# Transcriptome Analysis of Gene Expression Provides New Insights into the Effect of Mild Therapeutic Hypothermia on Primary Human Cortical Astrocytes Cultured under Hypoxia

**DOI:** 10.3389/fncel.2017.00386

**Published:** 2017-12-14

**Authors:** Mootaz M. Salman, Philip Kitchen, M. Nicola Woodroofe, Roslyn M. Bill, Alex C. Conner, Paul R. Heath, Matthew T. Conner

**Affiliations:** ^1^Biomolecular Sciences Research Centre, Sheffield Hallam University, Sheffield, United Kingdom; ^2^Institute of Clinical Sciences, University of Birmingham, Birmingham, United Kingdom; ^3^School of Life and Health Sciences, Aston University, Birmingham, United Kingdom; ^4^Sheffield Institute for Translational Neuroscience, University of Sheffield, Sheffield, United Kingdom; ^5^Research Institute of Health Sciences, Wolverhampton School of Sciences, University of Wolverhampton, Wolverhampton, United Kingdom

**Keywords:** hypothermia, astrocyte, Wnt signaling pathway, p53 signaling, apoptosis, hypoxia, MAPK

## Abstract

Hypothermia is increasingly used as a therapeutic measure to treat brain injury. However, the cellular mechanisms underpinning its actions are complex and are not yet fully elucidated. Astrocytes are the most abundant cell type in the brain and are likely to play a critical role. In this study, transcriptional changes and the protein expression profile of human primary cortical astrocytes cultured under hypoxic conditions for 6 h were investigated. Cells were treated either with or without a mild hypothermic intervention 2 h post-insult to mimic the treatment of patients following traumatic brain injury (TBI) and/or stroke. Using human gene expression microarrays, 411 differentially expressed genes were identified following hypothermic treatment of astrocytes following a 2 h hypoxic insult. KEGG pathway analysis indicated that these genes were mainly enriched in the Wnt and p53 signaling pathways, which were inhibited following hypothermic intervention. The expression levels of 168 genes involved in Wnt signaling were validated by quantitative real-time-PCR (qPCR). Among these genes, 10 were up-regulated and 32 were down-regulated with the remainder unchanged. Two of the differentially expressed genes (DEGs), p38 and JNK, were selected for validation at the protein level using cell based ELISA. Hypothermic intervention significantly down-regulated total protein levels for the gene products of p38 and JNK. Moreover, hypothermia significantly up-regulated the phosphorylated (activated) forms of JNK protein, while downregulating phosphorylation of p38 protein. Within the p53 signaling pathway, 35 human apoptosis-related proteins closely associated with Wnt signaling were investigated using a Proteome Profiling Array. Hypothermic intervention significantly down-regulated 18 proteins, while upregulating one protein, survivin. Hypothermia is a complex intervention; this study provides the first detailed longitudinal investigation at the transcript and protein expression levels of the molecular effects of therapeutic hypothermic intervention on hypoxic human primary cortical astrocytes. The identified genes and proteins are targets for detailed functional studies, which may help to develop new treatments for brain injury based on an in-depth mechanistic understanding of the astrocytic response to hypoxia and/or hypothermia.

## Introduction

Therapeutic hypothermia involves the controlled reduction of core temperature to minimize the secondary damage that occurs after a primary injury (Moore et al., 2011). Clinicians have been increasingly using therapeutic hypothermia to prevent or improve a wide range of neurological morbidities (Yenari and Han, 2012). The neuroprotective effect of therapeutic hypothermia has been confirmed in a number of clinical trials investigating the outcomes of patients suffering from neonatal hypoxia-ischemia (Gluckman et al., 2005; Shankaran et al., 2005) and cardiac arrest (Bernard et al., 2002). Hypothermia was reported to be neuroprotective in stroke patients (Yenari and Hemmen, 2010) but in human trials in patients suffering from a traumatic brain injury (TBI), a therapeutic role for hypothermia is controversial; different clinical trials have reported both positive and negative outcomes on patient recovery (Andrews et al., 2015; Lazaridis and Robertson, 2016).

The degree of cooling, duration of treatment and rate of rewarming of the brain vary between studies and trials. This may be one reason for the variable efficacy and side effects described (Carney et al., 2017). Current literature suggests that the optimal temperature for neuroprotection is 32–34°C (Yenari and Hemmen, 2010; Kim and Yenari, 2015). However, the optimal duration for hypothermia has not been defined therapeutically (Kim and Yenari, 2015). The precise mechanism of action underlying hypothermic intervention is still not understood owing to the complex mechanisms involved, which have been reported to include alterations in gene and protein expression, cellular signaling pathways and changes in cellular microstructure (Yenari and Han, 2012). It is also generally accepted that the early stages of brain edema formation (within the first 24–72 h) tend to be cytotoxic edema (with a swollen but intact astrocytic cell morphology), followed by a later vasogenic edema, characterized by disruption to the cellular structures (Unterberg et al., 2004). This may also underlie different outcomes following hypothermic intervention.

Astrocytes are the most abundant cell type of the brain and generally have a greater capacity than neurons to survive stresses (Jäkel and Dimou, 2017). Astrocyte cell function is critically affected by the lack of oxygen supply (hypoxia) to the brain which is usually associated with TBI (Conrad et al., 1999). It is vital for the brain to adapt and/or tolerate the changes in oxygen level with appropriate cellular mechanisms. One of the most extensively studied mechanisms of hypoxia under ischaemic conditions, is the role of hypoxia-inducible factor-1 (HIF-1) (Ziello et al., 2007; Loor and Schumacker, 2008). HIF-1 is a transcription factor, which has a pivotal protective role in enhancing the cellular adaptation to hypoxic stresses (Badawi et al., 2012). Activating HIF-1 results in transcriptional regulation of various genes associated with the adaption mechanism and promoting cellular survival in hypoxia. However, the specific intracellular mechanisms involved in such processes and following hypothermic intervention are not fully understood. As astrocytes are closely associated with neuronal function and regulation and remain mitotic, it would be therapeutically beneficial to understand their cellular pathophysiology following TBI and edema formation (Dienel and Hertz, 2005; Nedergaard and Dirnagl, 2005; Previch et al., 2016).

Therapeutic hypothermia has been widely studied for its neuroprotective effect, mainly against apoptosis and necrosis in neurons (Yenari and Han, 2012). More recent studies have assessed its effect on hypoxic and anoxic brain injuries (Shankaran et al., 2005). Additionally, hypothermia is considered to play an essential role in slowing or stopping some critical metabolic processes without resulting in death, referred to as “suspended animation” (Nozari et al., 2004). An incomplete understanding of the role of astrocytes and the complex interplay among signaling pathways (together with poorly defined treatment windows) are thought to be factors in the clinical failure of hypothermia (Andrews et al., 2015). Understanding the effects of therapeutic hypothermia in astrocytes may shed light on neuroprotective mechanisms and identify new drug targets and/or improved combined therapeutic approaches.

To gain insight into the complex mechanisms that mediate the effect of hypothermic intervention following ischemia/hypoxia, a comprehensive transcriptomic profiling of gene expression, using microarray analysis, was carried out and the resultant outcome further investigated using validation techniques at both the mRNA and protein level.

In this study, the transcriptome profile analysis of gene expression in primary human cortical astrocytes was analyzed to investigate the global transcriptional response in an *in vitro* cellular model of cytotoxic edema on primary human cortical astrocytes, with and without mild hypothermic intervention. A primary goal of the study was to identify the pathways and related functions that might be mediated by hypothermic intervention. Hence, the differential gene expression of human primary cortical astrocytes grown under hypoxic conditions (5% O_2_) for 6 h and the effect of 32°C hypothermic intervention after initial incubation for 2 h of hypoxia (hypoxia+hypothermia; for the total of 6 h) was investigated. These conditions and time points were selected to mimic hypoxia following brain injury and a typical clinical intervention following hypoxia after traumatic injury (Ma et al., 2012; Andresen et al., 2015). This is aimed at reducing early cytotoxic edema formation and the likelihood of surgical intervention.

Bioinformatic analysis has been used to identify pathways that could be involved in the effects of hypothermia. PCR profiling and ELISA were used to validate the microarray findings at the transcriptional and translational levels, respectively. Proteome profiling Array (PPA) has been used to investigate the effect of hypothermic intervention on human apoptosis-related proteins in hypoxic astrocytes.

The genes and proteins affected by hypothermic intervention following hypoxia could be targeted as potential candidates for functional validation and have potential translational value for reducing edema formation following TBI and/or stroke.

## Materials and methods

### Cell culture

Primary human cortical astrocytes (Sciencell, Cat. No. 1800) were plated on 75 cm^2^ culture flasks (Thermo Scientific Nunc Cell Culture Treated EasyFlasks) and cultured routinely in Astrocyte Medium (Sciencell; 1801) containing 1% fetal bovine serum (FBS, Sciencell Cat. No. 0010), 5 ml astrocyte growth supplement (AGS, Sciencell Cat. No. 1852) and 5 ml penicillin/streptomycin solution (P/S 1%, Sciencell Cat. No. 0503). The cells were then incubated either in humidified 5% (v/v) CO_2_ in air at 37°C for the normoxia work or in a controlled hypoxic atmosphere using a Coylab Hypoxia Chamber Glove Box, with a humidified airtight apparatus with inflow and outflow valves (into which a mixture of 95% N_2_ and 5% CO_2_ was flushed) for the hypoxia-related experiments. The effect of hypoxic intervention using the indicated chamber were validated by investigating the differential expression of Hypoxia-inducible factor 1-alpha (HIF-1α). The α subunit of HIF is considered to be the O_2_-sensitive subunit, constitutively destroyed during normoxia. However hypoxia is known to stabilize the expression of HIF-1α subunit, resulting in more HIF-1α activation and the subsequent HIF-1α mediated hypoxic response (Tanaka et al., 2009). The chamber was sealed to maintain the gas composition and kept at 37°C for experiments under normothermic conditions or 32°C for hypothermia-related work. Astrocytes were grown either under hypoxic conditions (5% O_2_) for 6 h (hypoxia) or under 32°C hypothermic intervention after initial incubation for 2 h of hypoxia (hypoxia+ hypothermia; for the total of 6 h). Astrocytes used for the experiments were at low (5 or below) passage number and the expression of AQP4 and GFAP (archetypal biomarkers for astrocytes) was regularly checked to ensure that they were not de-differentiated. Cellular proliferation and viability was determined by measuring the amount of ATP generated by viable cells using the CellTiter-Glo® luminescent cell viability assay (Promega, Cat. No. G7570) as per the manufacturer's instructions. The results indicated that cell viability levels were 92, 86.4, and 90.3% for normoxic control, 6 h hypoxia and 6 h hypoxia+hypothermia; respectively.

### RNA extraction, cDNA synthesis and purification, and quantitative real-time polymerase chain reaction

Total RNA was isolated from primary human cortical astrocytes with RNeasy plus mini kit (Qiagen; Cat. No. 74134). cDNAs were generated from 1 μg total RNA (or 8 μl from total RNA samples with less than 125 ng RNA) by SUPERSCRIPT III RNase H with oligo (dT)_18_ (Applied Biosystems; Cat. No. 18080400). The cDNAs were purified using the QIAquick PCR purification kit (Qiagen; Cat No. 28104) and multi-target RT-qPCR was then performed on a StepOnePlus™ Real-Time PCR System (Applied Biosystems) using purified cDNA samples to investigate gene expression levels of the investigated genes. The following Taqman assay IDs were used: *HIF-1*α (Hs00153153_m1) in addition to *PPIA* (Hs99999904_m1) and *CDKN1B* (Hs00153277_m1) which were used as housekeeping genes (Applied Biosystems) (Ayakannu et al., 2015; Salman et al., 2017; Tan et al., 2017). 2 μl containing 10 ng cDNA were used in the preparation of the 10 μl reactions using the TaqMan Universal PCR Master Mix (Applied Biosystems; Cat. No. 4324018). Samples were run for 50 cycles and results were analyzed using the 2^−ΔΔCt^ method (Schmittgen and Livak, 2008) and presented as relative gene expression normalized to the average cycle threshold for the two housekeeping genes.

### RNA preparation and quality control

Total cellular RNA was isolated as previously described in 2.2. To reduce interference in qPCR analysis caused by poor RNA quality, extracted RNA was assessed for its concentration, purity and integrity using a Nanodrop 1000 Spectrophotometer (Thermo Scientific) and an Agilent 2100 Bioanalyzer instrument (Agilent, Wokingham, UK). The RNA concentration was within the range 10–200 ng/μL; 260/280 nm absorbance ratio was ≥ 1.9; 260/230 nm ratio was ≥ 1.9; 28S/18S ribosomal ratio was ≥ 2; and RNA Integrity Number (RIN) was ≥ 9. These recommended conditions (Zhang et al., 2004) were met in order to consider any RNA sample for subsequent steps.

### Microarray

Microarray analysis was carried out using the Agilent one color methodology for low RNA input using G3 human gene expression 8 × 60 K v3 microarray (G4858A-072262), according to the manufacturer's instructions (Agilent). Briefly poly A spike–ins were prepared and added to 200 ng total RNA in a 3.5 μl reaction mix; quantified using NanoDrop spectrophotometer. T7 primer mix and first strand buffer were added and cDNA prepared. A transcription mix was then used to prepare cRNA with Cy3 labeled cytidine residues. Following purification and quantification of the reaction success, an appropriate quantity of these molecules was fragmented before being incorporated into a hybridization cocktail and applied to the microarray slide. The Agilent 8 × 60 design was used and samples distributed across the gasket slide upon which the array slide was placed and the hybridization assembly prepared. Following overnight incubation at 65°C with rotation at 10 rpm, the assembly was disassembled in a low stringency wash buffer (Agilent) and following further stringency washes the slide(s) were scanned in an Agilent C scanner. The resultant image files were further examined using Agilent Feature extraction software to produce the.txt files, which were interrogated for quality control and differential expression analysis using Agilent GeneSpring software.

### Microarray analysis

Gene Spring (v13.1.1 from Agilent) was used to determine a difference in transcript expression which was considered significant if a fold change (FC) ≥ 1.5 and a corrected *p*-value < 0.05 was determined on (*n* = 2) independent extractions from the cells on two individual microarray analyses. The full list of the up- and down- regulated genes following the comparison are provided. The list of up- and down-regulated genes was submitted to the bioinformatics and functional annotation tool provided by DAVID, version 6.7 (Huang da et al., 2009a,b). The KEGG pathway enrichment analysis then identified potential pathways that were associated with the mechanisms mediated by the hypothermic and/or hypoxic effects of astrocytic pathophysiology (http://david.abcc.ncifcrf.gov/). To minimize false positives among significantly-enriched functions, a false discovery rate (FDR) ≤ 0.05 (−log *P*-value = 1.33) was used to determine the probability that each biological function assigned to that data set was due to chance alone. KEGG pathways with enrichment score (ES) > 1.3 were considered significantly enriched.

Gene ontology (GO); the up-regulated and down-regulated genes identified from GeneSpring software analysis were submitted separately to DAVID, version 6.7 (Huang da et al., 2009a,b) and subsequent GO terms were identified using the same statistical parameters. GO terms and categorization of the up- and down-regulated genes according to their known molecular function following each of the indicated comparisons have been performed and for simplicity, various GO terms in broader Gene Ontology Biological Processes (GO_BP) categories were grouped as shown in Supplementary Table 1).

### Pathway-specific expression arrays (RT^2^ PCR profiler array)

cDNA was synthesized using the RT^2^ first strand kit (SABiosciences, Qiagen) from 500 ng total RNA and qPCR reactions were performed using RT^2^ SYBR® Green qPCR Mastermix (Qiagen; Cat. no. 330529); both were performed according to the manufacturer's instructions. An RT^2^ Profiler PCR array specific for Wnt signaling pathway plus (Qiagen, Cat. no. PAHS-043Y) and one specific for MAPK signaling pathway (Qiagen, Cat. no. PAHS-061Z) components were used to assess the transcript levels of 168 different genes using StepOnePlus™ Real-Time PCR System (Applied Biosystems). A full list of genes used in this study along with unigene ID, RefSeq numbers, gene symbol and description, are listed in Supplementary Tables 2, 3, respectively.

Raw C_T_ values were analyzed using the SAB PCR Array Data Analysis Web Portal online analysis software (http://pcrdataanalysis.sabiosciences.com/pcr/arrayanalysis.php). Samples were assigned to controls and test groups. C_T_ values were normalized based on an automatic selection from housekeeping genes (HKGs) panel of reference genes. The data analysis web portal autogenerates fold change/regulation using delta delta C_T_ method, in which delta C_T_ is calculated between gene of interest (GOI) and an average of HKGs, followed by delta-delta C_T_ calculations (delta C_T_ [hypoxia+hypothermia Group)-delta C_T_ (hypoxia Group)]. Fold Change (FC) is then calculated using 2∧ (-delta delta C_T_) formula by dividing the normalized gene expression [2∧ (-Delta C_T_)] in the hypoxia+hypothermia samples by the normalized gene expression [2∧ (-Delta C_T_)] in the hypoxia only samples (which is considered as a control for the analysis). Fold-change values greater than one indicate a positive or an up-regulation, and the fold regulation is equal to the fold-change. Fold change values less than one indicate a negative or down-regulation, and the fold-regulation is the negative inverse of the fold-change. All array internal quality controls were similarly calculated to indicate an absence of genomic contamination, optimal reverse transcription efficiency, and high sensitivity (SABiosciences online analysis software).

### Cell based ELISA measuring MAPK protein expression and activity levels

Primary human cortical astrocytes were pre-treated with hypoxia or hypoxia+hypothermia as indicated in 2.1. Total and phosphorylated protein levels of p38 and JNK MAPK were determined using cell based ELISA kit (RayBio® Cell Based, Cat No. CBEL-ERK-SK, Ray Biotech Inc., Norcross, GA, USA) as per manufacturer's instructions.

### Proteome profiling

Proteome Profiler™ Human Apoptosis Array Kit (R&D Systems; Catalog # ARY009) was used to analyse the expression profiles of apoptosis-related proteins. Protein was extracted using CelLytic™ as described above). The total protein concentration was determined using Pierce™ BCA Protein Assay Kit. Each proteome profiler membrane was then incubated with 250 μg of protein lysate per each array and diluted to the final volume of 1.25 mL of Array Buffer 1, according to the manufacturer's instructions. The HRP-conjugated streptavidin provided in the kit was replaced with IRDye® 800CW Streptavidin (LI-COR, 926-32230) and it was diluted at 1:2000 using the array Buffer 2/3 (R&D Systems, ARY009). All the following steps were performed according to the manufacturer's instructions. The arrays were scanned with LI-COR Odyssey® Infrared Imaging System and quantified with Image Studio™ software (LI-COR) to determine the relative amount of the specific proteins. A full list of the investigated proteins is provided in Supplementary Table 4.

### Statistical analysis

Data are presented as a fold-change normalized to the experimental control. Microarray analysis, RT-qPCR expression, ELISA and Proteome Profiling data were found to be nonparametric in distribution using the Shapiro-Wilk test, so a Kruskal–Wallis analysis with Conover– Inman *post hoc* test was used to identify significant differences (*P* ≤ 0.05) using StatsDirect 3 software.

## Results

### Effect of hypoxia on primary human cortical astrocytes

**Figure 1:**
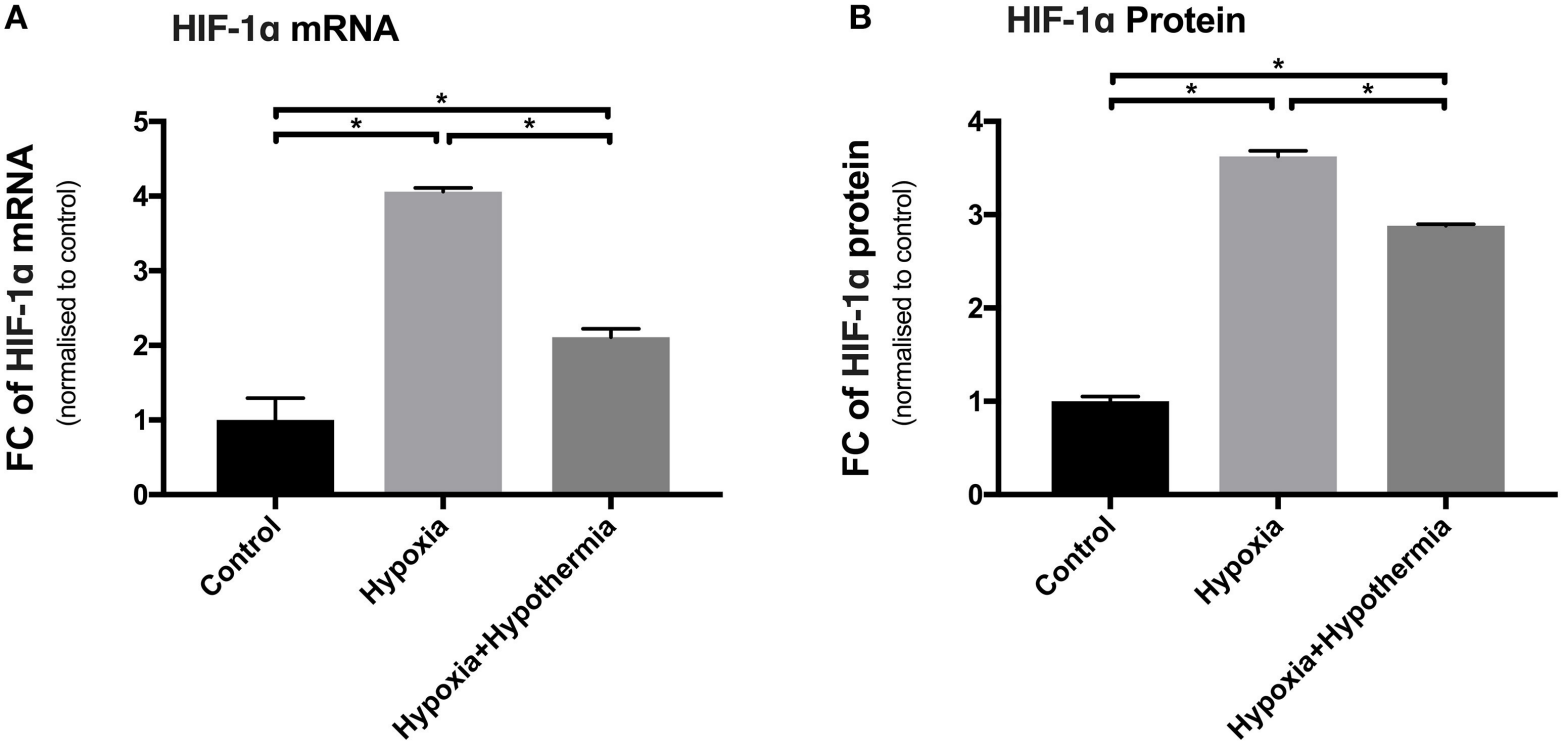
HIF-1α mRNA and protein expression levels in cultured primary human astrocytes following hypoxia or hypoxia+hypothermia. Data shown include mean fold change in expression (± S.E.M.) of cells treated with 5% oxygen for 6 h with no temperature change (Hypoxia) or with 32°C hypothermic intervention after initial incubation for 2 h of hypoxia for a total of 6 h (Hypoxia + Hypothermia) compared with untreated normoxic, normothermic astrocytes (Control). Each bar represents the Mean ± S.E.M. for each of the conditions. Kruskall-Wallis with Conover-Inman *post-hoc* analysis tests were used to identify significant differences between samples using StatDirect 3 software. ^*^Represents statistical significance (*p* < 0.001). **(A)** Shows RT-qPCR data (*N* = 5) using Taqman probes and 2^∧^−ΔΔCT analysis normalized to the two housekeeping genes, PPIA and CDKN1B. **(B)** Shows Proteome Profiling Array data (*N* = 3). FC, Fold change.

To validate the impact of hypoxia on primary human cortical astrocytes, the expression of HIF-1α was investigated at the mRNA and protein level using RT-qPCR and as part of the proteome profiling array, respectively. This was measured with and without hypothermic intervention and the results presented as fold change compared with the normoxic control. The results in Figure 1A show that hypoxia caused a significant upregulation of HIF-1α mRNA (4.05-fold ± 0.05; *p* < 0.0003) compared to untreated control astrocytes. Hypothermic intervention caused a significant reduction in the hypoxia-induced upregulation by 1.94-fold ± 0.11(*p* < 0.01) but the levels were still significantly higher than the untreated control astrocytes (2.11-fold ± 0.11; *p* < 0.01).

Hypoxia led to a significant upregulation in HIF-1α protein expression (3.62-fold ± 0.06; *p* < 0.0014) compared to untreated control astrocytes (Figure 1B). Moreover, hypothermia reduced the hypoxia-induced expression of HIF-1α protein by 0.74-fold (±0.018; *p* < 0.0017) but the level was still significantly higher than untreated control cells (2.88-fold ± 0.05; *p* < 0.0034). These results confirm a measurable effect of hypoxia treatment at both the mRNA and protein level in primary human astrocytes (Figure 1B).

### Gene expression profiles using microarray analysis

Transcriptional profiling of primary human cortical astrocytes was measured using Agilent single color G3 technology, under hypoxia or hypoxia+hypothermia. Comparison of hypoxia treatment of astrocytes, with and without hypothermia intervention, revealed 411 differentially regulated genes (209 down-regulated and 202 up-regulated) following GeneSpring analysis (A full list of the up- and down-regulated genes is provided in Supplementary Tables 5A,B).

### KEGG analysis of functional pathways

To obtain biological information for describing the molecular mechanisms and regulatory networks involved in hypothermic intervention of hypoxia in human astrocytes, the microarray data were assessed using KEGG enrichment pathway analysis (key results summarized along with *p*-value and fold enrichment in Table 1).

**Table 1:** Comparison of KEGG pathway analysis of hypoxia+hypothermia vs. hypoxia ranked according to *p*-value and fold enrichment for differentially expressed genes in astrocytes.

**KEGG pathways**	***p*-value**	**Fold enrichment**
1. Hedgehog signaling pathway	1.40E-03	7.1
2. Wnt signaling pathway	7.00E-03	3.5
3. p53 signaling pathway	1.8E-2	4.9

A primary focus of this study was to analyse hypoxia+hypothermia versus hypoxia alone. The subsequent KEGG analysis indicated that these genes were mainly enriched in three major pathways: Hedgehog, Wnt and p53 signaling pathways; which were inhibited following the hypothermic intervention.

**Figure 2:**
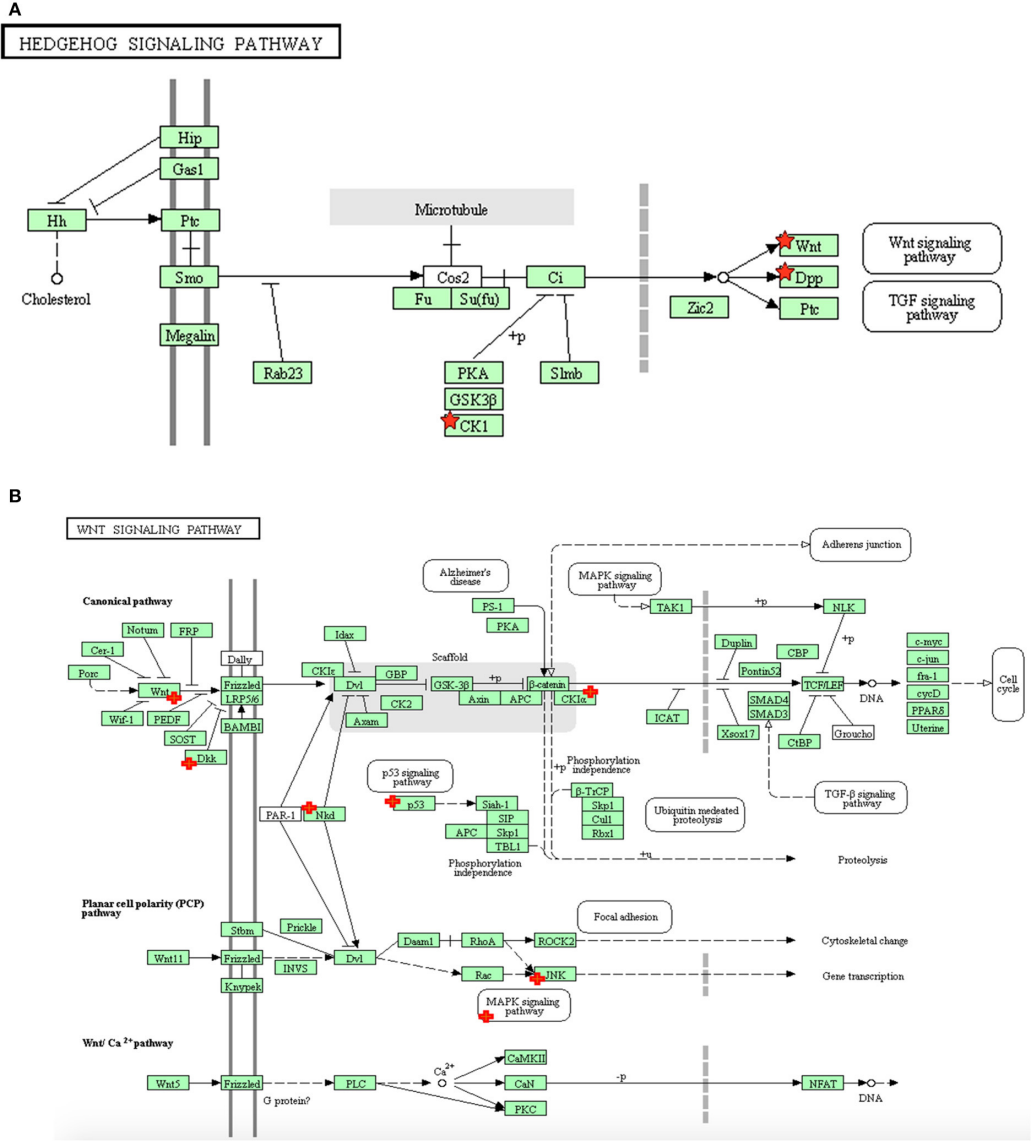
The distribution of differentially expressed genes in astrocytes following hypoxia+ hypothermia vs. hypoxia: **(A)** Within the Hedgehog signaling pathway, **(B)** Within the Wnt signaling pathway in primary human cortical astrocytes being cultured under hypoxic conditions (5% O_2_) for 6 h with or without hypothermic intervention (32°C after 2 h; maintaining hypoxia for 6 h) (adapted from KEGG website: www.genome.jp/kegg). The significant up- and down-regulated genes, indicated with red + signs, were identified using DAVID/KEGG enrichment analysis of microarray data (*n* = 2).

The pathway most enriched from the hypoxia+hypothermia group compared with hypoxia alone (Table 1) was the Hedgehog signaling pathway. The microarray results also suggest that the Hedgehog signaling pathway mediates its essential effects via the downstream involvement of the Wnt signaling pathway in the transcriptional regulation process (Figure 2A). The Wnt signaling pathway alone was the second highest enriched pathway (3.5 fold; *p* = 7.00E-03) (Table 1; Figure 2B). Additionally, further GO term analysis indicated that the Wnt receptor signaling pathway is the highest enriched biological process for the identified differentially expressed genes (Supplementary Table 1). Therefore, the differential transcriptional regulation of the Wnt pathway in human astrocytes was investigated under hypoxia with or without the hypothermic intervention at the transcript level and further validating it at the gene level using pathway specific RT^2^ Profiler PCR Arrays. Treating astrocytes with hypothermia did not have an effect on the Hedgehog or Wnt signaling pathways; and the results show that hypothermia may mediate its effects mainly via interfering with cell adhesion and adherens junction compared to untreated normoxic normothermic astrocytes (E.score 4.9; *p*-value = 4.60E-02).

### Validation of hypothermic-intervention effects on wnt and p53 pathways using pathway specific RT^2^ profiler PCR arrays

The expression of 168 genes known to be involved in the Wnt signaling pathway were examined to investigate whether basal differences in transcription play a role in mediating the effect of hypothermia following hypoxia on astrocytes using RT^2^ Profiler PCR Arrays for Wnt signaling pathway molecules. The results indicate the significant differential expression of 42 genes on the array. Among them, 32 genes were significantly down-regulated whilst the expression of only 10 genes were significantly induced (Table 2).

**Table 2:** Fold regulation comparison and *p*-value for genes within the human Wnt signaling and related pathways in human astrocytes under hypoxia with or without hypothermic intervention (a full list of all the investigated genes is provided in Supplementary Tables 2,3).

**Unigene ID**	**Gene symbol**	**Fold change**	***p*-value**
Hs.213424	SFRP1	−6.3451	0.005
Hs.282410	CALM1	−6.2696	<0.001
Hs.121540	WNT10A	−5.7345	<0.001
Hs.584775	LRP6	−4.0256	0.011
Hs.149504	WNT9A	−4.0151	<0.001
Hs.94234	FZD1	−3.9556	0.002
Hs.138211	MAPK8	−3.9479	<0.001
Hs.292156	DKK3	−3.9375	<0.001
Hs.40735	FZD3	−3.9292	0.049
Hs.516646	CREB1	−3.8994	<0.001
Hs.463978	MAP2K6	−3.4831	0.044
Hs.712929	CTNNB1	−3.2242	<0.001
Hs.23348	SKP2	−3.1765	0.03
Hs.194698	CCNB2	−3.1619	0.025
Hs.523852	CCND1	−3.1179	0.03
Hs.643085	WNT5A	−2.8536	0.027
Hs.445884	WNT3	−2.5029	0.002
Hs.654934	DAAM1	−2.4984	0.002
Hs.258575	WNT2B	−2.4898	0.002
Hs.512714	WNT7B	−2.4728	0.002
Hs.485233	MAPK14	−2.4269	0.037
Hs.644231	ETS2	−2.1205	<0.001
Hs.437460	p53	−2.08	<0.001
Hs.19192	CDK2	−2.0198	<0.001
Hs.408528	RB1	−2.0135	<0.001
Hs.234521	MAPKAPK3	−1.9963	<0.001
Hs.696684	JUN	−1.9679	<0.001
Hs.248164	WNT1	−1.9091	<0.001
Hs.592510	ATF2	−1.8732	<0.001
Hs.57732	MAPK11	−1.8633	<0.001
Hs.306051	WNT5B	−1.7533	0.005
Hs.145605	MAP3K2	1.8447	0.004
Hs.390428	MAP3K4	1.8914	<0.001
Hs.514012	MAP2K3	1.9496	<0.001
Hs.643566	MAPKAPK2	1.9993	<0.001
Hs.40499	DKK1	2.0323	<0.001
Hs.433332	LAMTOR3	2.0336	<0.001
Hs.202453	MYC	2.0639	<0.001
Hs.649965	MEF2C	2.1279	0.001
Hs.326035	EGR1	3.1509	0.025
Hs.627078	CXADR	3.2143	0.002

The RT-qPCR array platform has an embedded analytical score feature that is used to indicate the activity status of the investigated pathway. A positive score indicates activation of the pathway, whilst a negative score indicates repression. The results indicated that the activity score was −0.721 (*p* < 0.01) which reveals a significant inhibition of the Wnt signaling pathway following hypothermic intervention on astrocytes under hypoxia compared to astrocytes being cultured under hypoxia only (data not shown). This agrees with the findings from the microarray analysis (See Supplementary Table 1).

### Comparing protein expression of total and phosphorylated p38 and JNK proteins using ELISA on human primary cortical astrocytes

Transcriptional changes do not always reflect translational alterations due to many factors such as the efficiency of translation and the stability of the generated protein (Cenik et al., 2015). p38 and JNK are key Wnt signaling pathway proteins that mediate responses to a variety of cell stresses including ischemia, stress stimuli (such as cytokines), ultraviolet irradiation, heat shock and osmotic shock (Mielke and Herdegen, 2000; Wallace et al., 2012). p38 and JNK are also known to be altered in response to hypoxia in both neurons (Liu et al., 2009) and astrocytes (Roy Choudhury et al., 2014; Jeong et al., 2017). It was also shown in these studies that the activation status of these proteins may be as relevant as translational changes. This study therefore used these examples to interrogate the microarray findings at the protein level. The transcriptional results for these two genes showed a significant inhibition at the mRNA level using both the microarray (0.02 ± 0.007; *p* < 0.05; 0.23 ± 0.003; *p* < 0.05) and RT-qPCR (Table 2). To assess the translational profile and activity status of these stress kinases following hypoxia with or without hypothermic intervention, the total and phosphorylated p38 and JNK protein expression levels were measured using the cell based sandwich ELISA described in the methods.

**Figure 3:**
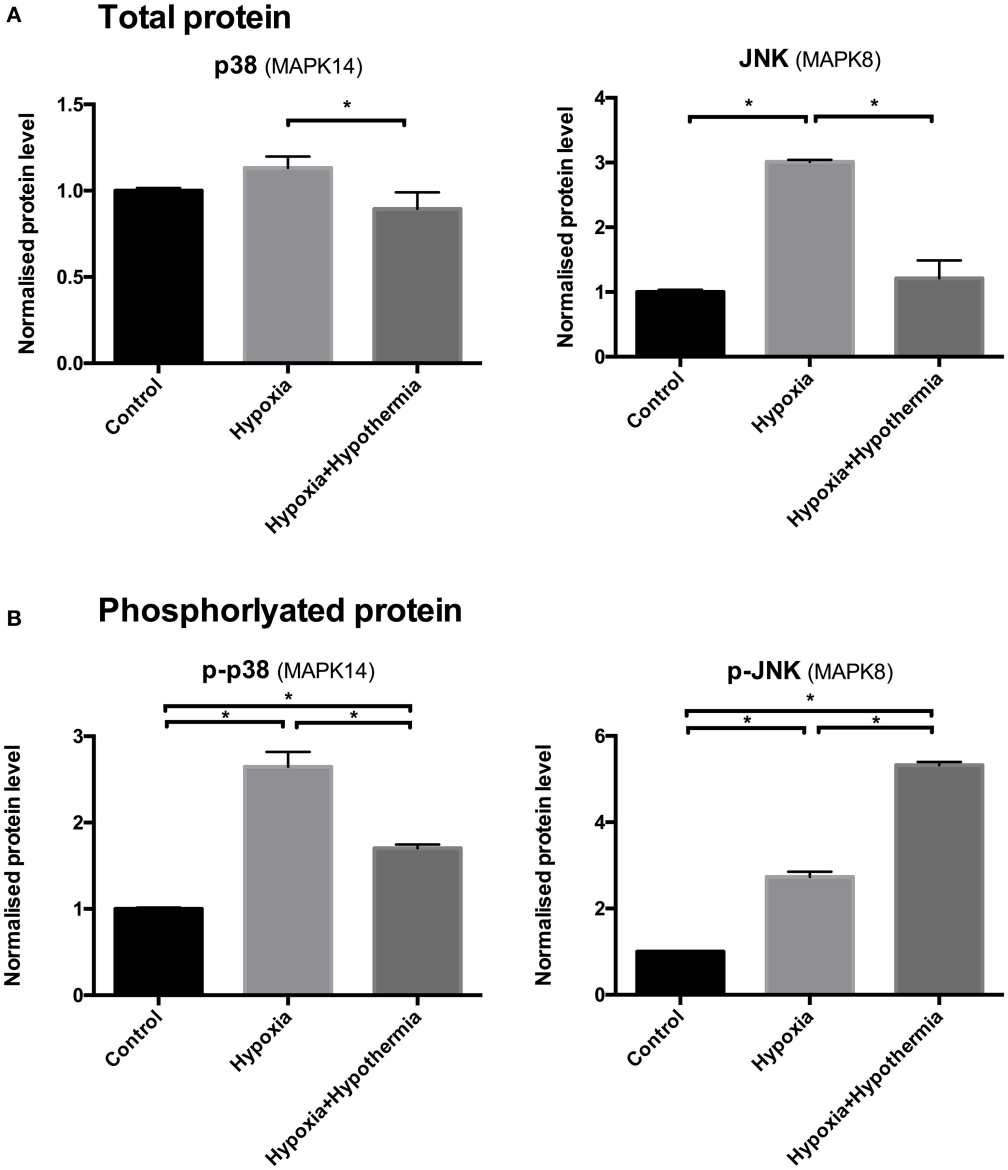
p38 and JNK **(A)** total and **(B)** phosphorylated protein levels in cultured human astrocytes using sandwich ELISA. Data are mean fold changes in expression (± S.E.M.) of cells incubated at 5% O_2_ for 6 h (“hypoxia”) and hypothermic intervention after 2 h (32°C; maintaining hypoxia for 6 h) (“hypoxia+hypothermia”) normalized to normoxic normothermic (“Control”) astrocytes. Each bar represents the mean ± S.E.M. for each of the conditions (*n* = 4). Kruskal-Wallis with Conover-Inman *post-hoc* analysis tests were used to identify significant differences between samples. ^*^Represents statistical significance (*p* < 0.05).

There was no significant difference in the total p38 (MAPK14) protein levels following hypoxia, compared to the untreated control (*p* = 0.23). Hypothermic intervention caused a significant decrease in the pan p38 levels following hypoxia by 21% (±0.09; *p* = 0.03) but not in comparison to the untreated control where there was no significant change (*p* = 0.2). Regarding JNK (MAPK8); 6 h hypoxia causes a significant upregulation (3.01-fold ± 0.02; *p* < 0.0003) in regard to the untreated control but the hypothermic intervention reduced the hypoxia-induced upregulation and restores the pan-JNK levels to a level where there is no detected statistical difference to the untreated control (*p* = 0.46) (Figure 3A).

However, 6 h hypoxia caused a significant 2.64 fold ± 0.17 (*p* = 0.0003) increase in the phosphorylated p38 (p-p38) protein levels compared to control (Figure 3B), indicating that p-p38 is rapidly activated in primary human astrocytes in response to hypoxia. Hypothermic intervention after 2 h significantly down-regulated the hypoxia-mediated increase in p-p38 levels by 35% ± 0.04 (*p* = 0.04). Despite the reduction following the hypothermic intervention; the levels were still significantly higher compared to the untreated control (1.7 fold ± 0.01; *p* < 0.004). 6 h hypoxia causes a significant increase in p-JNK protein levels (2.73-fold ± 0.11; *p* < 0.011) compared to untreated control (Figure 3B). Moreover, hypothermic intervention causes a further increase in p-JNK protein levels compared to untreated control and astrocytes being cultured for 6 h under hypoxia (5.32-fold ± 0.07; *p* < 0.0001 and 2.59-fold ± 0.08; *p* = 0.008); respectively.

### Validation of the effect of hypothermic intervention on apoptosis and p53 signaling pathways in hypoxic astrocytes

Table 3 shows that hypothermic intervention causes significant downregulation of 18 proteins, while only significantly upregulating survivin at the protein level (*P* < 0.05). Transcription levels were determined by reanalysing the MA data for each of these 19 individual genes to determine the transcript expression level. In addition, the translational and transcriptional expression FC was calculated by dividing the gene or protein expression level in hypoxia/hypothermia by its corresponding hypoxia value. The transcriptional data show that there was a significant downregulation of BCL2 Associated X (Bax) (*P* = 0.023), Bcl-2-like protein 1 (Bcl-x) (*P* = 0.029), claspin (*p* = 0.03), Fas-associated protein with death domain (FADD) (*P* = 0.034), HIF-1α (*P* = 0.023), Heat shock proteins [HSP; including HSP32 (*P* = 0.006), HSP27 (*P* = 0.042), HSP70 (*P* = 0.006)], Serine protease HTRA2 (HTRA2) (*P* = 0.039), and Cyclin Dependent Kinase Inhibitor 1B (CDKN1B) (*P* = 0.04) transcript expression level. While there was no significantly detected change at the transcript level of Cyclin Dependent Kinase Inhibitor 1A (CDKN1A) (*P* = 0.162), Cleaved Caspase-3 (CASP3) (0.248), Baculoviral IAP repeat-containing protein 3 (cIAP-2) (*P* = 0.468), Clusterin (*P* = 0.163), Rad17 (not detected) and SMAC/Diablo (*P* = 0.239). Conversely, the transcriptional result showed that livin and survivin were the only transcripts that show a significant upregulation at the transcriptional level (*P* = 0.003 and 0.001; respectively). Survivin; but not livin, showed a similar trend at the protein data.

**Table 3:** Fold regulation comparison for significant proteins within human p53 and apoptosis signaling and related pathways and their corresponding gene expression from the microarray data analysis.

**Target name**	**Gene fold change ± S.E.M**.	**Protein fold change ± S.E.M**.
Bax	0.15^*^ ± 0.03	0.43^*^ ± 0.07
Bcl-x	0.24^*^ ± 0.21	0.36^*^ ± 0.17
Cleaved Caspase-3	–	0.73^*^ ± 0.12
cIAP-2	NS	0.55^*^ ± 0.07
Claspin	0.32^*^ ± 0.27	0.84^*^ ± 0.08
Clusterin	NS	0.56^*^ ± 0.19
FADD	0.11^*^ ± 0.05	0.82^*^ ± 0.05
HIF-1α	0.06^*^ ± 0.01	0.79^*^ ± 0.02
HO-1/HMOX1/HSP32	0.38^*^ ± 0.03	0.54^*^ ± 0.05
HSP27	0.38^*^ ± 0.02	0.78^*^ ± 0.13
HSP70	0.4^*^ ± 0.01	0.71^*^ ± 0.1
HTRA2/Omi	0.35^*^ ± 0.06	0.49^*^ ± 0.19
Livin	2.17^*^ ± 0.02	0.47^*^ ± 0.09
p21/CIP1/CDKN1A	NS	0.45^*^ ± 0.21
p27/Kip1	0.35^*^ ± 0.03	0.59^*^ ± 0.27
Phospho-p53 (S15)	0.43^*^ ± 0.01	0.67^*^ ± 0.14
Phospho-Rad17 (S635)	–	0.65^*^ ± 0.02
SMAC/Diablo	NS	0.60^*^ ± 0.25
Survivin	5.77^*^ ± 0.49	1.41^*^ ± 0.12

*35 human apoptosis-related proteins closely associated with the Wnt signaling pathway were investigated using a Proteome Profiling array. Data shown include mean fold change (± S.E.M.) of significantly altered proteins in astrocyte cell lysates treated with 5% O_2_ for 6 h with no temperature change (hypoxia) or with a 32°C hypothermic intervention after initial incubation for 2 h of hypoxia (hypoxia+hypothermia; for a total of 6 h). Fold change (FC) was obtained by dividing the mean of the expression level in hypoxia+hypothermia by the mean of its corresponding hypoxia expression level. The data represent a comparative analysis for selected significantly expressed proteins (normalized to array control) and their corresponding genes (normalized to array signal) and in hypoxia+hypothermia and hypoxia. The results are presented as mean ± S.E.M. for microarray analysis (n = 2) and proteome profiler analysis (n = 3). Kruskal-Wallis with Conover-Inman* post hoc *analysis was used to identify significant differences between samples (*

* *P < 0.05, NS = not significant, – not detected in the microarray analysis)*.

It is notable to mention that proteome profiling array investigated three different sites of Phosphorylated-p53 protein at (S15), (S46), and (S392) and it was only the Phospho-p53 (S15) that showed a significant downregulation at the protein level, which could indicate the initial effect of hypothermia might be mediated through affecting the phosphorylation (and hence the activation) of different residues within the p53 protein.

## Discussion

Brain injuries such as TBI and stroke are usually associated with a state of hypoxia that results in glial activation (Kou and VandeVord, 2014). Glial cells, in particular astrocytes, play a major role in maintaining CNS homeostasis and protection from various stresses, by providing metabolic and structural support (Bélanger and Magistretti, 2009). Hypoxia can disrupt astrocyte homeostasis leading to cell swelling (Liang et al., 2007). This cytotoxic edema is one of the main deleterious effects of TBI, in some cases causing brain damage and death. Hypothermia has been shown to have a neuroprotective effect against glial activation associated with brain injury and cerebral hypoxia (Karnatovskaia et al., 2014). The therapeutic use of mild hypothermia is well established in animal models but it has a limited application in humans, due to the poorly defined mechanism of action (Yenari and Han, 2012). Identifying the mechanistic consequences of therapeutic hypothermia in primary human astrocyte cultures will help to elucidate the complex response in the central nervous system, as it provides a more physiologically relevant model compared to the most commonly used rodent cellular and/or animal models. This will also be important in terms of validating new drug targets due to the fact that oxygen differentially regulates gene expression in a cell-specific and species-specific manner (Mense et al., 2006).

### HIF-1α

It is well known that hypoxia induces many signaling pathways in astrocytes such as HIF-1α, NF-κB, p53, cAMP, CREB and c-jun (Rosenberger et al., 2001), all of which could affect astrocyte survival (Vangeison and Rempe, 2009). The results in this study show that hypothermia significantly reduced the mRNA expression of HIF-1α, CREB1, JUN and TP53, which was also confirmed at the protein levels for HIF-1α.

Ischemia is one of the main causes of cerebral hypoxia. HIF-1α is a protein that plays a major role in oxygen homeostasis in body tissues including the brain and is considered an oxygen sensor and it is well established that oxygen tension plays a major role in its activation (Semenza, 1999; Tanaka et al., 2009; Ding et al., 2010). HIF-1α abundance is very low under normoxic conditions, as it rapidly undergoes degradation via ubiquitination and proteasome degradation (Jaakkola et al., 2001). It has been shown that this protein is up-regulated during cerebral hypoxia due to an inhibition of the degradative process (Mu et al., 2003). This upregulation is associated with harmful outcomes such as blood–brain barrier (BBB) disruption or cell death mediated by different target genes (Kaur et al., 2006). It is well-known that therapeutic hypothermia significantly decreases HIF-1α neo-synthesis following hypoxia, inhibiting the gene expression for adaptation to hypoxia in both *in vivo* and *in vitro* models (Tanaka et al., 2009). The results (Figure 1) confirmed these findings by showing that hypoxia significantly upregulated HIF-1α at both gene and protein levels. This also serves as a useful control, validating our cellular model of *in vitro* cytotoxic edema. The hypothermia induced significant down-regulation of HIF-1α mRNA and protein could be part of the mechanism by which hypothermia mediates its neuroprotective effect on astrocytes.

### Microarray analysis

The microarray analysis identified 411 differentially expressed genes following hypothermic treatment of human astrocytes cultured under hypoxia. The subsequent KEGG analysis indicated that these genes were mainly enriched in three major pathways: Hedgehog, Wnt and p53 signaling pathways; which were inhibited following hypothermic intervention. The pathway most affected from the hypoxia+hypothermia group compared with hypoxia alone was the Hedgehog signaling pathway. The evolutionarily conserved Hedgehog (Hh) complex pathway is fundamental for embryonic development and exhibits essential roles in cellular survival, and adult tissue maintenance, regeneration and renewal (Mimeault and Batra, 2010). It mediates its effect via the secreted Hh proteins that act in a concentration- and time-dependent manner to initiate a series of cellular responses through transcriptional regulation (Mimeault and Batra, 2010). Despite being the pathway with the highest enrichment score, the microarray results also suggested that the Hedgehog signaling pathway mediates its essential effects via the downstream involvement of the Wnt signaling pathway in the transcriptional regulation process (Figure 1A, Table 2).

The Wnt signaling pathway on its own (Figure 1B, Table 2) had the second highest enrichment score. Interestingly, the idea of Wnt signaling involvement is further supported by the results from the GO term analysis, which indicated that the Wnt receptor signaling pathway and its calcium modulating pathway were the highest enriched biological processes for the identified genes (Supplementary Table 1). Moreover, the remaining upregulated differentially expressed genes identified within the Wnt signaling were mainly associated with direct antagonism of the canonical Wnt signaling mainly including Dickkopf-related protein 1 (DKK1). DKK1 antagonizes canonical Wnt signaling by inhibiting the low-density lipoprotein-related receptor (LRP5/6) interaction with Wnt and by forming a ternary complex with the transmembrane protein kringle domain-containing transmembrane protein 1 (KREMEN1) that promotes internalization of low-density lipoprotein receptor (LRP5/6) (Mao et al., 2002). DKK1 was also reported to antagonize the Wnt/β-catenin pathway through a reduction of β-catenin and an upregulation in octamer-binding transcription factor 4 (OCT4) expression (Ou et al., 2016).

### Wnt signaling

Analysis of the microarray data identified inhibition of the Wnt signaling pathway following hypothermic intervention on astrocytes under hypoxic conditions compared with hypoxia alone. Conversely analysis of the microarray data comparing astrocytes cultured under hypoxia with the untreated normoxic control cells suggested that hypoxia caused an activation of Wnt signaling pathway in agreement with earlier findings (Mazumdar et al., 2010).

The RT-qPCR data confirmed our microarray findings regarding inhibitory effects of hypothermic intervention on Wnt signaling for hypoxic astrocytes. The hypothermia-mediated inhibition of Wnt signaling in the RT-qPCR data may be mediated through several possible mechanisms such as the direct inhibition of a number of Wnt signaling elements including Wnt1, Wnt2B, Wnt3, Wnt5A, Wnt7B, Wnt9A and Wnt10A or through the inhibition of key downstream components of the canonical Wnt signaling pathway such as β-catenin (CTNNB1) as shown in the RT-qPCR data. Wnt inhibition could be also mediated through the direct activation of direct Wnt antagonists such as DKK1 (Niida et al., 2004) which was shown by RT-qPCR to be significantly upregulated.

Wnt inhibition could also be mediated through the inhibition of other related genes or closely linked signaling pathways. The RT-qPCR data show that there is an inhibition of secreted frizzled-related protein 1 (SFRP1). In general, SFRPs function as soluble Wnt signaling modulators, and they are known to be activated by hypoxia (Fukuhara et al., 2002). The binding of SFRPs and Frizzled (FZD) receptors; a family of G protein coupled receptors within the Wnt pathway; is thought to form a complex that inhibits Wnt signaling (Chim et al., 2007).

There was a marked inhibition of FZD1 and FZD3 receptors. The majority of FZD receptors were linked to the β-catenin canonical signaling pathway that promotes the activation of Wnt genes and Disheveled proteins and the decrease of glycogen synthase kinase 3(GSK3) kinase activity. They are also linked to signaling pathways involving PKC and calcium influxes where PKC is suggested to be needed for Wnt mediated GSK3 kinase inhibition (Cook et al., 1996). The inhibition of FZD1 and FZD3 in the experiments reported here could be due to Wnt3, Wnt1 and Wnt2b for FZD1 or Wnt5a for FZD3 (Blagodatski et al., 2014). Moreover, there was also a significant downregulation of Disheveled-associated activator of morphogenesis 1 (DAAM1), which plays a role in cell adhesion, cytokinesis and polarity through its binding to Disheveled (Dvl) and Rho, where it could play a role as a scaffolding protein that enhances Rho-GTP formation (Habas et al., 2001). It also has an important role in mediating the cellular polarity via directing the nucleation and elongation of new actin filaments, which could result in changes in astrocyte morphology (Ang et al., 2010; Ju et al., 2010).

### MAPK signaling

MAPK pathways are important intracellular signal transduction pathways involved in the protective response of cells to hypoxia and they play a key role in signaling cascades and within the Wnt pathway (Bikkavilli and Malbon, 2009). Intracellular signaling cascades are of interest after TBI because they are involved in the regulation of cellular repair, plasticity and homeostatic functions, which are markedly altered in response to TBI (Huang et al., 2009c). It is now well documented that the MAPK pathway represents a highly conserved upstream mechanism for transducing stress signals in eukaryotic cells (Guyton et al., 1996; Garrington and Johnson, 1999; Mielke and Herdegen, 2000).

The results from this study indicated that hypothermia significantly inhibits the MAPK pathways for p38 and JNK at both total protein and mRNA level, compared to hypoxia-induced elevated levels. Hypoxia caused a significant upregulation of the phosphorylated forms of p38 and JNK, which are known to be activated soon after TBI in astrocytes *in vitro* (Rosenberger et al., 2001; Wallace et al., 2012), and temperature markedly altered these responses (Huang et al., 2009c). Additionally, the results reported in this study show that hypothermia induced further activation of the phosphorylated forms of JNK, but not p38; these results for the increased phosphorylation of JNK but not p38 are in agreement with earlier reports that showed increased phosphorylation of JNK in cortical and hippocampal samples in a rat fluid-percussion model of TBI (Otani et al., 2002) and that hypothermia protects against TBI by the early inhibition of JNK activation and subsequent prevention of apoptosis in astrocytes (Ma et al., 2012).

Hypoxia and oxidative stress are typical physiological factors that affect the MAPK signaling pathway and, in particular, p38 (Conrad et al., 1999). The results here show a significant inhibitory effect of hypothermia on p38 activation, mRNA and total protein levels. This inhibition of the phosphorylated p38 protein occurred despite the significant upregulation at the mRNA level of known p38 activators such as MAPK3K4 (Takekawa et al., 1997), MAP2K3 (Raingeaud et al., 1996), and MAPKAPK2 (Rousseau et al., 2002; Tollenaere et al., 2015). Hypothermia mediated suppression of p38 activation suggests that hypothermia could mediate part of its neuroprotective effect via inhibiting inflammation (Diestel et al., 2009), cellular death and apoptosis (Yang et al., 2010). Thus, there is some indication that p38 inhibition could be a promising therapeutic target for hypothermia-mediated anti-inflammatory effects and for reducing brain injury and neurological pathology during the acute phase of cytotoxic edema (Barone et al., 2001).

The JNK signaling cascade plays a central role in the CNS including the regulation of apoptosis and regeneration of both neuronal and glial cells (Herdegen et al., 1997). The activation of JNK signaling can be induced by various cytotoxic stresses e.g., inflammatory cytokines, ischemia/hypoxia, and thermal shock (Huang et al., 2009c; Wallace et al., 2012). The results suggest that hypothermia modulates the early transient phase of hypoxia-mediated JNK stimulation that could regulate astrocytic survival which is in agreement with previous findings from the work of Ventura et al. (2006). The increase in activation of phosphorylated JNK protein agrees with the RT-qPCR results in this study that showed a significant upregulation of MAP3K2 (Cheng et al., 2000) and MAP3K4 (Takekawa et al., 1997), which are also known to be involved in JNK activation.

Hypothermia is known to cause a significant stimulation of JNK signaling intermediates (Lotocki et al., 2006). Linking these data with the observation of inhibited c-jun at the mRNA level from RT-qPCR suggests that hypothermia could result in the disruption of the JNK–c-Jun signaling pathway (Tournier et al., 2000; Noguchi et al., 2001; Kögel et al., 2005; Lotocki et al., 2006). Collectively, the present study suggests that hypothermia could mediate part of its neuroprotective effects via the downregulation of JNK levels following the oxidative-mediated insult on astrocytes.

### Apoptosis

Cellular death following ischemia and cerebral edema happens either by necrosis, which represents the premature death of the damaged cells or as part of the cascade of programmed cell death, or apoptosis (Frink et al., 2012). Apoptotic cell death is the most important mechanism contributing to cell loss following head injury and therefore the modulation of apoptotic cell death after hypoxia through hypothermia has been investigated (Ma et al., 2012).

Previous reports have suggested a role for hypothermia in reducing ischemia-mediated apoptosis and improving cell survival in animal models via decreasing p53 expression (Zhang et al., 2010). Additionally, hypothermia causes a reversible, p53-mediated cell cycle arrest in cultured fibroblasts (Matijasevic et al., 1998). This study showed a significant inhibition of p53 gene expression, which was confirmed by the protein data. This may suggest that a mechanism of action of hypothermia could be suppression of the early events of intrinsic apoptosis. These data could be linked to the significant inhibition of genes belonging to the highly-conserved cyclin family. The results demonstrated that hypothermia causes a significant downregulation of CCNB2, CCND1 and RB1 genes in addition to significant inhibition of Cyclin-dependent kinase 2 (CDK2). Each of these have been shown to play important roles in the regulation of the cell cycle and their inhibition may play a role in altered modulation of the cell cycle, leading to apoptotic cell death (Senchenko et al., 2015; Otto and Sicinski, 2017). Further links with the role of hypothermia in slowing down the events involved in the cell cycle and regulation of the apoptosis response, is indicated by inhibition of S-phase-associated kinase protein-2 (Skp2) which plays a major role in mediating the ubiquitination and subsequent proteasome degradation of p27(kip1), which is known to be involved in regulation of the G1/S transition during the cell cycle; and it also plays a role in neurogenesis, signal transduction and transcription (Podmirseg et al., 2016). It has been reported that Skp2 is up-regulated after TBI and this is linked to the inhibition of p27(kip1) expression and astrocyte proliferation (Liu et al., 2010). Thus, the hypothermic effect might be linked to astrocyte fate and survival following reduced blood and oxygen in TBI and cerebral edema.

It is established that hypothermia has both positive and negative effects upon many transcriptional regulation and adaptive responses. One of the possible negative effects suggested by the data here is the inhibition of some protective factors that attenuate the oxidative stress-mediated DNA damage such as the inhibitory effect on cyclic AMP-dependent transcription factor (ATF-2), a transcriptional activator and regulator for a large number of genes such as those involved in anti-apoptosis and DNA-damage and cell proliferation responses through its binding to CREB (cAMP response element). ATF-2 has also been shown to be inhibited in response to hypothermic intervention (Walluscheck et al., 2013). The downstream signaling of ATF is mediated through its binding to c-Jun or CREB (Harrison et al., 2004) and that it has been shown that its activation is increased via phosphorylation by p38 MAPK and JNK protein kinases (Pearson et al., 2001; Gadea et al., 2008).

At the protein level, the array results indicated that hypothermia significantly downregulated 18 proteins that have mixed pro- and anti-apoptotic effect (Table 3). The evidence that hypothermia caused a decrease in apoptosis agrees with various previous studies (Lee et al., 2012; Yenari and Han, 2012). Although hypothermia seems to have a global inhibitory effect, there was an interesting upregulation of only one protein during this acute phase, survivin. Survivin is an anti-apoptotic protein that depresses apoptosis via decreasing caspase 3 expression (Li et al., 2011). Survivin has been associated with the extent of vascularization within the infarct region (Conway et al., 2003). This increase in survivin expression could be part of an endogenous response mediated by hypothermia on astrocytes following hypoxia. These results are in agreement with the findings from Mormile et al. (2012) and suggests that part of the therapeutic benefit of hypothermia following hypoxia could be mediated by modulating survivin expression. Further studies are needed to understand the basic mechanisms associated with survivin regulation and function in astrocyte treated with hypothermia.

These results indicate that hypothermia could interfere with the main early events of apoptosis through its inhibitory effect on p53, HIF-1α and CREB and by enhancing the astrocytic survival in response to oxidative-stress mediated damage via interfering with the cell cycle regulatory processes and/or other essential elements, including survivin. Despite its beneficial effects during the acute phase of cytotoxic edema, hypothermia might also lead to a general slowing down effect that may include some essential cell survival events or enzymatic cascades (Boutilier, 2001; Goossens and Hachimi-Idrissi, 2014).

## Conclusion

This study provides the first detailed longitudinal investigation at the gene and protein expression levels of the molecular effects of therapeutic hypothermic intervention following hypoxia on primary cortical human astrocytes.

This study, for the first time, reports the involvement of Wnt pathway inhibition as the most significantly affected signaling cascade in the mechanism of hypothermia mediated response following hypoxia- and oxidative stress- activated insult on human astrocytes. Additionally, the results indicate that part of the protective effect of hypothermic intervention following hypoxia could be mediated through the inhibition of p38 and JNK signaling.

Global inhibition of Wnt signaling could be problematic due to its significant role in regulating essential processes involved in the functional regulation of stem and immune cells along with its effect on post-mitotic glial and neuronal cells (Chenn, 2008; Munji et al., 2011); this could be avoided through dissecting this generalized effect via specific targeting of signaling molecules.

Moreover, our results confirm the well-known effects of hypothermic intervention on attenuating the hypoxia activated HIF-1α- and p53-mediated apoptotic response. This hypothermic intervention will have a beneficial effect on the pro-inflammatory- and oxidative-stress-mediated elements but it could contribute to some negative outcomes through slowing down many essential molecules that are involved in the brain repair processes.

These results highlight the existence of different options for future therapies targeting cerebral diseases where injury- and oxidative-stress mediated inflammation drives secondary neurodegeneration, such as stroke and/or TBI.

The effects of hypothermia on astrocytes are complex and there is no single factor that could explain its neuroprotective effect, although it is possible that several of these effects may share a common upstream activator. Understanding the intracellular signaling networks underlying the beneficial effects of hypothermia is essential for discovery and validation of new potential therapeutic targets identified in this study using a global transcriptomic approach.

## Data accessibility

All relevant data are within the paper and its supporting Information files were made publicly available at: doi: 10.6084/m9.figshare.5285242.

## Author contributions

MS: performed all laboratory work, and initial data analysis, contributed to study design and helped draft the manuscript; MC, AC, RB, NW, PH, and PK: conceived the study, participated in its design and coordination, assisted in data and statistical analysis, and co-wrote the manuscript. All authors read and approved the final manuscript.

### Conflict of interest statement

The authors declare that the research was conducted in the absence of any commercial or financial relationships that could be construed as a potential conflict of interest.
