# Supplementary material for: Transcriptome Analysis of Gene Expression Provides New Insights into the Effect of Mild Therapeutic Hypothermia on Primary Human Cortical Astrocytes Cultured under Hypoxia
Source: Front Cell Neurosci. 2017 Dec 14;11:386. doi: 10.3389/fncel.2017.00386 (PMC5735114; doi:10.3389/fncel.2017.00386)
Supplement: Supplementary file 1 [file DataSheet1.docx]

**Supplementary table 1: Functional annotation clustering and gene ontology (GO) analysis of the significantly expressed genes (DEGs) following microarray analysis on primary human cortical astrocytes cultured under hypoxia alone or with hypothermic intervention.**

| **Condition** | **Up-regulated GO** | **E. score** | **Count** | ***p* value** | **Down-regulated GO** | **E. score** | **Count** | ***p* value** |
| --- | --- | --- | --- | --- | --- | --- | --- | --- |
| **Hypoxia+hypothermia vs Hypoxia** | 1. Cytoskeleton | 2.56 | 18 | 3.60E-02 | 1.     Wnt receptor signalling pathway, calcium modulating pathway | 1.49 | 3 | 9.60E-03 |
|  | 2. Chromosome | 2.05 | 13 | 2.20E-04 | 2.   Cellular calcium ion and cation homeostasis | 1.34 | 7 | 9.40E-03 |
|  | 3. ATP Binding | 1.76 | 25 | 4.00E-03 |  |  |  |  |
|  | 4. Phosphorus metabolic process | 1.76 | 14 | 9.50E-02 |  |  |  |  |
|  | 5. DNA metabolic process | 1.58 | 11 | 1.50E-02 |  |  |  |  |

**Supplementary table 2: Gene table for RT^2^ Profiler^TM^ PCR Array Human WNT Signalling Pathway Plus.**

| **Unigene** | **RefSeq number** | **Symbol** | **Description** |
| --- | --- | --- | --- |
| Hs.158932 | NM_000038 | APC | Adenomatous polyposis coli |
| Hs.592082 | NM_003502 | AXIN1 | Axin 1 |
| Hs.156527 | NM_004655 | AXIN2 | Axin 2 |
| Hs.643802 | NM_033637 | BTRC | Beta-transducin repeat containing |
| Hs.529862 | NM_001892 | CSNK1A1 | Casein kinase 1, alpha 1 |
| Hs.208597 | NM_001328 | CTBP1 | C-terminal binding protein 1 |
| Hs.712929 | NM_001904 | CTNNB1 | Catenin (cadherin-associated protein), beta 1, 88kDa |
| Hs.685322 | NM_020248 | CTNNBIP1 | Catenin, beta interacting protein 1 |
| Hs.654934 | NM_014992 | DAAM1 | Dishevelled associated activator of morphogenesis 1 |
| Hs.696631 | NM_001343 | DAB2 | Disabled homolog 2, mitogen-responsive phosphoprotein (Drosophila) |
| Hs.40499 | NM_012242 | DKK1 | Dickkopf homolog 1 (Xenopus laevis) |
| Hs.292156 | NM_015881 | DKK3 | Dickkopf homolog 3 (Xenopus laevis) |
| Hs.731450 | NM_004421 | DVL1 | Dishevelled, dsh homolog 1 (Drosophila) |
| Hs.118640 | NM_004422 | DVL2 | Dishevelled, dsh homolog 2 (Drosophila) |
| Hs.517517 | NM_001429 | EP300 | E1A binding protein p300 |
| Hs.484138 | NM_012300 | FBXW11 | F-box and WD repeat domain containing 11 |
| Hs.1755 | NM_002007 | FGF4 | Fibroblast growth factor 4 |
| Hs.283565 | NM_005438 | FOSL1 | FOS-like antigen 1 |
| Hs.126057 | NM_005479 | FRAT1 | Frequently rearranged in advanced T-cell lymphomas |
| Hs.128453 | NM_001463 | FRZB | Frizzled-related protein |
| Hs.94234 | NM_003505 | FZD1 | Frizzled family receptor 1 |
| Hs.142912 | NM_001466 | FZD2 | Frizzled family receptor 2 |
| Hs.40735 | NM_017412 | FZD3 | Frizzled family receptor 3 |
| Hs.591968 | NM_012193 | FZD4 | Frizzled family receptor 4 |
| Hs.17631 | NM_003468 | FZD5 | Frizzled family receptor 5 |
| Hs.591863 | NM_003506 | FZD6 | Frizzled family receptor 6 |
| Hs.173859 | NM_003507 | FZD7 | Frizzled family receptor 7 |
| Hs.302634 | NM_031866 | FZD8 | Frizzled family receptor 8 |
| Hs.647029 | NM_003508 | FZD9 | Frizzled family receptor 9 |
| Hs.445733 | NM_002093 | GSK3B | Glycogen synthase kinase 3 beta |
| Hs.696684 | NM_002228 | JUN | Jun proto-oncogene |
| Hs.229335 | NM_001039570 | KREMEN1 | Kringle containing transmembrane protein 1 |
| Hs.6347 | NM_002335 | LRP5 | Low density lipoprotein receptor-related protein 5 |
| Hs.584775 | NM_002336 | LRP6 | Low density lipoprotein receptor-related protein 6 |
| Hs.138211 | NM_002750 | MAPK8 | Mitogen-activated protein kinase 8 |
| Hs.2256 | NM_002423 | MMP7 | Matrix metallopeptidase 7 (matrilysin, uterine) |
| Hs.534074 | NM_172390 | NFATC1 | Nuclear factor of activated T-cells, cytoplasmic, calcineurin-dependent 1 |
| Hs.187578 | NM_033119 | NKD1 | Naked cuticle homolog 1 (Drosophila) |
| Hs.208759 | NM_016231 | NLK | Nemo-like kinase |
| Hs.643588 | NM_000325 | PITX2 | Paired-like homeodomain 2 |
| Hs.386453 | NM_022825 | PORCN | Porcupine homolog (Drosophila) |
| Hs.696032 | NM_006238 | PPARD | Peroxisome proliferator-activated receptor delta |
| Hs.524348 | NM_153026 | PRICKLE1 | Prickle homolog 1 (Drosophila) |
| Hs.247077 | NM_001664 | RHOA | Ras homolog gene family, member A |
| Hs.710868 | NM_003707 | RUVBL1 | RuvB-like 1 (E. coli) |
| Hs.213424 | NM_003012 | SFRP1 | Secreted frizzled-related protein 1 |
| Hs.658169 | NM_003014 | SFRP4 | Secreted frizzled-related protein 4 |
| Hs.98367 | NM_022454 | SOX17 | SRY (sex determining region Y)-box 17 |
| Hs.573153 | NM_003202 | TCF7 | Transcription factor 7 (T-cell specific, HMG-box) |
| Hs.516297 | NM_031283 | TCF7L1 | Transcription factor 7-like 1 (T-cell specific, HMG-box) |
| Hs.99477 | NM_020335 | VANGL2 | Vang-like 2 (van gogh, Drosophila) |
| Hs.284122 | NM_007191 | WIF1 | WNT inhibitory factor 1 |
| Hs.492974 | NM_003882 | WISP1 | WNT1 inducible signaling pathway protein 1 |
| Hs.248164 | NM_005430 | WNT1 | Wingless-type MMTV integration site family, member 1 |
| Hs.121540 | NM_025216 | WNT10A | Wingless-type MMTV integration site family, member 10A |
| Hs.108219 | NM_004626 | WNT11 | Wingless-type MMTV integration site family, member 11 |
| Hs.567356 | NM_003391 | WNT2 | Wingless-type MMTV integration site family member 2 |
| Hs.258575 | NM_004185 | WNT2B | Wingless-type MMTV integration site family, member 2B |
| Hs.445884 | NM_030753 | WNT3 | Wingless-type MMTV integration site family, member 3 |
| Hs.336930 | NM_033131 | WNT3A | Wingless-type MMTV integration site family, member 3A |
| Hs.25766 | NM_030761 | WNT4 | Wingless-type MMTV integration site family, member 4 |
| Hs.643085 | NM_003392 | WNT5A | Wingless-type MMTV integration site family, member 5A |
| Hs.306051 | NM_032642 | WNT5B | Wingless-type MMTV integration site family, member 5B |
| Hs.29764 | NM_006522 | WNT6 | Wingless-type MMTV integration site family, member 6 |
| Hs.72290 | NM_004625 | WNT7A | Wingless-type MMTV integration site family, member 7A |
| Hs.512714 | NM_058238 | WNT7B | Wingless-type MMTV integration site family, member 7B |
| Hs.591274 | NM_058244 | WNT8A | Wingless-type MMTV integration site family, member 8A |
| Hs.149504 | NM_003395 | WNT9A | Wingless-type MMTV integration site family, member 9A |
| Hs.425091 | NM_138369 | BOD1 | Biorientation of chromosomes in cell division 1 |
| Hs.282410 | NM_006888 | CALM1 | Calmodulin 1 (phosphorylase kinase, delta) |
| Hs.523852 | NM_053056 | CCND1 | Cyclin D1 |
| Hs.376071 | NM_001759 | CCND2 | Cyclin D2 |
| Hs.110488 | NM_014918 | CHSY1 | Chondroitin sulfate synthase 1 |
| Hs.627078 | NM_001338 | CXADR | Coxsackie virus and adenovirus receptor |
| Hs.587231 | NM_207352 | CYP4V2 | Cytochrome P450, family 4, subfamily v, polypeptide 2 |
| Hs.648448 | NM_025015 | HSPA12A | Heat shock 70kDa protein 12A |
| Hs.743478 | NM_016269 | LEF1 | Lymphoid enhancer-binding factor 1 |
| Hs.655199 | NM_005946 | MT1A | Metallothionein 1A |
| Hs.713636 | NM_016498 | MTFP1 | Mitochondrial fission process 1 |
| Hs.700429 | NM_014751 | MTSS1 | Metastasis suppressor 1 |
| Hs.202453 | NM_002467 | MYC | V-myc myelocytomatosis viral oncogene homolog (avian) |
| Hs.502116 | NM_145117 | NAV2 | Neuron navigator 2 |
| Hs.26006 | NM_018137 | PRMT6 | Protein arginine methyltransferase 6 |
| Hs.23348 | NM_005983 | SKP2 | S-phase kinase-associated protein 2 (p45) |
| Hs.520640 | NM_001101 | ACTB | Actin, beta |
| Hs.534255 | NM_004048 | B2M | Beta-2-microglobulin |
| Hs.592355 | NM_002046 | GAPDH | Glyceraldehyde-3-phosphate dehydrogenase |
| Hs.412707 | NM_000194 | HPRT1 | Hypoxanthine phosphoribosyltransferase 1 |
| Hs.546285 | NM_001002 | RPLP0 | Ribosomal protein, large, P0 |

**Supplementary table 3: Gene table for RT^2^ Profiler^TM^ PCR Array Human MAPK Signalling Pathway.**

| **Unigene** | **RefSeq number** | **Symbol** | **Description** |
| --- | --- | --- | --- |
| Hs.446641 | NM_001654 | ARAF | V-raf murine sarcoma 3611 viral oncogene homolog |
| Hs.592510 | NM_001880 | ATF2 | Activating transcription factor 2 |
| Hs.550061 | NM_004333 | BRAF | V-raf murine sarcoma viral oncogene homolog B1 |
| Hs.417050 | NM_003914 | CCNA1 | Cyclin A1 |
| Hs.58974 | NM_001237 | CCNA2 | Cyclin A2 |
| Hs.23960 | NM_031966 | CCNB1 | Cyclin B1 |
| Hs.194698 | NM_004701 | CCNB2 | Cyclin B2 |
| Hs.523852 | NM_053056 | CCND1 | Cyclin D1 |
| Hs.376071 | NM_001759 | CCND2 | Cyclin D2 |
| Hs.534307 | NM_001760 | CCND3 | Cyclin D3 |
| Hs.244723 | NM_001238 | CCNE1 | Cyclin E1 |
| Hs.467637 | NM_001791 | CDC42 | Cell division cycle 42 (GTP binding protein, 25kDa) |
| Hs.19192 | NM_001798 | CDK2 | Cyclin-dependent kinase 2 |
| Hs.95577 | NM_000075 | CDK4 | Cyclin-dependent kinase 4 |
| Hs.119882 | NM_001259 | CDK6 | Cyclin-dependent kinase 6 |
| Hs.370771 | NM_000389 | CDKN1A | Cyclin-dependent kinase inhibitor 1A (p21, Cip1) |
| Hs.238990 | NM_004064 | CDKN1B | Cyclin-dependent kinase inhibitor 1B (p27, Kip1) |
| Hs.106070 | NM_000076 | CDKN1C | Cyclin-dependent kinase inhibitor 1C (p57, Kip2) |
| Hs.512599 | NM_000077 | CDKN2A | Cyclin-dependent kinase inhibitor 2A (melanoma, p16, inhibits CDK4) |
| Hs.72901 | NM_004936 | CDKN2B | Cyclin-dependent kinase inhibitor 2B (p15, inhibits CDK4) |
| Hs.525324 | NM_078626 | CDKN2C | Cyclin-dependent kinase inhibitor 2C (p18, inhibits CDK4) |
| Hs.435051 | NM_001800 | CDKN2D | Cyclin-dependent kinase inhibitor 2D (p19, inhibits CDK4) |
| Hs.198998 | NM_001278 | CHUK | Conserved helix-loop-helix ubiquitous kinase |
| Hs.172928 | NM_000088 | COL1A1 | Collagen, type I, alpha 1 |
| Hs.516646 | NM_004379 | CREB1 | CAMP responsive element binding protein 1 |
| Hs.459759 | NM_004380 | CREBBP | CREB binding protein |
| Hs.533717 | NM_003836 | DLK1 | Delta-like 1 homolog (Drosophila) |
| Hs.654393 | NM_005225 | E2F1 | E2F transcription factor 1 |
| Hs.488293 | NM_005228 | EGFR | Epidermal growth factor receptor |
| Hs.326035 | NM_001964 | EGR1 | Early growth response 1 |
| Hs.181128 | NM_005229 | ELK1 | ELK1, member of ETS oncogene family |
| Hs.369438 | NM_005238 | ETS1 | V-ets erythroblastosis virus E26 oncogene homolog 1 (avian) |
| Hs.644231 | NM_005239 | ETS2 | V-Ets erythroblastosis virus E26 oncogene homolog 2 (avian) |
| Hs.25647 | NM_005252 | FOS | FBJ murine osteosarcoma viral oncogene homolog |
| Hs.444356 | NM_002086 | GRB2 | Growth factor receptor-bound protein 2 |
| Hs.37003 | NM_005343 | HRAS | V-Ha-ras Harvey rat sarcoma viral oncogene homolog |
| Hs.743241 | NM_005347 | HSPA5 | Heat shock 70kDa protein 5 (glucose-regulated protein, 78kDa) |
| Hs.520973 | NM_001540 | HSPB1 | Heat shock 27kDa protein 1 |
| Hs.696684 | NM_002228 | JUN | Jun proto-oncogene |
| Hs.505033 | NM_004985 | KRAS | V-Ki-ras2 Kirsten rat sarcoma viral oncogene homolog |
| Hs.133534 | NM_014238 | KSR1 | Kinase suppressor of ras 1 |
| Hs.433332 | NM_021970 | LAMTOR3 | Late endosomal/lysosomal adaptor, MAPK and MTOR activator 3 |
| Hs.145442 | NM_002755 | MAP2K1 | Mitogen-activated protein kinase kinase 1 |
| Hs.465627 | NM_030662 | MAP2K2 | Mitogen-activated protein kinase kinase 2 |
| Hs.514012 | NM_002756 | MAP2K3 | Mitogen-activated protein kinase kinase 3 |
| Hs.514681 | NM_003010 | MAP2K4 | Mitogen-activated protein kinase kinase 4 |
| Hs.114198 | NM_002757 | MAP2K5 | Mitogen-activated protein kinase kinase 5 |
| Hs.463978 | NM_002758 | MAP2K6 | Mitogen-activated protein kinase kinase 6 |
| Hs.531754 | NM_145185 | MAP2K7 | Mitogen-activated protein kinase kinase 7 |
| Hs.653654 | NM_005921 | MAP3K1 | Mitogen-activated protein kinase kinase kinase 1 |
| Hs.145605 | NM_006609 | MAP3K2 | Mitogen-activated protein kinase kinase kinase 2 |
| Hs.29282 | NM_002401 | MAP3K3 | Mitogen-activated protein kinase kinase kinase 3 |
| Hs.390428 | NM_005922 | MAP3K4 | Mitogen-activated protein kinase kinase kinase 4 |
| Hs.95424 | NM_007181 | MAP4K1 | Mitogen-activated protein kinase kinase kinase kinase 1 |
| Hs.431850 | NM_002745 | MAPK1 | Mitogen-activated protein kinase 1 |
| Hs.125503 | NM_002753 | MAPK10 | Mitogen-activated protein kinase 10 |
| Hs.57732 | NM_002751 | MAPK11 | Mitogen-activated protein kinase 11 |
| Hs.432642 | NM_002969 | MAPK12 | Mitogen-activated protein kinase 12 |
| Hs.178695 | NM_002754 | MAPK13 | Mitogen-activated protein kinase 13 |
| Hs.485233 | NM_001315 | MAPK14 | Mitogen-activated protein kinase 14 |
| Hs.861 | NM_002746 | MAPK3 | Mitogen-activated protein kinase 3 |
| Hs.411847 | NM_002748 | MAPK6 | Mitogen-activated protein kinase 6 |
| Hs.150136 | NM_002749 | MAPK7 | Mitogen-activated protein kinase 7 |
| Hs.138211 | NM_002750 | MAPK8 | Mitogen-activated protein kinase 8 |
| Hs.558180 | NM_012324 | MAPK8IP2 | Mitogen-activated protein kinase 8 interacting protein 2 |
| Hs.484371 | NM_002752 | MAPK9 | Mitogen-activated protein kinase 9 |
| Hs.643566 | NM_004759 | MAPKAPK2 | Mitogen-activated protein kinase-activated protein kinase 2 |
| Hs.234521 | NM_004635 | MAPKAPK3 | Mitogen-activated protein kinase-activated protein kinase 3 |
| Hs.285354 | NM_002382 | MAX | MYC associated factor X |
| Hs.649965 | NM_002397 | MEF2C | Myocyte enhancer factor 2C |
| Hs.371594 | NM_003684 | MKNK1 | MAP kinase interacting serine/threonine kinase 1 |
| Hs.533432 | NM_005372 | MOS | V-mos Moloney murine sarcoma viral oncogene homolog |
| Hs.655432 | NM_020998 | MST1 | Macrophage stimulating 1 (hepatocyte growth factor-like) |
| Hs.202453 | NM_002467 | MYC | V-myc myelocytomatosis viral oncogene homolog (avian) |
| Hs.77810 | NM_004554 | NFATC4 | Nuclear factor of activated T-cells, cytoplasmic, calcineurin-dependent 4 |
| Hs.486502 | NM_002524 | NRAS | Neuroblastoma RAS viral (v-ras) oncogene homolog |
| Hs.435714 | NM_002576 | PAK1 | P21 protein (Cdc42/Rac)-activated kinase 1 |
| Hs.120 | NM_004905 | PRDX6 | Peroxiredoxin 6 |
| Hs.413812 | NM_006908 | RAC1 | Ras-related C3 botulinum toxin substrate 1 (rho family, small GTP binding protein Rac1) |
| Hs.159130 | NM_002880 | RAF1 | V-raf-1 murine leukaemia viral oncogene homolog 1 |
| Hs.408528 | NM_000321 | RB1 | Retinoblastoma 1 |
| Hs.523718 | NM_006142 | SFN | Stratifin |
| Hs.75862 | NM_005359 | SMAD4 | SMAD family member 4 |
| Hs.437460 | NM_000546 | TP53 | Tumor protein p53 |
| Hs.520640 | NM_001101 | ACTB | Actin, beta |
| Hs.534255 | NM_004048 | B2M | Beta-2-microglobulin |
| Hs.592355 | NM_002046 | GAPDH | Glyceraldehyde-3-phosphate dehydrogenase |
| Hs.412707 | NM_000194 | HPRT1 | Hypoxanthine phosphoribosyltransferase 1 |
| Hs.546285 | NM_001002 | RPLP0 | Ribosomal protein, large, P0 |

**Supplementary table 4: Analytes represented in the human apoptosis array Kit (ARY009) and their corresponding western blotting antibodies used in western blotting (**[**www.rndsystems.com)**](http://www.rndsystems.com))**.**

| Analyte | Phospho-Specific Antibodies | Affinity Purified Polyclonal Antibodies (Total) | Monoclonal Antibodies (Total) | Labeled Antibodies (Total) |
| --- | --- | --- | --- | --- |
| Bad |  | [AF819](https://www.rndsystems.com/products/human-mouse-bad-aa-1-21-antibody_af819) | [MAB6405](https://www.rndsystems.com/products/human-bad-antibody-612312_mab6405) |  |
| Bax |  | [AF820](https://www.rndsystems.com/products/human-mouse-bax-antibody_af820) | [MAB846](https://www.rndsystems.com/products/human-bax-minus-c-terminus-antibody-127606_mab846), [MAB883](https://www.rndsystems.com/search?keywords=mab883), [2282-MC-100](https://www.rndsystems.com/products/human-bax-aa-3-16-antibody-yth-2d2_2282-mc) |  |
| Bcl-2 |  | [AF810](https://www.rndsystems.com/products/human-mouse-bcl-2-antibody_af810) | [MAB8272](https://www.rndsystems.com/products/human-mouse-rat-bcl-2-antibody-625509_mab8272) |  |
| Bcl-x |  | [AF800](https://www.rndsystems.com/products/human-mouse-bcl-x-antibody_af800) |  |  |
| Cleaved Caspase-3 |  |  | [MAB835](https://www.rndsystems.com/products/human-mouse-cleaved-caspase-3-asp175-antibody-269518_mab835) |  |
| Catalase |  | [AF3398](https://www.rndsystems.com/products/human-mouse-rat-catalase-antibody_af3398) |  |  |
| cIAP-1 |  | [AF8181](https://www.rndsystems.com/products/human-ciap-1-hiap-2-antibody_af8181) | [MAB818](https://www.rndsystems.com/products/human-ciap-1-hiap-2-antibody-681732_mab818) |  |
| cIAP-2 |  | [AF8171](https://www.rndsystems.com/products/human-ciap-2-hiap-1-antibody_af8171) | [MAB817](https://www.rndsystems.com/products/human-mouse-ciap-2-hiap-1-antibody-315304_mab817), [MAB3400](https://www.rndsystems.com/products/human-mouse-ciap-pan-specific-antibody-315301_mab3400), [MAB3310](https://www.rndsystems.com/products/human-claspin-antibody-485508_mab3310) | [BAF8171](https://www.rndsystems.com/products/human-ciap-2-hiap-1-biotinylated-antibody_baf8171) |
| Claspin |  | [AF3310](https://www.rndsystems.com/products/0) |  | [BAF3310](https://www.rndsystems.com/products/human-claspin-biotinylated-antibody_baf3310) |
| Clusterin |  | [AF2937](https://www.rndsystems.com/products/human-clusterin-isoform-1-antibody_af2937) | [MAB2937](https://www.rndsystems.com/products/human-clusterin-antibody-350227_mab2937) | [BAF2937](https://www.rndsystems.com/products/human-clusterin-biotinylated-antibody_baf2937) |
| Cytochrome C |  |  | [MAB897](https://www.rndsystems.com/products/human-mouse-rat-cytochrome-c-antibody-7h82c12_mab897) |  |
| Trail R1/DR4 |  | [AF347](https://www.rndsystems.com/products/human-trail-r1-tnfrsf10a-antibody_af347) |  | [BAF347](https://www.rndsystems.com/products/human-trail-r1-tnfrsf10a-biotinylated-antibody_baf347) |
| Trail R2/DR5 |  | [AF631](https://www.rndsystems.com/products/human-trail-r2-tnfrsf10b-antibody_af631) |  | [BAF631](https://www.rndsystems.com/products/human-trail-r2-tnfrsf10b-biotinylated-antibody_baf631) |
| FADD | [MAB7047](https://www.rndsystems.com/products/human-phospho-fadd-s194-antibody-709736_mab7047) (S194) | [AF2938](https://www.rndsystems.com/products/human-fadd-antibody_af2938) |  |  |
| Fas/TNFSF6 |  | [AF326](https://www.rndsystems.com/products/human-fas-tnfrsf6-cd95-antibody_af326) | [MAB142](https://www.rndsystems.com/products/human-fas-tnfrsf6-cd95-antibody-dx2_mab142) | [BAF326](https://www.rndsystems.com/products/human-fas-tnfrsf6-cd95-biotinylated-antibody_baf326) |
| HIF-1alpha |  | [AF1935](https://www.rndsystems.com/products/human-mouse-rat-hif-1alpha-antibody_af1935) | [MAB1536](https://www.rndsystems.com/search?keywords=mab1536) |  |
| HO-1/HMOX1/ HSP32 |  | [AF3776](https://www.rndsystems.com/products/human-mouse-rat-hif-1alpha-antibody-241809_mab1536) | [MAB3776](https://www.rndsystems.com/products/human-mouse-ho-1-hmox1-hsp32-antibody-412811_mab3776) |  |
| HO-2/HMOX2 |  | [AF3170](https://www.rndsystems.com/products/human-mouse-rat-ho-2-hmox2-antibody_af3170) | [MAB3170](https://www.rndsystems.com/products/human-mouse-ho-2-hmox2-antibody-322913_mab3170) |  |
| HSP27 | [AF2314](https://www.rndsystems.com/products/human-mouse-rat-phospho-hsp27-s78-s82-antibody_af2314) (S78/S82) | [AF1580](https://www.rndsystems.com/products/human-mouse-hsp27-antibody_af1580), [AF15801](https://www.rndsystems.com/products/human-mouse-rat-hsp27-antibody_af15801) |  |  |
| HSP60 |  | [AF1800](https://www.rndsystems.com/products/human-mouse-rat-hsp60-antibody_af1800) | [MAB1800](https://www.rndsystems.com/products/mouse-fgf-r5-fgfrl1-antibody-223003_mab1899) |  |
| HSP70 |  | [AF1663](https://www.rndsystems.com/products/human-mouse-rat-hsp70-hspa1a-antibody_af1663) | [MAB1663](https://www.rndsystems.com/products/human-mouse-rat-hsp70-hspa1a-antibody-242707_mab1663) |  |
| HTRA2/Omi |  | [AF1458](https://www.rndsystems.com/products/human-mouse-rat-htra2-omi-antibody_af1458) | [MAB1458](https://www.rndsystems.com/products/human-htra2-omi-antibody-229926_mab1458) |  |
| Livin |  | [AF1161](https://www.rndsystems.com/products/human-livin-antibody_af1161) |  |  |
| PON2 |  | [AF4344](https://www.rndsystems.com/products/human-pon2-antibody_af4344) | [MAB4344](https://www.rndsystems.com/products/human-pon2-antibody-453709_mab4344) |  |
| p21/CIP1/CDNK1A |  | [AF1047](https://www.rndsystems.com/products/human-p21-cip1-cdkn1a-antibody_af1047) | [MAB1047](https://www.rndsystems.com/products/human-p21-cip1-cdkn1a-antibody-195720_mab1047) | [BAF1047](https://www.rndsystems.com/products/human-p21-cip1-cdkn1a-biotinylated-antibody_baf1047) |
| p27/Kip1 | [AF1555](https://www.rndsystems.com/products/human-phospho-p27-kip1-t157-antibody_af1555) (T157), [AF3994](https://www.rndsystems.com/products/human-phospho-p27-kip1-t198-antibody_af3994) (T198) | [AF2256](https://www.rndsystems.com/products/human-mouse-rat-p27-kip1-antibody_af2256) | [MAB2256](https://www.rndsystems.com/products/human-mouse-rat-p27-kip1-antibody-225501_mab2256) |  |
| phospho-p53 (S15) | [AF1043](https://www.rndsystems.com/products/human-phospho-p53-s15-antibody_af1043) (S15), [MAB1839](https://www.rndsystems.com/products/human-phospho-p53-s15-antibody-261352_mab1839) (S15) | [AF1355](https://www.rndsystems.com/products/human-mouse-rat-p53-antibody_af1355) | [MAB1355](https://www.rndsystems.com/products/human-mouse-rat-p53-antibody-184721_mab1355) | [BAF1355](https://www.rndsystems.com/products/human-mouse-rat-p53-biotinylated-antibody_baf1355), [HAF1355](https://www.rndsystems.com/products/human-mouse-rat-p53-horseradish-peroxidase-conjugated-antibody_haf1355) |
| phospho-p53 (S46) | [AF1489](https://www.rndsystems.com/products/human-phospho-p53-s46-antibody_af1489) (S46), [MAB1489](https://www.rndsystems.com/products/human-phospho-p53-s46-antibody-1022g_mab1489) (S46) | [AF1355](https://www.rndsystems.com/products/human-mouse-rat-p53-antibody_af1355) | [MAB1355](https://www.rndsystems.com/products/human-mouse-rat-p53-antibody-184721_mab1355) | [BAF1355](https://www.rndsystems.com/products/human-mouse-rat-p53-biotinylated-antibody_baf1355), [HAF1355](https://www.rndsystems.com/search?keywords=haf1355) |
| phospho-p53 (S392) | [AF2996](https://www.rndsystems.com/products/human-phospho-p53-s392-antibody_af2996) (S392) | [AF1355](https://www.rndsystems.com/products/human-mouse-rat-p53-antibody_af1355) | [MAB1355](https://www.rndsystems.com/products/human-mouse-rat-p53-antibody-184721_mab1355) | [BAF1355](https://www.rndsystems.com/products/human-mouse-rat-p53-biotinylated-antibody_baf1355), [HAF1355](https://www.rndsystems.com/search?keywords=haf1355) |
| phospho-Rad17 (S635) | [AF1374](https://www.rndsystems.com/products/human-phospho-rad17-s635-antibody_af1374) (S635) | [AF1926](https://www.rndsystems.com/products/human-rat-rad17-antibody_af1926) |  |  |
| SMAC/Diablo |  | [AF789](https://www.rndsystems.com/products/human-smac-diablo-antibody_af789), [AF7891](https://www.rndsystems.com/products/human-smac-diablo-antibody_af7891) |  | [BAF789](https://www.rndsystems.com/products/human-smac-diablo-biotinylated-antibody_baf789) |
| TNF RI/TNFRSF1A |  | [AF225](https://www.rndsystems.com/products/human-tnf-ri-tnfrsf1a-antibody_af225) | [MAB225](https://www.rndsystems.com/products/human-tnf-ri-tnfrsf1a-antibody-16803_mab225), [MAB625](https://www.rndsystems.com/products/human-tnf-ri-tnfrsf1a-antibody-16805_mab625) | [BAF225](https://www.rndsystems.com/products/human-tnf-ri-tnfrsf1a-biotinylated-antibody_baf225) |
| XIAP |  | [AF8221](https://www.rndsystems.com/products/human-mouse-rat-xiap-antibody_af8221) | [MAB822](https://www.rndsystems.com/products/human-mouse-xiap-antibody-117320_mab822) | [BAF8221](https://www.rndsystems.com/products/human-mouse-xiap-biotinylated-antibody_baf8221) |

**Supplementary table 5: Differentially expressed (A) up-regulated and (B) down-regulated genes following exposure to hypothermia on astrocytes cultured under hypoxia**

**(A)**

| **Probe Name** | **p value** | **FC (abs)** | **Regulation** | **Gene Symbol** | **Description** | **UniGene ID** |
| --- | --- | --- | --- | --- | --- | --- |
| A_33_P3251871 | 0.020578803 | 11.210708 | up | XLOC_l2_015206 | BROAD Institute lincRNA (XLOC_l2_015206), lincRNA [TCONS_l2_00029332] | |
| A_22_P00019604 | 0.043512538 | 9.496125 | up | lnc-KIF16B-1 | LNCipedia lincRNA (lnc-KIF16B-1), lincRNA [lnc-KIF16B-1:1] |  |
| A_33_P3345549 | 0.01953522 | 8.536874 | up | L2HGDH | Homo sapiens L-2-hydroxyglutarate dehydrogenase (L2HGDH), mRNA [NM_024884] | Hs.256034 |
| A_23_P500433 | 0.005350987 | 5.3837843 | up | CARD9 | Homo sapiens caspase recruitment domain family, member 9 (CARD9), transcript variant 1, mRNA [NM_052813] | Hs.694071 |
| A_21_P0005971 | 0.02082249 | 5.28588 | up | lnc-RBM12B-2 | LNCipedia lincRNA (lnc-RBM12B-2), lincRNA [lnc-RBM12B-2:1] |  |
| A_23_P127676 | 0.0437514 | 5.1272283 | up | CTR9 | Homo sapiens CTR9, Paf1/RNA polymerase II complex component (CTR9), mRNA [NM_014633] | Hs.725151 |
| A_23_P397543 | 0.03433153 | 4.87322 | up | LINC00174 | Homo sapiens long intergenic non-protein coding RNA 174 (LINC00174), long non-coding RNA [NR_026873] | Hs.50755 |
| A_33_P3420757 | 0.000317383 | 4.609771271 | up | AQP4 | Homo sapiens aquaporin 4 (AQP4), transcript variant a, mRNA [NM_001650] | Hs.315369 |
| A_23_P24004 | 0.03169583 | 4.600304 | up | IFIT2 | Homo sapiens interferon-induced protein with tetratricopeptide repeats 2 (IFIT2), mRNA [NM_001547] | Hs.437609 |
| A_23_P252193 | 0.03772137 | 4.4667726 | up | ITGA9 | Homo sapiens integrin, alpha 9 (ITGA9), mRNA [NM_002207] | Hs.113157 |
| A_19_P00321332 | 0.018326053 | 4.405929 | up | NEAT1 | Homo sapiens nuclear paraspeckle assembly transcript 1 (non-protein coding) (NEAT1), long non-coding RNA [NR_028272] | Hs.523789 |
| A_33_P3221568 | 0.026593119 | 4.29723 | up | ARMC5 | Homo sapiens armadillo repeat containing 5 (ARMC5), transcript variant 2, mRNA [NM_024742] | Hs.732945 |
| A_21_P0013854 | 0.01231584 | 4.2742724 | up |  |  |  |
| A_24_P185394 | 0.043023955 | 4.172266 | up | GSK3A | Homo sapiens glycogen synthase kinase 3 alpha (GSK3A), mRNA [NM_019884] | Hs.466828 |
| A_22_P00014212 | 0.026239166 | 4.170293 | up | LOC101928323 | Homo sapiens uncharacterized LOC101928323 (LOC101928323), long non-coding RNA [NR_125400] | Hs.657338 |
| A_21_P0014422 | 0.028355854 | 3.9875958 | up | LOC646513 | PREDICTED: Homo sapiens VLGN1945 (LOC646513), misc_RNA [XR_159371] | Hs.565803 |
| A_23_P93690 | 0.008691865 | 3.9222374 | up | MCM7 | Homo sapiens minichromosome maintenance complex component 7 (MCM7), transcript variant 2, mRNA [NM_182776] | Hs.438720 |
| A_24_P547010 | 0.0493298 | 3.7916727 | up |  |  |  |
| A_24_P517252 | 0.02010545 | 3.7583733 | up | PRCD | Homo sapiens progressive rod-cone degeneration (PRCD), transcript variant 1, mRNA [NM_001077620] | Hs.634380 |
| A_19_P00322498 | 0.042211086 | 3.6895888 | up |  | PREDICTED: Homo sapiens uncharacterized LOC102723493 (LOC102723493), transcript variant X1, ncRNA [XR_424607] | |
| A_24_P338788 | 0.03875059 | 3.6636024 | up | CSNK1A1L | Homo sapiens casein kinase 1, alpha 1-like (CSNK1A1L), mRNA [NM_145203] | Hs.512897 |
| A_23_P162970 | 0.039646424 | 3.647159 | up | IPO4 | Homo sapiens importin 4 (IPO4), transcript variant 1, mRNA [NM_024658] | Hs.411865 |
| A_23_P23850 | 0.048957475 | 3.522157 | up | DAB1 | Homo sapiens Dab, reelin signal transducer, homolog 1 (Drosophila) (DAB1), mRNA [NM_021080] | Hs.477370 |
| A_23_P159125 | 0.023442902 | 3.488036 | up | SLC16A5 | Homo sapiens solute carrier family 16 (monocarboxylate transporter), member 5 (SLC16A5), transcript variant 1, mRNA [NM_004695] | Hs.592095 |
| A_33_P3303385 | 0.002589175 | 3.4543345 | up | NCAPD2 | Homo sapiens non-SMC condensin I complex, subunit D2 (NCAPD2), mRNA [NM_014865] | Hs.5719 |
| A_23_P8311 | 0.04392303 | 3.4531748 | up | TDP2 | Homo sapiens tyrosyl-DNA phosphodiesterase 2 (TDP2), mRNA [NM_016614] | Hs.403010 |
| A_23_P157449 | 0.040710848 | 3.428383 | up | POLR2K | Homo sapiens polymerase (RNA) II (DNA directed) polypeptide K, 7.0kDa (POLR2K), mRNA [NM_005034] | Hs.351475 |
| A_23_P377616 | 0.032060467 | 3.4277236 | up | CIRBP | cold inducible RNA binding protein [Source:HGNC Symbol;Acc:HGNC:1982] [ENST00000621399] | Hs.618145 |
| A_22_P00020557 | 0.021268046 | 3.395222 | up | lnc-AC010536.1-1 | Q9SCR0_ARATH (Q9SCR0) Scarecrow-like 7 (SCL7), partial (4%) [THC2724708] | |
| A_23_P66180 | 0.030328887 | 3.3448896 | up | CACNG3 | Homo sapiens calcium channel, voltage-dependent, gamma subunit 3 (CACNG3), mRNA [NM_006539] | Hs.7235 |
| A_33_P3314471 | 0.007968558 | 3.3042865 | up | IMMT | inner membrane protein, mitochondrial [Source:HGNC Symbol;Acc:HGNC:6047] [ENST00000474969] | |
| A_23_P55731 | 0.03833126 | 3.2864985 | up | CIC | Homo sapiens capicua transcriptional repressor (CIC), mRNA [NM_015125] | Hs.388236 |
| A_23_P94591 | 0.04560761 | 3.2630134 | up | TMEM141 | Homo sapiens transmembrane protein 141 (TMEM141), mRNA [NM_032928] | Hs.356744 |
| A_23_P60599 | 0.019731002 | 3.1811035 | up | UGT1A6 | Homo sapiens UDP glucuronosyltransferase 1 family, polypeptide A6 (UGT1A6), transcript variant 1, mRNA [NM_001072] | Hs.554822 |
| A_32_P70135 | 0.035501264 | 3.1473875 | up | PROSER1 | Homo sapiens proline and serine rich 1 (PROSER1), mRNA [NM_025138] | Hs.318526 |
| A_33_P3369567 | 0.02287892 | 3.1198552 | up | LSP1 | Homo sapiens lymphocyte-specific protein 1 (LSP1), transcript variant 6, mRNA [NM_001289005] | Hs.56729 |
| A_33_P3390778 | 0.048831757 | 3.103801 | up | TRIM46 | Homo sapiens tripartite motif containing 46 (TRIM46), transcript variant 2, mRNA [NM_001256599] | Hs.287735 |
| A_22_P00010533 | 0.025638778 | 3.1013522 | up | lnc-NCF4-1 | LNCipedia lincRNA (lnc-NCF4-1), lincRNA [lnc-NCF4-1:1] |  |
| A_24_P189739 | 0.012731256 | 3.0869586 | up | DUSP16 | Homo sapiens dual specificity phosphatase 16 (DUSP16), mRNA [NM_030640] | Hs.536535 |
| A_24_P941787 | 0.029709453 | 3.0228345 | up | PRPF4B | Homo sapiens pre-mRNA processing factor 4B (PRPF4B), mRNA [NM_003913] | Hs.159014 |
| A_22_P00024064 | 0.043813307 | 3.0149698 | up | OGFR-AS1 | Homo sapiens OGFR antisense RNA 1 (OGFR-AS1), long non-coding RNA [NR_102430] | |
| A_22_P00011020 | 0.042247336 | 2.9936752 | up | lnc-NT5C-2 | LNCipedia lincRNA (lnc-NT5C-2), lincRNA [lnc-NT5C-2:1] |  |
| A_22_P00022215 | 0.03130548 | 2.9772341 | up |  |  |  |
| A_33_P3211569 | 0.02827686 | 2.953068 | up | ERBB3 | Homo sapiens v-erb-b2 avian erythroblastic leukemia viral oncogene homolog 3 (ERBB3), transcript variant s, mRNA [NM_001005915] | Hs.118681 |
| A_21_P0007948 | 0.043510955 | 2.950191 | up | lnc-AL359392.1-2 | LNCipedia lincRNA (lnc-AL359392.1-2), lincRNA [lnc-AL359392.1-2:1] | |
| A_33_P3351101 | 0.011837876 | 2.9165833 | up | TYSND1 | Homo sapiens trypsin domain containing 1 (TYSND1), transcript variant 1, mRNA [NM_173555] | Hs.533655 |
| A_33_P3248580 | 0.006095667 | 2.9150631 | up |  | chromobox homolog 3 pseudogene 3 [Source:HGNC Symbol;Acc:HGNC:42875] [ENST00000508108] | |
| A_22_P00009847 | 0.03674845 | 2.8881962 | up | BDNF-AS | Homo sapiens BDNF antisense RNA (BDNF-AS), transcript variant BT1C, long non-coding RNA [NR_033312] | Hs.675323 |
| A_33_P3390918 | 0.028763989 | 2.8860226 | up | FSD2 | Homo sapiens fibronectin type III and SPRY domain containing 2 (FSD2), transcript variant 1, mRNA [NM_001007122] | Hs.719372 |
| A_21_P0004202 | 0.005147204 | 2.882694 | up | lnc-C5orf38-1 | LNCipedia lincRNA (lnc-C5orf38-1), lincRNA [lnc-C5orf38-1:3] |  |
| A_33_P3248629 | 0.04600485 | 2.8809912 | up | DENND2A | DENN/MADD domain containing 2A [Source:HGNC Symbol;Acc:HGNC:22212] [ENST00000492720] | Hs.6385 |
| A_33_P3389837 | 0.028502578 | 2.8713639 | up | GPN3 | Homo sapiens GPN-loop GTPase 3 (GPN3), transcript variant 2, mRNA [NM_001164372] | Hs.634680 |
| A_22_P00004246 | 0.015488482 | 2.8334053 | up |  |  |  |
| A_23_P26905 | 0.019855795 | 2.8253467 | up | POLG2 | Homo sapiens polymerase (DNA directed), gamma 2, accessory subunit (POLG2), mRNA [NM_007215] | Hs.437009 |
| A_23_P155848 | 3.51E-04 | 2.823677 | up | DKK2 | Homo sapiens dickkopf WNT signaling pathway inhibitor 2 (DKK2), mRNA [NM_014421] | Hs.211869 |
| A_22_P00019189 | 0.032911696 | 2.817968 | up | LINC01431 | Homo sapiens long intergenic non-protein coding RNA 1431 (LINC01431), long non-coding RNA [NR_109884] | Hs.140295 |
| A_22_P00018101 | 0.03209185 | 2.8044431 | up |  |  |  |
| A_23_P429092 | 0.046657644 | 2.7950172 | up | HBS1L | Homo sapiens HBS1-like translational GTPase (HBS1L), transcript variant 3, mRNA [NM_001145207] | Hs.378532 |
| A_33_P3374117 | 4.92E-04 | 2.7408493 | up | LOC101927507 | anoctamin 7 pseudogene 1 [Source:HGNC Symbol;Acc:HGNC:32248] [ENST00000602586] | |
| A_33_P3289466 | 0.031745218 | 2.6310666 | up |  |  |  |
| A_21_P0000289 | 0.03053641 | 2.6213818 | up | SNORD45A | Homo sapiens small nucleolar RNA, C/D box 45A (SNORD45A), small nucleolar RNA [NR_002749] | |
| A_22_P00014988 | 0.048890267 | 2.613341 | up | C3orf80 | Homo sapiens chromosome 3 open reading frame 80 (C3orf80), mRNA [NM_001168214] | Hs.729755 |
| A_33_P3233055 | 0.008296309 | 2.6064281 | up |  | UI-H-BW1-anr-f-12-0-UI.s1 NCI_CGAP_Sub7 Homo sapiens cDNA clone IMAGE:3083302 3', mRNA sequence [BF515046] | Hs.720773 |
| A_24_P42389 | 0.0436901 | 2.5933807 | up | OTUD6A | OTU deubiquitinase 6A [Source:HGNC Symbol;Acc:HGNC:32312] [ENST00000338352] | Hs.447381 |
| A_21_P0014773 | 0.004984786 | 2.570306 | up |  | TRX2_MOUSE (O08550) Trithorax homolog 2 (WW domain-binding protein 7) (Fragment), partial (7%) [THC2668279] | |
| A_22_P00015648 | 0.02493574 | 2.5589886 | up | lnc-SUSD1-2 | LNCipedia lincRNA (lnc-SUSD1-2), lincRNA [lnc-SUSD1-2:1] |  |
| A_24_P942321 | 0.039878305 | 2.5522556 | up | FRMD4A | Homo sapiens FERM domain containing 4A (FRMD4A), mRNA [NM_018027] | Hs.330463 |
| A_21_P0007184 | 0.029380787 | 2.5471509 | up | lnc-NAV2-1 | LNCipedia lincRNA (lnc-NAV2-1), lincRNA [lnc-NAV2-1:1] |  |
| A_33_P3244834 | 0.031807788 | 2.5407782 | up |  |  |  |
| A_33_P3330972 | 0.022086482 | 2.530948 | up |  |  |  |
| A_23_P73801 | 0.029315012 | 2.524136 | up | TCEAL1 | Homo sapiens transcription elongation factor A (SII)-like 1 (TCEAL1), transcript variant 3, mRNA [NM_001006640] | Hs.95243 |
| A_24_P865672 | 0.002156077 | 2.523689 | up |  | nucleophosmin 1 (nucleolar phosphoprotein B23, numatrin) pseudogene 42 [Source:HGNC Symbol;Acc:HGNC:45221] [ENST00000560329] | |
| A_33_P3377459 | 0.022826986 | 2.5142343 | up | PAPD5 | Homo sapiens PAP associated domain containing 5 (PAPD5), transcript variant 1, mRNA [NM_001040284] | Hs.514342 |
| A_23_P211909 | 0.008760396 | 2.513033 | up | PLS1 | Homo sapiens plastin 1 (PLS1), transcript variant 2, mRNA [NM_002670] | Hs.203637 |
| A_23_P59099 | 0.005619492 | 2.510823 | up | OR11A1 | Homo sapiens olfactory receptor, family 11, subfamily A, member 1 (OR11A1), mRNA [NM_013937] | Hs.676010 |
| A_24_P171983 | 0.038470913 | 2.5067616 | up | NDUFAF4 | Homo sapiens NADH dehydrogenase (ubiquinone) complex I, assembly factor 4 (NDUFAF4), mRNA [NM_014165] | Hs.512144 |
| A_19_P00812554 | 0.010623072 | 2.5050077 | up | USP2-AS1 | Homo sapiens USP2 antisense RNA 1 (head to head) (USP2-AS1), long non-coding RNA [NR_034160] | Hs.662365 |
| A_33_P3354823 | 0.04867136 | 2.4830737 | up | BTBD19 | Homo sapiens BTB (POZ) domain containing 19 (BTBD19), mRNA [NM_001136537] | Hs.632400 |
| A_32_P43812 | 0.04378506 | 2.4724822 | up | DCUN1D4 | Homo sapiens DCN1, defective in cullin neddylation 1, domain containing 4 (DCUN1D4), transcript variant 1, mRNA [NM_001040402] | Hs.605388 |
| A_21_P0001704 | 0.005492356 | 2.4569156 | up | lnc-NBPF16-4 | LNCipedia lincRNA (lnc-NBPF16-4), lincRNA [lnc-NBPF16-4:2] | Hs.654925 |
| A_33_P3722568 | 0.019024191 | 2.4558265 | up | LINC00685 | Homo sapiens long intergenic non-protein coding RNA 685 (LINC00685), transcript variant 1, long non-coding RNA [NR_027232] | Hs.575741 |
| A_22_P00022314 | 0.042114858 | 2.4366024 | up | MSH6 | mutS homolog 6 [Source:HGNC Symbol;Acc:HGNC:7329] [ENST00000454137] | |
| A_23_P162589 | 0.035088025 | 2.4326293 | up | VDR | Homo sapiens vitamin D (1,25- dihydroxyvitamin D3) receptor (VDR), transcript variant 2, mRNA [NM_001017535] | Hs.524368 |
| A_33_P3373163 | 0.047813658 | 2.431735 | up |  | ATPase, H+ transporting, lysosomal 16kDa, V0 subunit c pseudogene 1 [Source:HGNC Symbol;Acc:HGNC:31323] [ENST00000482495] | |
| A_19_P00316396 | 0.00286739 | 2.4125268 | up | MEG3 | Homo sapiens maternally expressed 3 (non-protein coding) (MEG3), transcript variant 16, long non-coding RNA [NR_046473] | Hs.654863 |
| A_21_P0008982 | 0.031720422 | 2.4031904 | up |  |  |  |
| A_21_P0006901 | 0.039347067 | 2.3903465 | up | lnc-CNNM1-2 | LNCipedia lincRNA (lnc-CNNM1-2), lincRNA [lnc-CNNM1-2:1] |  |
| A_32_P32905 | 0.046911784 | 2.383791 | up | MEAF6 | Homo sapiens MYST/Esa1-associated factor 6 (MEAF6), transcript variant 1, mRNA [NM_022756] | Hs.17118 |
| A_22_P00025715 | 0.04123515 | 2.37731 | up | lnc-PDZK1IP1-1 | LNCipedia lincRNA (lnc-PDZK1IP1-1), lincRNA [lnc-PDZK1IP1-1:1] |  |
| A_22_P00014084 | 0.022033954 | 2.3672292 | up | HAND2-AS1 | Homo sapiens HAND2 antisense RNA 1 (head to head) (HAND2-AS1), long non-coding RNA [NR_003679] | Hs.61435 |
| A_21_P0002732 | 0.04565755 | 2.3638074 | up | LOC102725370 | DA372386 BRTHA2 Homo sapiens cDNA clone BRTHA2002622 5', mRNA sequence [DA372386] | Hs.628261 |
| A_33_P3228023 | 0.0445158 | 2.3571475 | up | SCPEP1 | Homo sapiens serine carboxypeptidase 1 (SCPEP1), mRNA [NM_021626] | Hs.514950 |
| A_21_P0000076 | 0.012612317 | 2.3564837 | up | CDK1 | Homo sapiens cyclin-dependent kinase 1 (CDK1), transcript variant 4, mRNA [NM_001170406] | Hs.732435 |
| A_22_P00015061 | 0.017763173 | 2.350419 | up | lnc-SNRPD1-3 | LNCipedia lincRNA (lnc-SNRPD1-3), lincRNA [lnc-SNRPD1-3:1] |  |
| A_33_P3227284 | 0.048161805 | 2.348531 | up | OGT | Homo sapiens O-linked N-acetylglucosamine (GlcNAc) transferase (OGT), transcript variant 1, mRNA [NM_181672] | Hs.405410 |
| A_21_P0010080 | 0.042001195 | 2.3455086 | up | lnc-C20orf187-1 | DB030984 TESTI2 Homo sapiens cDNA clone TESTI2014797 5', mRNA sequence [DB030984] | Hs.570364 |
| A_33_P3833256 | 0.049568284 | 2.3440485 | up | LOC440028 | Homo sapiens uncharacterized LOC440028 (LOC440028), long non-coding RNA [NR_033972] | Hs.677541 |
| A_24_P225616 | 0.003808152 | 2.3295543 | up | RRM2 | Homo sapiens ribonucleotide reductase M2 (RRM2), transcript variant 2, mRNA [NM_001034] | Hs.226390 |
| A_24_P107317 | 0.018526835 | 2.310274 | up | USP2 | Homo sapiens ubiquitin specific peptidase 2 (USP2), transcript variant 1, mRNA [NM_004205] | Hs.524085 |
| A_24_P253003 | 0.033388946 | 2.2893956 | up | WNT11 | Homo sapiens wingless-type MMTV integration site family, member 11 (WNT11), mRNA [NM_004626] | Hs.108219 |
| A_23_P389371 | 0.011320714 | 2.2687643 | up | MRGPRX3 | Homo sapiens MAS-related GPR, member X3 (MRGPRX3), mRNA [NM_054031] | Hs.380177 |
| A_23_P58489 | 0.043541454 | 2.2624555 | up | FASTKD3 | Homo sapiens FAST kinase domains 3 (FASTKD3), transcript variant 1, mRNA [NM_024091] | Hs.653162 |
| A_24_P198629 | 0.020159354 | 2.2611618 | up | LINS | Homo sapiens lines homolog (Drosophila) (LINS), mRNA [NM_001040616] | Hs.105633 |
| A_21_P0000304 | 0.010834448 | 2.2506728 | up | SNORA13 | Homo sapiens small nucleolar RNA, H/ACA box 13 (SNORA13), small nucleolar RNA [NR_002922] | Hs.689709 |
| A_23_P34744 | 0.037231743 | 2.250242 | up | CTSK | Homo sapiens cathepsin K (CTSK), mRNA [NM_000396] | Hs.632466 |
| A_21_P0011533 | 0.048099175 | 2.230035 | up |  |  | Hs.745165 |
| A_23_P310086 | 0.04083183 | 2.2283351 | up | BEND6 | Homo sapiens BEN domain containing 6 (BEND6), mRNA [NM_152731] | Hs.582993 |
| A_21_P0009365 | 0.047356628 | 2.2256784 | up | LOC101928447 | PREDICTED: Homo sapiens uncharacterized LOC101928447 (LOC101928447), ncRNA [XR_243759] | |
| A_33_P3225507 | 0.040414352 | 2.2226074 | up | OR10G2 | Homo sapiens olfactory receptor, family 10, subfamily G, member 2 (OR10G2), mRNA [NM_001005466] | Hs.554580 |
| A_23_P76622 | 0.047958273 | 2.218863 | up | DCT | Homo sapiens dopachrome tautomerase (DCT), transcript variant 1, mRNA [NM_001922] | Hs.301865 |
| A_21_P0003368 | 0.03545234 | 2.2184846 | up |  | DA725066 NT2RI3 Homo sapiens cDNA clone NT2RI3006635 5', mRNA sequence [DA725066] | Hs.743919 |
| A_22_P00024264 | 0.037469383 | 2.2165904 | up | lnc-ADD1-1 | Homo sapiens mRNA, exon 1, 2, 3, 4, clone:RES4-24B. [AB000465] | Hs.398178 |
| A_21_P0009873 | 0.04617356 | 2.213899 | up | LOC100505771 | PREDICTED: Homo sapiens uncharacterized LOC100505771 (LOC100505771), transcript variant 2, ncRNA [XR_109592] | Hs.448392 |
| A_33_P3323048 | 0.04826772 | 2.2129807 | up | SLC10A7 | Homo sapiens solute carrier family 10, member 7 (SLC10A7), transcript variant 2, mRNA [NM_001029998] | Hs.659209 |
| A_21_P0010301 | 0.014237576 | 2.212308 | up | lnc-TSPEAR-1 | LNCipedia lincRNA (lnc-TSPEAR-1), lincRNA [lnc-TSPEAR-1:2] |  |
| A_23_P40805 | 0.003164703 | 2.2117467 | up | KLF15 | Homo sapiens Kruppel-like factor 15 (KLF15), mRNA [NM_014079] | Hs.272215 |
| A_23_P395595 | 0.016029539 | 2.2105126 | up | FNBP4 | Homo sapiens formin binding protein 4 (FNBP4), mRNA [NM_015308] | Hs.6834 |
| A_24_P397386 | 0.034832425 | 2.2104068 | up | LIFR | Homo sapiens leukemia inhibitory factor receptor alpha (LIFR), transcript variant 2, mRNA [NM_002310] | Hs.133421 |
| A_21_P0014331 | 0.027396563 | 2.1875408 | up | LOC100505942 | Homo sapiens uncharacterized LOC100505942 (LOC100505942), long non-coding RNA [NR_104656] | Hs.121233 |
| A_33_P3311755 | 0.049343802 | 2.1864748 | up | KIF23 | Homo sapiens kinesin family member 23 (KIF23), transcript variant 1, mRNA [NM_138555] | Hs.270845 |
| A_21_P0000467 | 0.047419105 | 2.1746829 | up |  |  |  |
| A_22_P00005824 | 0.041254647 | 2.1695848 | up | lnc-ERAL1-1 | DKFZp781J1536_s1 781 (synonym: hlcc4) Homo sapiens cDNA clone DKFZp781J1536 3', mRNA sequence [BX644926] | Hs.729146 |
| A_21_P0011619 | 0.002931151 | 2.168256 | up | TBC1D3P5 | Homo sapiens TBC1 domain family, member 3 pseudogene 5 (TBC1D3P5), non-coding RNA [NR_033892] | Hs.385755 |
| A_23_P99625 | 0.028413871 | 2.1534338 | up | FITM1 | Homo sapiens fat storage-inducing transmembrane protein 1 (FITM1), mRNA [NM_203402] | Hs.128060 |
| A_23_P160240 | 0.036714267 | 2.1522377 | up | ACP6 | Homo sapiens acid phosphatase 6, lysophosphatidic (ACP6), mRNA [NM_016361] | Hs.562154 |
| A_22_P00015513 | 0.047279526 | 2.1515496 | up | LOC101929294 | Homo sapiens uncharacterized LOC101929294 (LOC101929294), long non-coding RNA [NR_125808] | Hs.637673 |
| A_21_P0013763 | 0.046358425 | 2.146643 | up | XLOC_l2_015520 | BROAD Institute lincRNA (XLOC_l2_015520), lincRNA [TCONS_l2_00030204] | |
| A_33_P3380597 | 0.005206732 | 2.136589 | up | PRR14L | Homo sapiens proline rich 14-like (PRR14L), mRNA [NM_173566] | Hs.438906 |
| A_21_P0007567 | 0.028173393 | 2.1348314 | up | lnc-XRCC6BP1-1 | LNCipedia lincRNA (lnc-XRCC6BP1-1), lincRNA [lnc-XRCC6BP1-1:1] |  |
| A_23_P165007 | 0.036155365 | 2.1331158 | up | RASGRP4 | Homo sapiens RAS guanyl releasing protein 4 (RASGRP4), transcript variant a, mRNA [NM_170604] | Hs.130434 |
| A_23_P205046 | 0.03623986 | 2.1238563 | up | ANKRD10 | Homo sapiens ankyrin repeat domain 10 (ANKRD10), transcript variant 1, mRNA [NM_017664] | Hs.525163 |
| A_33_P3336562 | 0.010667448 | 2.122475 | up |  |  |  |
| A_33_P3367550 | 0.010289396 | 2.1204295 | up | LOC401242 | Homo sapiens uncharacterized LOC401242 (LOC401242), long non-coding RNA [NR_033379] | Hs.448285 |
| A_21_P0012487 | 0.027114654 | 2.1195183 | up | XLOC_l2_010511 | BROAD Institute lincRNA (XLOC_l2_010511), lincRNA [TCONS_l2_00019725] | Hs.652562 |
| A_23_P250735 | 0.042067718 | 2.1195176 | up | CBX7 | Homo sapiens chromobox homolog 7 (CBX7), mRNA [NM_175709] | Hs.356416 |
| A_22_P00012669 | 0.034455024 | 2.1185772 | up | FAM184B | Homo sapiens family with sequence similarity 184, member B (FAM184B), mRNA [NM_015688] | Hs.744951 |
| A_33_P3375145 | 0.002480072 | 2.1079104 | up | LURAP1L | Homo sapiens leucine rich adaptor protein 1-like (LURAP1L), mRNA [NM_203403] | Hs.445356 |
| A_32_P11894 | 0.019296972 | 2.103357 | up | C12orf65 | Homo sapiens chromosome 12 open reading frame 65 (C12orf65), transcript variant 1, mRNA [NM_152269] | Hs.319128 |
| A_21_P0000341 | 0.029228944 | 2.1004255 | up | SNORA61 | Homo sapiens small nucleolar RNA, H/ACA box 61 (SNORA61), small nucleolar RNA [NR_002987] | Hs.632377 |
| A_23_P349406 | 0.03514992 | 2.0995677 | up | RIMKLA | Homo sapiens ribosomal modification protein rimK-like family member A (RIMKLA), mRNA [NM_173642] | Hs.420244 |
| A_21_P0013535 | 7.38E-04 | 2.0964806 | up | XLOC_l2_014123 | BROAD Institute lincRNA (XLOC_l2_014123), lincRNA [TCONS_l2_00028472] | Hs.546711 |
| A_23_P61674 | 0.011238277 | 2.093897 | up | CLK4 | Homo sapiens CDC-like kinase 4 (CLK4), mRNA [NM_020666] | Hs.406557 |
| A_22_P00008242 | 0.005144687 | 2.0828195 | up |  |  | Hs.439634 |
| A_23_P305938 | 0.042483836 | 2.063574 | up | ZNF75D | Homo sapiens zinc finger protein 75D (ZNF75D), transcript variant 1, mRNA [NM_007131] | Hs.533540 |
| A_33_P3291569 | 0.037008714 | 2.0612624 | up | LUC7L3 | Homo sapiens LUC7-like 3 (S. cerevisiae) (LUC7L3), transcript variant 1, mRNA [NM_016424] | Hs.130293 |
| A_22_P00016387 | 0.035011597 | 2.0595822 | up |  |  |  |
| A_22_P00002844 | 0.022952966 | 2.0479028 | up | lnc-C3orf30-2 | LNCipedia lincRNA (lnc-C3orf30-2), lincRNA [lnc-C3orf30-2:2] |  |
| A_23_P423926 | 0.006039781 | 2.0477877 | up | SS18L1 | Homo sapiens synovial sarcoma translocation gene on chromosome 18-like 1 (SS18L1), transcript variant 1, mRNA [NM_198935] | Hs.154429 |
| A_33_P3391275 | 0.043060713 | 2.0433297 | up | LINC00494 | Homo sapiens long intergenic non-protein coding RNA 494 (LINC00494), long non-coding RNA [NR_026958] | Hs.299080 |
| A_33_P3314550 | 0.029389113 | 2.035932 | up | RAB3A | Homo sapiens RAB3A, member RAS oncogene family (RAB3A), mRNA [NM_002866] | Hs.27744 |
| A_23_P56654 | 0.00843715 | 2.0310588 | up | MCEE | Homo sapiens methylmalonyl CoA epimerase (MCEE), mRNA [NM_032601] | Hs.94949 |
| A_21_P0005612 | 0.002979127 | 2.0306406 | up | lnc-AC099552.4.1-2 | LNCipedia lincRNA (lnc-AC099552.4.1-2), lincRNA [lnc-AC099552.4.1-2:3] | |
| A_19_P00802201 | 0.049963772 | 2.0268593 | up |  | CCNB2 pseudogene 1 [Source:HGNC Symbol;Acc:HGNC:50850] [ENST00000399411] | |
| A_21_P0001346 | 2.09E-04 | 2.0241394 | up | lnc-TMEM56-1 | LNCipedia lincRNA (lnc-TMEM56-1), lincRNA [lnc-TMEM56-1:1] |  |
| A_23_P120243 | 0.01480521 | 2.0204449 | up | HOXD1 | Homo sapiens homeobox D1 (HOXD1), mRNA [NM_024501] | Hs.83465 |
| A_33_P3310039 | 0.008570859 | 2.0140026 | up | TVP23A | Homo sapiens trans-golgi network vesicle protein 23 homolog A (S. cerevisiae) (TVP23A), mRNA [NM_001079512] | Hs.371576 |
| A_23_P395404 | 0.002320626 | 2.0108328 | up | FGF20 | Homo sapiens fibroblast growth factor 20 (FGF20), mRNA [NM_019851] | Hs.199905 |
| A_22_P00022398 | 0.027329303 | 2.008533 | up | AGBL5-AS1 | Homo sapiens AGBL5 antisense RNA 1 (AGBL5-AS1), long non-coding RNA [NR_046730] | |
| A_24_P248863 | 0.009654455 | 2.0032625 | up | ZCCHC4 | Homo sapiens zinc finger, CCHC domain containing 4 (ZCCHC4), mRNA [NM_024936] | Hs.278945 |
| A_22_P00011121 | 0.021436006 | 2.0031395 | up | LOC101928143 | PREDICTED: Homo sapiens uncharacterized LOC101928143 (LOC101928143), transcript variant X1, ncRNA [XR_245781] | Hs.737221 |
| A_33_P3303562 | 0.04425114 | 1.9977105 | up |  |  |  |
| A_23_P150950 | 0.040632688 | 1.9942144 | up | ZFC3H1 | Homo sapiens zinc finger, C3H1-type containing (ZFC3H1), mRNA [NM_144982] | Hs.527874 |
| A_22_P00012901 | 0.03453507 | 1.9897327 | up | HAUS5 | Homo sapiens HAUS augmin-like complex, subunit 5 (HAUS5), mRNA [NM_015302] | Hs.7426 |
| A_33_P3218768 | 0.038861632 | 1.9891462 | up | UPF2 | Homo sapiens UPF2 regulator of nonsense transcripts homolog (yeast) (UPF2), transcript variant 1, mRNA [NM_080599] | Hs.370689 |
| A_23_P387523 | 0.043516725 | 1.986725 | up | ZBTB40 | Homo sapiens zinc finger and BTB domain containing 40 (ZBTB40), transcript variant 1, mRNA [NM_001083621] | Hs.418966 |
| A_33_P3378101 | 0.008599056 | 1.9864391 | up | AGBL4 | Homo sapiens ATP/GTP binding protein-like 4 (AGBL4), mRNA [NM_032785] | Hs.679809 |
| A_24_P159837 | 0.042013768 | 1.986055 | up | ZNF302 | Homo sapiens zinc finger protein 302 (ZNF302), transcript variant 15, mRNA [NM_018675] | Hs.436350 |
| A_23_P202269 | 3.02E-04 | 1.9841028 | up | ANK3 | Homo sapiens ankyrin 3, node of Ranvier (ankyrin G) (ANK3), transcript variant 1, mRNA [NM_020987] | Hs.499725 |
| A_21_P0012985 | 0.007665583 | 1.9809005 | up | lnc-FGF10-3 | LNCipedia lincRNA (lnc-FGF10-3), lincRNA [lnc-FGF10-3:6] | Hs.744696 |
| A_33_P3304538 | 0.025600508 | 1.9800899 | up | RNF207 | Homo sapiens ring finger protein 207 (RNF207), mRNA [NM_207396] | Hs.716549 |
| A_24_P265856 | 0.046331096 | 1.968261 | up | SENP7 | Homo sapiens SUMO1/sentrin specific peptidase 7 (SENP7), transcript variant 1, mRNA [NM_020654] | Hs.529551 |
| A_23_P111724 | 0.035403218 | 1.9667058 | up | RUNDC3B | Homo sapiens RUN domain containing 3B (RUNDC3B), transcript variant 1, mRNA [NM_138290] | Hs.411488 |
| A_23_P70359 | 6.51E-04 | 1.96313 | up | AGPAT4-IT1 | Homo sapiens AGPAT4 intronic transcript 1 (non-protein coding) (AGPAT4-IT1), long non-coding RNA [NR_024277] | Hs.664873 |
| A_22_P00000868 | 0.04792113 | 1.9620743 | up | LOC100505942 | Homo sapiens uncharacterized LOC100505942 (LOC100505942), long non-coding RNA [NR_104656] | Hs.121233 |
| A_23_P399251 | 0.03596695 | 1.9612905 | up | NBPF22P | Homo sapiens neuroblastoma breakpoint family, member 22, pseudogene (NBPF22P), non-coding RNA [NR_003719] | Hs.449272 |
| A_22_P00005088 | 0.007853844 | 1.9605253 | up | lnc-DGAT2-1 | LNCipedia lincRNA (lnc-DGAT2-1), lincRNA [lnc-DGAT2-1:1] |  |
| A_23_P89981 | 0.048102833 | 1.9550726 | up | CYP2F1 | Homo sapiens cytochrome P450, family 2, subfamily F, polypeptide 1 (CYP2F1), mRNA [NM_000774] | Hs.558318 |
| A_33_P3323939 | 0.035504058 | 1.9482163 | up | LIPN | Homo sapiens lipase, family member N (LIPN), mRNA [NM_001102469] | Hs.632091 |
| A_22_P00004821 | 0.04155299 | 1.9386709 | up | SLC25A5-AS1 | Homo sapiens SLC25A5 antisense RNA 1 (SLC25A5-AS1), long non-coding RNA [NR_028443] | Hs.714405 |
| A_23_P372368 | 0.029026626 | 1.9341731 | up |  | Homo sapiens C21orf87 protein (C21orf87) mRNA, complete cds. [AF426265] | Hs.729612 |
| A_33_P3212027 | 0.029077977 | 1.928771 | up | ELF2 | Homo sapiens E74-like factor 2 (ets domain transcription factor) (ELF2), transcript variant 1, mRNA [NM_201999] | Hs.634040 |
| A_33_P3231140 | 0.007439354 | 1.9253037 | up | ANKRD50 | Homo sapiens ankyrin repeat domain 50 (ANKRD50), transcript variant 2, mRNA [NM_001167882] | Hs.480694 |
| A_23_P133133 | 0.008667295 | 1.9186181 | up | ALPK1 | Homo sapiens alpha-kinase 1 (ALPK1), transcript variant 1, mRNA [NM_025144] | Hs.652825 |
| A_24_P67027 | 0.047375523 | 1.9155054 | up | CLK2 | Homo sapiens CDC-like kinase 2 (CLK2), transcript variant 1, mRNA [NM_001294338] | Hs.73986 |
| A_23_P112086 | 0.010615105 | 1.9147344 | up | DEFA5 | Homo sapiens defensin, alpha 5, Paneth cell-specific (DEFA5), mRNA [NM_021010] | Hs.655233 |
| A_33_P3406706 | 0.048145775 | 1.9144813 | up | EXOG | Homo sapiens endo/exonuclease (5'-3'), endonuclease G-like (EXOG), transcript variant 1, mRNA [NM_005107] | Hs.517897 |
| A_33_P3716128 | 0.042208448 | 1.9113415 | up | SMC4 | Homo sapiens structural maintenance of chromosomes 4 (SMC4), transcript variant 1, mRNA [NM_005496] | Hs.58992 |
| A_22_P00003884 | 0.008190648 | 1.908815 | up | LINC00475 | Homo sapiens long intergenic non-protein coding RNA 475 (LINC00475), long non-coding RNA [NR_027341] | Hs.149940 |
| A_23_P131383 | 0.03368357 | 1.9029025 | up | FANCL | Homo sapiens Fanconi anemia, complementation group L (FANCL), transcript variant 2, mRNA [NM_018062] | Hs.631890 |
| A_22_P00000733 | 0.024952194 | 1.8988787 | up | lnc-ADAMTS9-1 | LNCipedia lincRNA (lnc-ADAMTS9-1), lincRNA [lnc-ADAMTS9-1:1] |  |
| A_23_P74309 | 0.016117098 | 1.8982673 | up | NOS1AP | Homo sapiens nitric oxide synthase 1 (neuronal) adaptor protein (NOS1AP), transcript variant 1, mRNA [NM_014697] | Hs.731942 |
| A_33_P3225487 | 0.008699405 | 1.8974373 | up | HSD17B7 | Homo sapiens hydroxysteroid (17-beta) dehydrogenase 7 (HSD17B7), mRNA [NM_016371] | Hs.492925 |
| A_23_P151297 | 0.038665846 | 1.8923467 | up | TNS2 | Homo sapiens tensin 2 (TNS2), transcript variant 1, mRNA [NM_015319] | Hs.6147 |
| A_21_P0008099 | 0.026812611 | 1.8903245 | up | SLITRK5 | SLIT and NTRK-like family, member 5 [Source:HGNC Symbol;Acc:HGNC:20295] [ENST00000325089] | |
| A_23_P146497 | 0.017244723 | 1.8901365 | up | PPP1R26 | Homo sapiens protein phosphatase 1, regulatory subunit 26 (PPP1R26), mRNA [NM_014811] | Hs.533260 |
| A_24_P51683 | 0.032509714 | 1.8863264 | up | CDK5R2 | Homo sapiens cyclin-dependent kinase 5, regulatory subunit 2 (p39) (CDK5R2), mRNA [NM_003936] | Hs.158460 |
| A_23_P318464 | 0.018009616 | 1.8812252 | up | EPHA8 | Homo sapiens EPH receptor A8 (EPHA8), transcript variant 1, mRNA [NM_020526] | Hs.283613 |
| A_33_P3299634 | 0.0292539 | 1.8786774 | up | OR1L6 | olfactory receptor, family 1, subfamily L, member 6 [Source:HGNC Symbol;Acc:HGNC:8218] [ENST00000373684] | |
| A_21_P0000774 | 0.020618405 | 1.8716874 | up | STK4-AS1 | Homo sapiens STK4 antisense RNA 1 (head to head) (STK4-AS1), long non-coding RNA [NR_038341] | Hs.196065 |
| A_22_P00013046 | 5.88E-04 | 1.8680103 | up | RGS5 | regulator of G-protein signaling 5 [Source:HGNC Symbol;Acc:HGNC:10001] [ENST00000530241] | |
| A_23_P102471 | 0.017564584 | 1.8610775 | up | MSH2 | Homo sapiens mutS homolog 2 (MSH2), transcript variant 1, mRNA [NM_000251] | Hs.597656 |
| A_21_P0014745 | 0.04103841 | 1.859613 | up |  | Homo sapiens mRNA; cDNA DKFZp781J0350 (from clone DKFZp781J0350). [CR933683] | Hs.626983 |
| A_21_P0001446 | 0.033902694 | 1.8509995 | up | lnc-EFCAB2-1 | LNCipedia lincRNA (lnc-EFCAB2-1), lincRNA [lnc-EFCAB2-1:1] |  |
| A_23_P107173 | 0.033358034 | 1.8474123 | up | MEOX1 | Homo sapiens mesenchyme homeobox 1 (MEOX1), transcript variant 1, mRNA [NM_004527] | Hs.438 |
| A_21_P0012907 | 0.013208725 | 1.8468027 | up | XLOC_l2_012035 | BROAD Institute lincRNA (XLOC_l2_012035), lincRNA [TCONS_l2_00022897] | |
| A_21_P0007440 | 0.03692417 | 1.841313 | up | LOC102724209 | PREDICTED: Homo sapiens uncharacterized LOC102724209 (LOC102724209), ncRNA [XR_424249] | |
| A_23_P102832 | 0.032392364 | 1.8410525 | up | CEP250 | Homo sapiens centrosomal protein 250kDa (CEP250), mRNA [NM_007186] | Hs.443976 |
| A_23_P253524 | 0.041659318 | 1.8396034 | up | CENPE | Homo sapiens centromere protein E, 312kDa (CENPE), transcript variant 1, mRNA [NM_001813] | Hs.75573 |
| A_21_P0012284 | 0.043290146 | 1.8387269 | up |  | sphingomyelin phosphodiesterase 4, neutral membrane (neutral sphingomyelinase-3) pseudogene 1 [Source:HGNC Symbol;Acc:HGNC:39673] [ENST00000416717] | |
| A_22_P00011713 | 0.02506366 | 1.830826 | up | lnc-PDZD9-1 | LNCipedia lincRNA (lnc-PDZD9-1), lincRNA [lnc-PDZD9-1:2] |  |
| A_23_P354297 | 5.37E-04 | 1.8301914 | up | CHTF18 | Homo sapiens CTF18, chromosome transmission fidelity factor 18 homolog (S. cerevisiae) (CHTF18), mRNA [NM_022092] | Hs.153850 |
| A_33_P3311498 | 0.022305615 | 1.8215652 | up | TRHDE-AS1 | Homo sapiens TRHDE antisense RNA 1 (TRHDE-AS1), transcript variant 1, long non-coding RNA [NR_026837] | Hs.363603 |
| A_22_P00017888 | 0.004597951 | 1.8188488 | up | lnc-ZFAT-3 | LNCipedia lincRNA (lnc-ZFAT-3), lincRNA [lnc-ZFAT-3:1] |  |
| A_23_P161481 | 0.032190736 | 1.8137641 | up | PALD1 | Homo sapiens phosphatase domain containing, paladin 1 (PALD1), mRNA [NM_014431] | Hs.202351 |
| A_23_P144684 | 0.014143872 | 1.8135133 | up | ANKRD32 | Homo sapiens ankyrin repeat domain 32 (ANKRD32), mRNA [NM_032290] | Hs.657315 |
| A_22_P00019960 | 0.022766953 | 1.808977 | up | lnc-CALML6-1 | LNCipedia lincRNA (lnc-CALML6-1), lincRNA [lnc-CALML6-1:1] |  |
| A_23_P146783 | 0.0205763 | 1.8058981 | up | NEB | Homo sapiens nebulin (NEB), transcript variant 3, mRNA [NM_004543] | Hs.588655 |
| A_23_P338952 | 0.03988135 | 1.8057859 | up | S100PBP | Homo sapiens S100P binding protein (S100PBP), transcript variant 1, mRNA [NM_022753] | Hs.440880 |
| A_23_P115331 | 0.03730328 | 1.8053651 | up | AHDC1 | Homo sapiens AT hook, DNA binding motif, containing 1 (AHDC1), mRNA [NM_001029882] | Hs.469280 |
| A_22_P00002126 | 0.023870666 | 1.8050462 | up | DGUOK-AS1 | Homo sapiens DGUOK antisense RNA 1 (DGUOK-AS1), transcript variant 2, long non-coding RNA [NR_104030] | |
| A_22_P00024236 | 0.041551363 | 1.8018712 | up | lnc-MRPS5-2 | HSMALD {Homo sapiens} (exp=-1; wgp=0; cg=0), partial (58%) [THC2623746] | Hs.585935 |
| A_21_P0005484 | 0.038142774 | 1.8001401 | up | lnc-SKAP2-1 | LNCipedia lincRNA (lnc-SKAP2-1), lincRNA [lnc-SKAP2-1:2] |  |
| A_21_P0007168 | 0.02534693 | 1.8000027 | up | LOC101927503 | PREDICTED: Homo sapiens uncharacterized LOC101927503 (LOC101927503), transcript variant X2, misc_RNA [XR_428869] | Hs.436626 |
| A_21_P0008219 | 0.03215713 | 1.7917995 | up | lnc-TUBGCP3-6 | LNCipedia lincRNA (lnc-TUBGCP3-6), lincRNA [lnc-TUBGCP3-6:1] |  |
| A_24_P935491 | 0.009999297 | 1.7898757 | up | COL3A1 | Homo sapiens collagen, type III, alpha 1 (COL3A1), mRNA [NM_000090] | Hs.443625 |
| A_19_P00318878 | 0.008308979 | 1.7854108 | up |  | Homo sapiens cDNA clone IMAGE:4794941. [BC030094] | Hs.498418 |
| A_33_P3254606 | 0.02662694 | 1.7834668 | up | WDR62 | Homo sapiens WD repeat domain 62 (WDR62), transcript variant 1, mRNA [NM_001083961] | Hs.116244 |
| A_22_P00004038 | 0.040194925 | 1.7815309 | up | lnc-CHRNA3-1 | Homo sapiens cholinergic receptor, nicotinic, alpha 5, mRNA (cDNA clone IMAGE:6189913). [BC050469] | Hs.719767 |
| A_22_P00006414 | 0.021550752 | 1.7786397 | up | lnc-FBXO25-2 | LNCipedia lincRNA (lnc-FBXO25-2), lincRNA [lnc-FBXO25-2:1] |  |
| A_33_P3393135 | 0.027750077 | 1.7755382 | up | HIST1H2AI | Homo sapiens histone cluster 1, H2ai, mRNA (cDNA clone MGC:138459 IMAGE:8327722), complete cds. [BC112254] | Hs.534035 |
| A_21_P0004460 | 0.004207548 | 1.7726647 | up | lnc-EBF1-2 | LNCipedia lincRNA (lnc-EBF1-2), lincRNA [lnc-EBF1-2:1] |  |
| A_32_P129968 | 0.048028387 | 1.7706128 | up | ZNF284 | zinc finger protein 284 [Source:HGNC Symbol;Acc:HGNC:13078] [ENST00000421176] | Hs.445395 |
| A_23_P200030 | 0.025017226 | 1.7650301 | up | FPGT | Homo sapiens fucose-1-phosphate guanylyltransferase (FPGT), transcript variant 1, mRNA [NM_003838] | Hs.480085 |
| A_19_P00322354 | 0.04711223 | 1.7648263 | up | MAGI2-AS3 | Homo sapiens MAGI2 antisense RNA 3 (MAGI2-AS3), transcript variant 1, long non-coding RNA [NR_038343] | Hs.31474 |
| A_22_P00001044 | 0.002641659 | 1.7637638 | up | C1orf86 | chromosome 1 open reading frame 86 [Source:HGNC Symbol;Acc:HGNC:26428] [ENST00000497675] | Hs.107101 |
| A_33_P3304162 | 0.04885262 | 1.7634767 | up | COL19A1 | Homo sapiens collagen, type XIX, alpha 1 (COL19A1), mRNA [NM_001858] | Hs.444842 |
| A_33_P3252359 | 0.023408161 | 1.7616426 | up | BDH1 | Homo sapiens 3-hydroxybutyrate dehydrogenase, type 1 (BDH1), transcript variant 3, mRNA [NM_203314] | Hs.274539 |
| A_22_P00017937 | 0.04726691 | 1.7580938 | up | EIF2S3 | eukaryotic translation initiation factor 2, subunit 3 gamma, 52kDa [Source:HGNC Symbol;Acc:HGNC:3267] [ENST00000460032] | |
| A_33_P3339070 | 0.004393059 | 1.756557 | up | LINC00704 | Homo sapiens long intergenic non-protein coding RNA 704 (LINC00704), long non-coding RNA [NR_024475] | Hs.634869 |
| A_23_P321473 | 0.002589643 | 1.748591 | up | FAM76A | Homo sapiens family with sequence similarity 76, member A (FAM76A), transcript variant 3, mRNA [NM_152660] | Hs.469359 |
| A_33_P3261803 | 0.020276638 | 1.7454045 | up | FAAH | Homo sapiens fatty acid amide hydrolase (FAAH), mRNA [NM_001441] | Hs.720143 |
| A_24_P937855 | 0.04972168 | 1.7349725 | up | SIKE1 | Homo sapiens suppressor of IKBKE 1 (SIKE1), transcript variant 1, mRNA [NM_001102396] | Hs.709277 |
| A_33_P3358851 | 0.020668156 | 1.7291193 | up |  |  |  |
| A_22_P00017270 | 4.66E-05 | 1.7257373 | up |  |  |  |
| A_22_P00002572 | 0.0331273 | 1.7254043 | up | LOC100506388 | Homo sapiens uncharacterized LOC100506388 (LOC100506388), transcript variant 1, mRNA [NM_001242780] | Hs.627744 |
| A_33_P3214466 | 0.002709973 | 1.7233297 | up | MESP1 | Homo sapiens mesoderm posterior basic helix-loop-helix transcription factor 1 (MESP1), mRNA [NM_018670] | Hs.447531 |
| A_21_P0004383 | 0.041010085 | 1.7225295 | up | lnc-C5orf39-1 | LNCipedia lincRNA (lnc-C5orf39-1), lincRNA [lnc-C5orf39-1:5] |  |
| A_24_P387321 | 0.026172299 | 1.7223457 | up | ZNF44 | Homo sapiens zinc finger protein 44 (ZNF44), transcript variant 2, mRNA [NM_016264] | Hs.296731 |
| A_32_P790284 | 0.044991445 | 1.7185045 | up | KATNAL2 | Homo sapiens katanin p60 subunit A-like 2 (KATNAL2), mRNA [NM_031303] | Hs.404137 |
| A_33_P3227506 | 0.019758554 | 1.7159266 | up | BPTF | Homo sapiens bromodomain PHD finger transcription factor (BPTF), transcript variant 1, mRNA [NM_182641] | Hs.444200 |
| A_19_P00322762 | 0.029930549 | 1.7154906 | up | ZNF83 | zinc finger protein 83 [Source:HGNC Symbol;Acc:HGNC:13158] [ENST00000598190] | Hs.369632 |
| A_23_P43071 | 0.007283707 | 1.7153772 | up | MTERF3 | Homo sapiens mitochondrial transcription termination factor 3 (MTERF3), transcript variant 1, mRNA [NM_015942] | Hs.308613 |
| A_33_P3284453 | 0.04919756 | 1.7140799 | up | SOGA3 | Homo sapiens SOGA family member 3 (SOGA3), mRNA [NM_001012279] | Hs.319247 |
| A_23_P109895 | 0.03325479 | 1.7128985 | up | SLC26A6 | Homo sapiens solute carrier family 26 (anion exchanger), member 6 (SLC26A6), transcript variant 4, mRNA [NM_001040454] | Hs.631925 |
| A_23_P160025 | 0.006553302 | 1.7052833 | up | IFI16 | Homo sapiens interferon, gamma-inducible protein 16 (IFI16), transcript variant 2, mRNA [NM_005531] | Hs.380250 |
| A_33_P3382910 | 0.017284919 | 1.7038227 | up | GIN1 | Homo sapiens gypsy retrotransposon integrase 1 (GIN1), mRNA [NM_017676] | Hs.24088 |
| A_23_P258493 | 0.021709992 | 1.7021192 | up | LMNB1 | Homo sapiens lamin B1 (LMNB1), transcript variant 1, mRNA [NM_005573] | Hs.89497 |
| A_24_P413884 | 0.006685219 | 1.6994201 | up | CENPA | Homo sapiens centromere protein A (CENPA), transcript variant 1, mRNA [NM_001809] | Hs.1594 |
| A_33_P3276813 | 0.013248958 | 1.6961932 | up | EYS | Homo sapiens eyes shut homolog (Drosophila) (EYS), transcript variant 1, mRNA [NM_001142800] | Hs.25067 |
| A_23_P3177 | 0.036631912 | 1.6919638 | up | KCNK13 | Homo sapiens potassium channel, two pore domain subfamily K, member 13 (KCNK13), mRNA [NM_022054] | Hs.510191 |
| A_23_P43157 | 0.02340595 | 1.6847069 | up | MYBL1 | Homo sapiens v-myb avian myeloblastosis viral oncogene homolog-like 1 (MYBL1), transcript variant 1, mRNA [NM_001080416] | Hs.445898 |
| A_23_P158277 | 0.048184037 | 1.6807725 | up | TMCO4 | Homo sapiens transmembrane and coiled-coil domains 4 (TMCO4), mRNA [NM_181719] | Hs.656313 |
| A_33_P3217238 | 6.41E-04 | 1.6807348 | up | ATAD2 | Homo sapiens ATPase family, AAA domain containing 2 (ATAD2), mRNA [NM_014109] | Hs.370834 |
| A_24_P245838 | 0.042756252 | 1.6776348 | up | MGAT3 | Homo sapiens mannosyl (beta-1,4-)-glycoprotein beta-1,4-N-acetylglucosaminyltransferase (MGAT3), transcript variant 1, mRNA [NM_002409] | Hs.276808 |
| A_22_P00022339 | 0.037230145 | 1.6771708 | up |  |  |  |
| A_22_P00015282 | 0.015125311 | 1.6763462 | up | NBPF1 | Homo sapiens neuroblastoma breakpoint family, member 1 (NBPF1), mRNA [NM_017940] | Hs.467587 |
| A_21_P0012064 | 0.019432355 | 1.6756355 | up | LOC102723854 | Homo sapiens uncharacterized LOC102723854 (LOC102723854), long non-coding RNA [NR_110585] | Hs.570165 |
| A_23_P153640 | 0.017359706 | 1.672745 | up | ANKRD27 | Homo sapiens ankyrin repeat domain 27 (VPS9 domain) (ANKRD27), mRNA [NM_032139] | Hs.59236 |
| A_19_P00316933 | 0.045560766 | 1.6684859 | up |  |  |  |
| A_24_P535219 | 0.037306767 | 1.6591967 | up | PHF10 | PHD finger protein 10 [Source:HGNC Symbol;Acc:HGNC:18250] [ENST00000612128] | Hs.435933 |
| A_23_P202496 | 0.028048448 | 1.6572636 | up | NOC3L | Homo sapiens nucleolar complex associated 3 homolog (S. cerevisiae) (NOC3L), mRNA [NM_022451] | Hs.74899 |
| A_33_P3314441 | 0.019934028 | 1.656128 | up | FBXL17 | Homo sapiens F-box and leucine-rich repeat protein 17 (FBXL17), mRNA [NM_001163315] | Hs.657225 |
| A_33_P3240538 | 0.004240521 | 1.6515058 | up | TUBE1 | Homo sapiens tubulin, epsilon 1 (TUBE1), mRNA [NM_016262] | Hs.34851 |
| A_23_P257201 | 0.018813794 | 1.6481588 | up | RNF146 | Homo sapiens ring finger protein 146 (RNF146), transcript variant 2, mRNA [NM_030963] | Hs.267120 |
| A_23_P201319 | 0.041353818 | 1.6379027 | up | DISP1 | Homo sapiens dispatched homolog 1 (Drosophila) (DISP1), mRNA [NM_032890] | Hs.528817 |
| A_22_P00013354 | 0.03940506 | 1.6338058 | up | lnc-RP11-158I9.5.1-1 | UI-H-BI3-ale-d-04-0-UI.s1 NCI_CGAP_Sub5 Homo sapiens cDNA clone IMAGE:2736438 3', mRNA sequence [AW451725] | Hs.674680 |
| A_33_P3232458 | 0.007683395 | 1.627343 | up | CLIP4 | Homo sapiens CAP-GLY domain containing linker protein family, member 4 (CLIP4), transcript variant 1, mRNA [NM_024692] | Hs.122927 |
| A_21_P0000635 | 0.015773254 | 1.6253972 | up | NCAM1-AS1 | Homo sapiens NCAM1 antisense RNA1 (NCAM1-AS1), long non-coding RNA [NR_034101] | Hs.661826 |
| A_23_P334883 | 0.019837996 | 1.6177301 | up | SHANK2 | Homo sapiens SH3 and multiple ankyrin repeat domains 2 (SHANK2), transcript variant 1, mRNA [NM_012309] | Hs.268726 |
| A_19_P00321110 | 0.026177986 | 1.6163149 | up | TIAM2 | T-cell lymphoma invasion and metastasis 2 [Source:HGNC Symbol;Acc:HGNC:11806] [ENST00000449545] | Hs.486886 |
| A_33_P3407549 | 0.004451132 | 1.613366 | up | SOWAHA | Homo sapiens sosondowah ankyrin repeat domain family member A (SOWAHA), mRNA [NM_175873] | Hs.13308 |
| A_22_P00001356 | 0.046204977 | 1.605719 | up |  | DB148555 THYMU3 Homo sapiens cDNA clone THYMU3024557 5', mRNA sequence [DB148555] | Hs.579516 |
| A_33_P3380211 | 0.008904586 | 1.6055889 | up | AKAP9 | Homo sapiens A kinase (PRKA) anchor protein 9 (AKAP9), transcript variant 2, mRNA [NM_005751] | Hs.651221 |
| A_33_P3298771 | 0.04586204 | 1.6045513 | up |  | olfactory receptor, family 52, subfamily L, member 2 pseudogene [Source:HGNC Symbol;Acc:HGNC:14788] [ENST00000572217] | |
| A_33_P3386344 | 0.043064605 | 1.604121 | up | FANCA | Homo sapiens Fanconi anemia, complementation group A (FANCA), transcript variant 2, mRNA [NM_001018112] | Hs.744083 |
| A_22_P00011810 | 0.004924815 | 1.5998803 | up | lnc-PHACTR4-1 | Homo sapiens cDNA FLJ13845 fis, clone THYRO1000815. [AK023907] | Hs.667880 |
| A_33_P3212700 | 0.0474232 | 1.5946438 | up |  |  |  |
| A_24_P212129 | 0.043082666 | 1.5942773 | up | ZNF33A | Homo sapiens zinc finger protein 33A (ZNF33A), transcript variant 2, mRNA [NM_006974] | Hs.435774 |
| A_21_P0006570 | 0.027437545 | 1.5935323 | up | lnc-PLS3-2 | LNCipedia lincRNA (lnc-PLS3-2), lincRNA [lnc-PLS3-2:1] |  |
| A_33_P3415087 | 0.004943086 | 1.5861747 | up | CLCN5 | Homo sapiens chloride channel, voltage-sensitive 5 (CLCN5), transcript variant 1, mRNA [NM_001127899] | Hs.166486 |
| A_32_P389118 | 0.026356598 | 1.5739268 | up | HEATR5B | Homo sapiens HEAT repeat containing 5B (HEATR5B), mRNA [NM_019024] | Hs.744084 |
| A_24_P233878 | 0.005295976 | 1.5682584 | up | GDAP2 | Homo sapiens ganglioside induced differentiation associated protein 2 (GDAP2), transcript variant 1, mRNA [NM_017686] | Hs.594430 |
| A_23_P371129 | 0.02700493 | 1.5644703 | up | SLX4 | Homo sapiens SLX4 structure-specific endonuclease subunit (SLX4), mRNA [NM_032444] | Hs.143681 |
| A_32_P99753 | 0.013733641 | 1.5624636 | up | EFCAB12 | Homo sapiens EF-hand calcium binding domain 12 (EFCAB12), mRNA [NM_207307] | Hs.652347 |
| A_23_P79661 | 0.008281422 | 1.560765 | up | CCDC93 | Homo sapiens coiled-coil domain containing 93 (CCDC93), mRNA [NM_019044] | Hs.107845 |
| A_23_P382775 | 0.013772811 | 1.5606825 | up | BBC3 | Homo sapiens BCL2 binding component 3 (BBC3), transcript variant 4, mRNA [NM_014417] | Hs.467020 |
| A_22_P00003198 | 0.017334966 | 1.5580357 | up | PDP2 | Homo sapiens pyruvate dehyrogenase phosphatase catalytic subunit 2 (PDP2), mRNA [NM_020786] | Hs.632214 |
| A_21_P0000347 | 0.007964208 | 1.5502028 | up | SNORA76C | Homo sapiens small nucleolar RNA, H/ACA box 76C (SNORA76C), small nucleolar RNA [NR_002995] | Hs.405444 |
| A_22_P00009293 | 0.014338791 | 1.5468252 | up | lnc-LRRC32-5 | LNCipedia lincRNA (lnc-LRRC32-5), lincRNA [lnc-LRRC32-5:1] |  |
| A_32_P71113 | 0.04966725 | 1.5352119 | up | SCAI | Homo sapiens suppressor of cancer cell invasion (SCAI), transcript variant 1, mRNA [NM_173690] | Hs.59504 |
| A_23_P14493 | 0.025121942 | 1.5206786 | up | DNAAF2 | Homo sapiens dynein, axonemal, assembly factor 2 (DNAAF2), transcript variant 1, mRNA [NM_018139] | Hs.231761 |
| A_33_P3243168 | 0.011536737 | 1.5191791 | up | MZF1 | Homo sapiens myeloid zinc finger 1 (MZF1), transcript variant 2, mRNA [NM_198055] | Hs.399810 |
| A_23_P68155 | 0.046289254 | 1.5186036 | up | IFIH1 | Homo sapiens interferon induced with helicase C domain 1 (IFIH1), mRNA [NM_022168] | Hs.163173 |
| A_33_P3382835 | 0.02214756 | 1.5165362 | up | NYNRIN | Homo sapiens NYN domain and retroviral integrase containing (NYNRIN), mRNA [NM_025081] | Hs.288348 |
| A_23_P26928 | 0.04515886 | 1.5161977 | up | CCDC103 | family with sequence similarity 187, member A [Source:HGNC Symbol;Acc:HGNC:35153] [ENST00000331733] | Hs.743398 |
| A_33_P3349536 | 0.014882115 | 1.5122488 | up | CHEK1 | Homo sapiens checkpoint kinase 1 (CHEK1), transcript variant 2, mRNA [NM_001114121] | Hs.24529 |
| A_33_P3327921 | 0.04350168 | 1.5054111 | up | RHOQ | Homo sapiens ras homolog family member Q (RHOQ), mRNA [NM_012249] | Hs.709193 |
| A_22_P00010810 | 0.039405778 | 1.5037328 | up | lnc-NMNAT1-2 | LNCipedia lincRNA (lnc-NMNAT1-2), lincRNA [lnc-NMNAT1-2:1] |  |

**(B)**

| **Probe Name** | **p value** | **FC (abs)** | **Regulation** | **Gene Symbol** | **Description** | **UniGeneID** |
| --- | --- | --- | --- | --- | --- | --- |
| A_22_P00000416 | 0.034329794 | 175.4486 | down | lnc-AC069257.9.1-1 | LNCipedia lincRNA (lnc-AC069257.9.1-1), lincRNA [lnc-AC069257.9.1-1:1] | |
| A_33_P3414022 | 0.03841379 | 143.14363 | down | LOC100996724 | PREDICTED: Homo sapiens myomegalin-like (LOC100996724), transcript variant X2, mRNA [XM_003846758] | |
| A_22_P00024323 | 0.0412538 | 128.0631 | down | lnc-C5orf42-3 | LNCipedia lincRNA (lnc-C5orf42-3), lincRNA [lnc-C5orf42-3:3] |  |
| A_24_P887857 | 0.04311484 | 122.80051 | down |  | keratin 17 pseudogene 3 [Source:HGNC Symbol;Acc:HGNC:33697] [ENST00000420566] | |
| A_23_P428373 | 0.04403093 | 120.43224 | down | REXO1L1P | REX1, RNA exonuclease 1 homolog (S. cerevisiae)-like 1, pseudogene [Source:HGNC Symbol;Acc:HGNC:24660] [ENST00000379010] | Hs.373854 |
| A_33_P3283700 | 0.042991247 | 118.729515 | down |  |  |  |
| A_33_P3284132 | 0.042318568 | 112.01533 | down | C10orf90 | Homo sapiens chromosome 10 open reading frame 90 (C10orf90), mRNA [NM_001004298] | Hs.587663 |
| A_33_P3345299 | 0.044041015 | 111.966095 | down |  |  |  |
| A_21_P0004829 | 0.03145308 | 111.32903 | down | lnc-LY86-5 | LNCipedia lincRNA (lnc-LY86-5), lincRNA [lnc-LY86-5:1] |  |
| A_22_P00004739 | 0.017659009 | 109.34994 | down | LOC100287792 | Homo sapiens uncharacterized LOC100287792 (LOC100287792), long non-coding RNA [NR_040021] | Hs.517026 |
| A_21_P0005006 | 0.042740162 | 107.45516 | down | lnc-ATXN1-2 | LNCipedia lincRNA (lnc-ATXN1-2), lincRNA [lnc-ATXN1-2:1] |  |
| A_22_P00011356 | 0.046040114 | 106.75806 | down | LINC00359 | Homo sapiens long intergenic non-protein coding RNA 359 (LINC00359), long non-coding RNA [NR_051966] | Hs.617003 |
| A_33_P3375516 | 0.042852785 | 104.98676 | down | EIF4E1B | Homo sapiens eukaryotic translation initiation factor 4E family member 1B (EIF4E1B), mRNA [NM_001099408] | Hs.448362 |
| A_22_P00016382 | 0.045808975 | 104.8719 | down | lnc-TMEM183A-1 | LNCipedia lincRNA (lnc-TMEM183A-1), lincRNA [lnc-TMEM183A-1:1] |  |
| A_19_P00319558 | 0.022397866 | 104.662544 | down |  |  |  |
| A_24_P190873 | 0.04212247 | 101.82545 | down | FAM163A | Homo sapiens family with sequence similarity 163, member A (FAM163A), mRNA [NM_173509] | Hs.729631 |
| A_33_P3320752 | 0.046867296 | 99.41464 | down |  | Homo sapiens clone HQ0195$ PRO0195 mRNA, complete cds. [AF090901] | |
| A_24_P408704 | 0.020124916 | 96.54098 | down | DOCK2 | Homo sapiens dedicator of cytokinesis 2 (DOCK2), mRNA [NM_004946] | Hs.586174 |
| A_33_P3235677 | 0.04806251 | 91.140915 | down |  | AY465171 POTE14B {Homo sapiens} (exp=-1; wgp=0; cg=0), partial (7%) [THC2775571] | |
| A_21_P0007514 | 0.04473926 | 90.92338 | down | lnc-KCNA1-1 | LNCipedia lincRNA (lnc-KCNA1-1), lincRNA [lnc-KCNA1-1:1] |  |
| A_22_P00014783 | 0.048149407 | 89.793655 | down | lnc-SLC38A8-3 | Homo sapiens full length insert cDNA clone YY79F12. [AF088013] | Hs.621470 |
| A_22_P00000620 | 0.030861061 | 85.60963 | down | lnc-ACOX3-3 | DB026626 TESTI2 Homo sapiens cDNA clone TESTI2008876 5', mRNA sequence [DB026626] | Hs.691340 |
| A_22_P00023258 | 0.002970229 | 84.893394 | down | lnc-AL136115.1-2 | DB071059 TESTI4 Homo sapiens cDNA clone TESTI4013768 5', mRNA sequence [DB071059] | Hs.630976 |
| A_22_P00011775 | 0.049214706 | 84.49667 | down |  | Homo sapiens cDNA FLJ31539 fis, clone NT2RI2000738. [AK056101] | Hs.568047 |
| A_22_P00003571 | 0.044107385 | 82.493416 | down | lnc-CCT5-6 | Q9S1A6_ESCBL (Q9S1A6) Acid phosphatase , partial (5%) [THC2697781] | |
| A_33_P3359763 | 0.04695441 | 82.188225 | down | FLCN | folliculin [Source:HGNC Symbol;Acc:HGNC:27310] [ENST00000466317] | Hs.31652 |
| A_22_P00015105 | 0.043922007 | 82.06662 | down |  |  |  |
| A_21_P0004725 | 0.04918518 | 79.780464 | down |  | PREDICTED: Homo sapiens uncharacterized LOC100506091 (LOC100506091), misc_RNA [XR_158802] | Hs.246334 |
| A_19_P00810227 | 0.036224026 | 76.554344 | down | lnc-C14orf118-1 | LNCipedia lincRNA (lnc-C14orf118-1), lincRNA [lnc-C14orf118-1:4] |  |
| A_21_P0011072 | 0.03779116 | 75.92328 | down |  | long intergenic non-protein coding RNA 943 [Source:HGNC Symbol;Acc:HGNC:48639] [ENST00000537374] | Hs.662298 |
| A_22_P00016163 | 0.037458308 | 73.14731 | down | lnc-TK2-2 | Synthetic construct Homo sapiens gateway clone IMAGE:100021827 3' read CMTM2 mRNA. [CU692975] | |
| A_21_P0013258 | 0.006754242 | 72.99279 | down | XLOC_l2_013532 | BROAD Institute lincRNA (XLOC_l2_013532), lincRNA [TCONS_l2_00026125] | |
| A_22_P00000679 | 0.041909322 | 72.78589 | down | lnc-ADA-1 | LNCipedia lincRNA (lnc-ADA-1), lincRNA [lnc-ADA-1:2] | Hs.671450 |
| A_22_P00006204 | 0.029789692 | 69.81983 | down | XXYLT1-AS2 | Homo sapiens XXYLT1 antisense RNA 2 (XXYLT1-AS2), transcript variant 2, long non-coding RNA [NR_102711] | Hs.658170 |
| A_24_P520767 | 0.012371223 | 67.681046 | down | LOC149351 |  | Hs.546492 |
| A_21_P0011833 | 0.018822456 | 64.063934 | down | LINC01127 | Homo sapiens long intergenic non-protein coding RNA 1127 (LINC01127), long non-coding RNA [NR_103791] | |
| A_21_P0001615 | 0.04658246 | 62.413002 | down | lnc-PLXNA2-1 | LNCipedia lincRNA (lnc-PLXNA2-1), lincRNA [lnc-PLXNA2-1:1] |  |
| A_21_P0008289 | 0.04953568 | 61.24603 | down | BMS1P17 | Homo sapiens BMS1 pseudogene 17 (BMS1P17), non-coding RNA [NR_073460] | Hs.617315 |
| A_33_P3375077 | 0.013446371 | 60.59438 | down | BRPF3 | Homo sapiens bromodomain and PHD finger containing, 3 (BRPF3), mRNA [NM_015695] | Hs.520096 |
| A_21_P0006955 | 0.0414325 | 60.495823 | down | lnc-KLF6-3 | LNCipedia lincRNA (lnc-KLF6-3), lincRNA [lnc-KLF6-3:1] |  |
| A_22_P00003841 | 0.007613188 | 60.121025 | down | lnc-CEACAM18-1 | AY029277 siglec-like protein {Homo sapiens} (exp=-1; wgp=0; cg=0), partial (13%) [THC2773289] | |
| A_21_P0012234 | 0.005831604 | 59.71996 | down |  |  |  |
| A_33_P3301897 | 0.030609027 | 57.301872 | down |  | T cell receptor beta variable 6-7 (non-functional) [Source:HGNC Symbol;Acc:HGNC:12232] [ENST00000390373] | Hs.449373 |
| A_33_P3226825 | 0.005847428 | 55.869442 | down | RLN3 | Homo sapiens relaxin 3 (RLN3), mRNA [NM_080864] | Hs.352155 |
| A_33_P3295056 | 0.0094898 | 55.855705 | down | PTPRCAP | Homo sapiens protein tyrosine phosphatase, receptor type, C-associated protein (PTPRCAP), mRNA [NM_005608] | Hs.155975 |
| A_33_P3263002 | 0.017764496 | 55.80625 | down | TMEM262 | transmembrane protein 262 [Source:HGNC Symbol;Acc:HGNC:49389] [ENST00000528029] | Hs.98170 |
| A_21_P0011177 | 0.029542414 | 55.454987 | down | LINC00539 | Homo sapiens long intergenic non-protein coding RNA 539 (LINC00539), transcript variant 1, long non-coding RNA [NR_103840] | Hs.659198 |
| A_33_P3352467 | 0.039879013 | 55.22445 | down | SSTR2 | Homo sapiens somatostatin receptor 2 (SSTR2), mRNA [NM_001050] | Hs.514451 |
| A_21_P0010872 | 0.006853746 | 55.030655 | down | ANTXRLP1 | Homo sapiens anthrax toxin receptor-like pseudogene 1 (ANTXRLP1), transcript variant 1, non-coding RNA [NR_103827] | Hs.546758 |
| A_33_P3369446 | 0.016114308 | 54.190563 | down | lnc-AC073343.1-1 | LNCipedia lincRNA (lnc-AC073343.1-1), lincRNA [lnc-AC073343.1-1:3] | Hs.730333 |
| A_33_P3386147 | 0.0313582 | 52.951714 | down |  |  |  |
| A_21_P0008796 | 0.03014208 | 52.361862 | down | lnc-RP11-82I10.1.1-2 | LNCipedia lincRNA (lnc-RP11-82I10.1.1-2), lincRNA [lnc-RP11-82I10.1.1-2:1] | |
| A_22_P00005751 | 0.040579896 | 51.494095 | down | lnc-EN2-1 | LNCipedia lincRNA (lnc-EN2-1), lincRNA [lnc-EN2-1:1] |  |
| A_21_P0010011 | 0.011622274 | 51.24517 | down | lnc-FOXA2-7 | LNCipedia lincRNA (lnc-FOXA2-7), lincRNA [lnc-FOXA2-7:3] |  |
| A_22_P00009795 | 0.00518367 | 50.279102 | down |  |  |  |
| A_21_P0006199 | 0.0381166 | 50.043015 | down | lnc-ZNF462-1 | LNCipedia lincRNA (lnc-ZNF462-1), lincRNA [lnc-ZNF462-1:3] |  |
| A_22_P00016464 | 0.046473622 | 49.55781 | down | lnc-TMEM71-1 | RST34058 Athersys RAGE Library Homo sapiens cDNA, mRNA sequence [BG214420] | Hs.679630 |
| A_21_P0006663 | 0.03565586 | 49.47384 | down | LINC01517 | Homo sapiens long intergenic non-protein coding RNA 1517 (LINC01517), long non-coding RNA [NR_120652] | Hs.224866 |
| A_33_P3248405 | 0.011640511 | 49.084644 | down | NRK | Homo sapiens Nik related kinase, mRNA (cDNA clone IMAGE:5722833), complete cds. [BC108702] | Hs.209527 |
| A_21_P0009566 | 0.0039532 | 48.144566 | down | lnc-RNF152-1 | LNCipedia lincRNA (lnc-RNF152-1), lincRNA [lnc-RNF152-1:1] |  |
| A_21_P0009826 | 0.01919714 | 47.996758 | down |  |  |  |
| A_33_P3279426 | 0.003807085 | 47.15432 | down | GIGYF2 | GRB10 interacting GYF protein 2 [Source:HGNC Symbol;Acc:HGNC:11960] [ENST00000458528] | |
| A_33_P3320077 | 0.012841243 | 47.14807 | down | NFIB | Homo sapiens nuclear factor I/B (NFIB), transcript variant 4, mRNA [NM_001282787] | Hs.644095 |
| A_22_P00016364 | 0.006846943 | 47.08231 | down | lnc-TMEM18-1 | LNCipedia lincRNA (lnc-TMEM18-1), lincRNA [lnc-TMEM18-1:1] |  |
| A_33_P3326634 | 0.005367443 | 46.758007 | down | GPC3 | Homo sapiens glypican 3 (GPC3), transcript variant 1, mRNA [NM_001164617] | Hs.644108 |
| A_19_P00804070 | 0.004446724 | 44.90858 | down |  |  |  |
| A_21_P0003872 | 0.008671143 | 44.9041 | down | lnc-ZFP42-7 | LNCipedia lincRNA (lnc-ZFP42-7), lincRNA [lnc-ZFP42-7:3] |  |
| A_22_P00017173 | 0.01879404 | 44.069542 | down | LOC101927740 | Homo sapiens uncharacterized LOC101927740 (LOC101927740), long non-coding RNA [NR_109890] | Hs.738721 |
| A_21_P0009709 | 0.01636045 | 44.038834 | down |  |  |  |
| A_21_P0009759 | 0.027425407 | 44.005527 | down | lnc-TSHZ3-1 | LNCipedia lincRNA (lnc-TSHZ3-1), lincRNA [lnc-TSHZ3-1:1] | Hs.638550 |
| A_21_P0009948 | 0.045665685 | 43.31401 | down | lnc-SSTR4-3 | LNCipedia lincRNA (lnc-SSTR4-3), lincRNA [lnc-SSTR4-3:1] |  |
| A_22_P00002415 | 0.036900774 | 43.204487 | down |  |  |  |
| A_22_P00015370 | 0.048337087 | 42.734882 | down |  |  | Hs.447522 |
| A_22_P00005731 | 0.012595728 | 41.677986 | down | lnc-EMID1-1 | LNCipedia lincRNA (lnc-EMID1-1), lincRNA [lnc-EMID1-1:1] |  |
| A_21_P0002683 | 0.03801003 | 41.06628 | down | lnc-DLX2-1 | LNCipedia lincRNA (lnc-DLX2-1), lincRNA [lnc-DLX2-1:1] |  |
| A_21_P0008247 | 0.044270985 | 40.96228 | down | lnc-USP12-5 | LNCipedia lincRNA (lnc-USP12-5), lincRNA [lnc-USP12-5:1] |  |
| A_21_P0014697 | 6.37E-04 | 40.213047 | down | LOC101060542 | Homo sapiens uncharacterized LOC101060542 (LOC101060542), long non-coding RNA [NR_110764] | Hs.586967 |
| A_21_P0003667 | 0.004007125 | 40.118656 | down | lnc-ARFIP1-1 | LNCipedia lincRNA (lnc-ARFIP1-1), lincRNA [lnc-ARFIP1-1:1] |  |
| A_22_P00020264 | 0.049708527 | 39.25365 | down | LOC101929577 | Homo sapiens uncharacterized LOC101929577 (LOC101929577), long non-coding RNA [NR_125928] | Hs.631131 |
| A_22_P00012196 | 0.002859651 | 38.548412 | down | lnc-POU5F1B-3 | LNCipedia lincRNA (lnc-POU5F1B-3), lincRNA [lnc-POU5F1B-3:3] | Hs.557008 |
| A_22_P00000927 | 0.028729754 | 37.596607 | down | CYP51A1-AS1 | Homo sapiens CYP51A1 antisense RNA 1 (CYP51A1-AS1), transcript variant 1, long non-coding RNA [NR_122109] | Hs.662663 |
| A_33_P3224285 | 0.045367114 | 36.77256 | down |  |  |  |
| A_33_P3234657 | 0.03915025 | 36.449284 | down | PRTG | Homo sapiens protogenin (PRTG), mRNA [NM_173814] | Hs.130957 |
| A_21_P0013133 | 0.047904145 | 35.925976 | down | LOC101928516 | Homo sapiens uncharacterized LOC101928516 (LOC101928516), long non-coding RNA [NR_110856] | Hs.384600 |
| A_22_P00019068 | 0.013086554 | 35.655144 | down | lnc-CLEC14A-2 | LNCipedia lincRNA (lnc-CLEC14A-2), lincRNA [lnc-CLEC14A-2:1] |  |
| A_33_P3347663 | 0.035843257 | 35.3314 | down |  |  | Hs.163898 |
| A_22_P00000887 | 0.00861004 | 34.789032 | down | LOC101928326 | PREDICTED: Homo sapiens uncharacterized LOC101928326 (LOC101928326), ncRNA [XR_245604] | Hs.621262 |
| A_21_P0005361 | 0.046583846 | 34.6385 | down | lnc-IL6-1 | LNCipedia lincRNA (lnc-IL6-1), lincRNA [lnc-IL6-1:1] |  |
| A_22_P00013960 | 0.007265515 | 34.498764 | down | lnc-RTL1-1 | Homo sapiens cDNA FLJ44703 fis, clone BRACE3016788. [AK126659] |  |
| A_23_P6596 | 0.010006152 | 34.231346 | down | HES1 | Homo sapiens hes family bHLH transcription factor 1 (HES1), mRNA [NM_005524] | Hs.250666 |
| A_21_P0007345 | 0.018042922 | 34.207756 | down | lnc-ALX4-1 | LNCipedia lincRNA (lnc-ALX4-1), lincRNA [lnc-ALX4-1:1] |  |
| A_32_P517715 | 0.026174223 | 34.11181 | down | RAD51B | Homo sapiens RAD51 paralog B (RAD51B), transcript variant 3, mRNA [NM_133509] | Hs.172587 |
| A_21_P0014037 | 5.39E-04 | 34.04522 | down |  |  |  |
| A_21_P0008139 | 0.028266553 | 33.50918 | down | LOC102723392 | PREDICTED: Homo sapiens uncharacterized LOC102723392 (LOC102723392), ncRNA [XR_424440] | |
| A_33_P3280009 | 0.023564426 | 33.22454 | down |  |  |  |
| A_22_P00023241 | 0.010599599 | 33.155407 | down | lnc-SLC24A5-3 | Q4HW52_GIBZE (Q4HW52) Predicted protein, partial (12%) [THC2788552] | |
| A_33_P3419711 | 0.02401054 | 33.024277 | down |  | GB |  |
| A_33_P3613516 | 0.036570895 | 32.65584 | down | GATA2-AS1 | Homo sapiens cDNA: FLJ21000 fis, clone CAE03359. [AK024653] | Hs.586109 |
| A_21_P0005280 | 0.031559583 | 32.37233 | down | lnc-GNA12-2 | LNCipedia lincRNA (lnc-GNA12-2), lincRNA [lnc-GNA12-2:1] |  |
| A_21_P0012664 | 0.04684953 | 32.30446 | down | LOC102723415 | PREDICTED: Homo sapiens uncharacterized LOC102723415 (LOC102723415), transcript variant X1, ncRNA [XR_427513] | |
| A_33_P3306762 | 2.16E-04 | 32.2823 | down |  | Q5VZL8_HUMAN (Q5VZL8) Olfactomedin 1, complete [THC2682558] |  |
| A_23_P152838 | 0.049031302 | 32.255497 | down | CCL5 | Homo sapiens chemokine (C-C motif) ligand 5 (CCL5), transcript variant 1, mRNA [NM_002985] | Hs.514821 |
| A_33_P3354783 | 0.033954695 | 31.813719 | down |  |  |  |
| A_22_P00007651 | 0.010378496 | 31.797562 | down | LOC115110 | Homo sapiens uncharacterized LOC115110 (LOC115110), long non-coding RNA [NR_037844] | Hs.602652 |
| A_21_P0005196 | 0.03729361 | 31.664694 | down | LOC101927902 | PREDICTED: Homo sapiens uncharacterized LOC101927902 (LOC101927902), ncRNA [XR_242326] | Hs.561671 |
| A_33_P3384657 | 0.04664795 | 31.607601 | down | ERCC5 | excision repair cross-complementation group 5 [Source:HGNC Symbol;Acc:HGNC:3437] [ENST00000472151] | |
| A_33_P3308716 | 0.028978359 | 31.497911 | down |  | immunoglobulin lambda variable 4-69 [Source:HGNC Symbol;Acc:HGNC:5921] [ENST00000390282] | Hs.742017 |
| A_21_P0012362 | 0.044953734 | 31.49051 | down | XLOC_l2_009613 | BROAD Institute lincRNA (XLOC_l2_009613), lincRNA [TCONS_l2_00018429] | |
| A_22_P00018133 | 0.021468807 | 31.325186 | down | lnc-ZNF503-AS2-1 | LNCipedia lincRNA (lnc-ZNF503-AS2-1), lincRNA [lnc-ZNF503-AS2-1:1] |  |
| A_22_P00013737 | 0.018992871 | 31.318012 | down | LINC01582 | Homo sapiens long intergenic non-protein coding RNA 1582 (LINC01582), long non-coding RNA [NR_120325] | |
| A_21_P0007781 | 0.04812193 | 30.84658 | down | lnc-DHX37-7 | LNCipedia lincRNA (lnc-DHX37-7), lincRNA [lnc-DHX37-7:1] |  |
| A_22_P00008907 | 0.008741743 | 30.57727 | down |  | BX110533 NCI_CGAP_GC6 Homo sapiens cDNA clone IMAGp998D185541, mRNA sequence [BX110533] | Hs.738135 |
| A_22_P00010968 | 0.040712573 | 29.991877 | down |  |  |  |
| A_33_P3410584 | 0.03202415 | 29.659489 | down |  | Homo sapiens cDNA FLJ25208 fis, clone REC05984. [AK057937] |  |
| A_21_P0000050 | 0.041043036 | 29.53783 | down | DISC1 | Homo sapiens disrupted in schizophrenia 1 (DISC1), transcript variant l, mRNA [NM_001164549] | Hs.13318 |
| A_23_P139740 | 0.022412892 | 29.486874 | down | PRMT8 | Homo sapiens protein arginine methyltransferase 8 (PRMT8), transcript variant 1, mRNA [NM_019854] | Hs.504530 |
| A_23_P120982 | 0.009612124 | 29.449862 | down | PKDREJ | Homo sapiens polycystin (PKD) family receptor for egg jelly (PKDREJ), mRNA [NM_006071] | Hs.241383 |
| A_22_P00010046 | 0.019971086 | 29.32786 | down | lnc-MOCS1-1 | IL5-IT0027-121200-327-f08 IT0027 Homo sapiens cDNA, mRNA sequence [BF772485] | Hs.614689 |
| A_23_P216812 | 0.019858139 | 28.734106 | down | CDKN2B | Homo sapiens cyclin-dependent kinase inhibitor 2B (p15, inhibits CDK4) (CDKN2B), transcript variant 1, mRNA [NM_004936] | Hs.72901 |
| A_21_P0002140 | 0.024901936 | 28.597898 | down | lnc-OBSL1-1 | LNCipedia lincRNA (lnc-OBSL1-1), lincRNA [lnc-OBSL1-1:1] |  |
| A_21_P0004639 | 0.04821924 | 28.44069 | down |  |  | Hs.291546 |
| A_21_P0007274 | 0.026285727 | 27.885948 | down | lnc-ZC3H12C-1 | LNCipedia lincRNA (lnc-ZC3H12C-1), lincRNA [lnc-ZC3H12C-1:1] |  |
| A_22_P00005284 | 0.024799366 | 25.7187 | down |  |  |  |
| A_22_P00023512 | 0.035064433 | 25.650463 | down | lnc-RFWD2-2 | DKFZp434A0531_r1 434 (synonym: htes3) Homo sapiens cDNA clone DKFZp434A0531 5', mRNA sequence [AL079817] | Hs.671138 |
| A_21_P0002603 | 0.04176125 | 25.518345 | down | LOC101928427 | PREDICTED: Homo sapiens uncharacterized LOC101928427 (LOC101928427), transcript variant X2, ncRNA [XR_427057] | |
| A_21_P0001366 | 0.04748664 | 25.063223 | down | lnc-TSHB-2 | LNCipedia lincRNA (lnc-TSHB-2), lincRNA [lnc-TSHB-2:1] |  |
| A_23_P102117 | 0.010039387 | 24.829407 | down | WNT10A | Homo sapiens wingless-type MMTV integration site family, member 10A (WNT10A), mRNA [NM_025216] | Hs.121540 |
| A_23_P29663 | 0.0495062 | 24.800947 | down | ZMYND10 | Homo sapiens zinc finger, MYND-type containing 10 (ZMYND10), mRNA [NM_015896] | Hs.526735 |
| A_22_P00013844 | 0.001265982 | 24.774532 | down | LOC102724784 | Homo sapiens uncharacterized LOC102724784 (LOC102724784), long non-coding RNA [NR_120590] | Hs.613276 |
| A_32_P95151 | 0.001790122 | 24.621979 | down |  | Homo sapiens, clone IMAGE:5194137, mRNA. [BC029043] | Hs.207074 |
| A_33_P3248843 | 0.027620554 | 24.049635 | down |  |  |  |
| A_21_P0008974 | 0.047025077 | 23.784164 | down |  |  |  |
| A_33_P3215282 | 0.030118844 | 23.73703 | down | TTBK1 | Homo sapiens tau tubulin kinase 1 (TTBK1), mRNA [NM_032538] | Hs.485436 |
| A_22_P00011842 | 0.018252978 | 23.325987 | down | lnc-PHKB-5 | Homo sapiens cDNA FLJ30342 fis, clone BRACE2007477. [AK054904] | Hs.744137 |
| A_21_P0007148 | 0.0420533 | 23.299425 | down | lnc-APOC3-2 | LNCipedia lincRNA (lnc-APOC3-2), lincRNA [lnc-APOC3-2:1] |  |
| A_21_P0003276 | 0.044219974 | 23.277004 | down | lnc-LRRC33-1 | LNCipedia lincRNA (lnc-LRRC33-1), lincRNA [lnc-LRRC33-1:1] |  |
| A_22_P00008315 | 0.047496036 | 23.275072 | down |  | DA923414 SMINT2 Homo sapiens cDNA clone SMINT2009669 5', mRNA sequence [DA923414] | Hs.573738 |
| A_33_P3374718 | 0.017910594 | 23.2382 | down | WAC-AS1 | Homo sapiens WAC antisense RNA 1 (head to head) (WAC-AS1), long non-coding RNA [NR_033805] | Hs.403253 |
| A_33_P3348927 | 0.038236376 | 23.128075 | down |  |  |  |
| A_23_P13934 | 0.015077262 | 23.001156 | down | FAM222A-AS1 | Homo sapiens FAM222A antisense RNA 1 (FAM222A-AS1), transcript variant 1, long non-coding RNA [NR_026661] | Hs.675961 |
| A_33_P3416218 | 0.001846835 | 22.791397 | down | MUC3A | Homo sapiens mucin 3A, cell surface associated (MUC3A), mRNA [NM_005960] | Hs.744422 |
| A_33_P3327300 | 0.00991316 | 22.610716 | down | NUMBL | numb homolog (Drosophila)-like [Source:HGNC Symbol;Acc:HGNC:8061] [ENST00000598759] | Hs.326953 |
| A_33_P3293169 | 0.030399812 | 22.425463 | down | AMBRA1 | Homo sapiens autophagy/beclin-1 regulator 1 (AMBRA1), transcript variant 4, mRNA [NM_001300731] | Hs.654644 |
| A_22_P00022611 | 0.012344653 | 22.378607 | down | lnc-NBAS-1 | LNCipedia lincRNA (lnc-NBAS-1), lincRNA [lnc-NBAS-1:1] |  |
| A_33_P3276112 | 0.024528014 | 22.164042 | down | lnc-CTD-2517M22.14.1-1 | Homo sapiens cDNA FLJ44789 fis, clone BRACE3038760. [AK126743] | Hs.521937 |
| A_24_P257579 | 0.047400035 | 22.112528 | down | EPB41L4A | Homo sapiens erythrocyte membrane protein band 4.1 like 4A (EPB41L4A), mRNA [NM_022140] | Hs.584954 |
| A_23_P161135 | 0.024506614 | 21.321323 | down | LEPR | Homo sapiens leptin receptor (LEPR), transcript variant 1, mRNA [NM_002303] | Hs.723178 |
| A_33_P3333054 | 0.031026287 | 21.09801 | down | SYBU | Homo sapiens syntabulin (syntaxin-interacting) (SYBU), transcript variant 1, mRNA [NM_001099744] | Hs.390738 |
| A_21_P0007459 | 0.048193578 | 20.903776 | down | CCDC179 | Homo sapiens coiled-coil domain containing 179 (CCDC179), mRNA [NM_001195637] | Hs.555029 |
| A_21_P0010204 | 0.042040057 | 20.624363 | down | LINC00114 | long intergenic non-protein coding RNA 114 [Source:HGNC Symbol;Acc:HGNC:1265] [ENST00000448579] | Hs.278704 |
| A_32_P82111 | 0.0401185 | 20.500889 | down | LRFN2 | leucine rich repeat and fibronectin type III domain containing 2 [Source:HGNC Symbol;Acc:HGNC:21226] [ENST00000338305] | Hs.250015 |
| A_23_P60775 | 0.002432499 | 20.425652 | down | CELF5 | Homo sapiens CUGBP, Elav-like family member 5 (CELF5), transcript variant 1, mRNA [NM_021938] | Hs.655747 |
| A_24_P32394 | 0.02562489 | 20.424652 | down | ZRANB3 | Homo sapiens zinc finger, RAN-binding domain containing 3 (ZRANB3), transcript variant 1, mRNA [NM_032143] | Hs.658422 |
| A_21_P0013484 | 0.007243002 | 20.112585 | down | XLOC_l2_014217 | BROAD Institute lincRNA (XLOC_l2_014217), lincRNA [TCONS_l2_00027777] | |
| A_33_P3261545 | 0.018669222 | 20.11258 | down |  | Homo sapiens cDNA clone IMAGE:4994693. [BC028053] | Hs.631870 |
| A_22_P00019293 | 0.001278074 | 19.666645 | down |  |  |  |
| A_33_P3398187 | 0.021300578 | 19.63473 | down |  |  |  |
| A_33_P3212402 | 0.049877692 | 19.44478 | down | lnc-ZNF479-2 | LNCipedia lincRNA (lnc-ZNF479-2), lincRNA [lnc-ZNF479-2:1] | Hs.568312 |
| A_33_P3400918 | 0.035630263 | 19.413488 | down | DISC1 | Homo sapiens disrupted in schizophrenia 1 (DISC1), transcript variant m, mRNA [NM_001164550] | Hs.13318 |
| A_33_P3364631 | 0.044725753 | 19.312141 | down | lnc-NCKAP1L-1 | LNCipedia lincRNA (lnc-NCKAP1L-1), lincRNA [lnc-NCKAP1L-1:1] |  |
| A_22_P00006314 | 0.008501019 | 19.193077 | down | lnc-FAM91A1-1 | LNCipedia lincRNA (lnc-FAM91A1-1), lincRNA [lnc-FAM91A1-1:1] |  |
| A_22_P00004502 | 0.023644032 | 19.059698 | down | LANCL1-AS1 | Homo sapiens LANCL1 antisense RNA 1 (LANCL1-AS1), transcript variant 1, long non-coding RNA [NR_110604] | |
| A_33_P3315729 | 0.009313568 | 18.658064 | down | ZNF382 | Homo sapiens zinc finger protein 382 (ZNF382), transcript variant 1, mRNA [NM_032825] | Hs.631591 |
| A_22_P00023859 | 0.032706466 | 18.497557 | down | lnc-FAM163B-1 | LNCipedia lincRNA (lnc-FAM163B-1), lincRNA [lnc-FAM163B-1:1] |  |
| A_22_P00000882 | 0.029766578 | 18.471409 | down | RPL34-AS1 | Homo sapiens RPL34 antisense RNA 1 (head to head) (RPL34-AS1), long non-coding RNA [NR_026968] | Hs.683863 |
| A_22_P00000431 | 0.021978764 | 18.362898 | down |  | 602499730F1 NIH_MGC_75 Homo sapiens cDNA clone IMAGE:4613503 5', mRNA sequence [BG431189] | Hs.615336 |
| A_22_P00001589 | 0.049944777 | 18.203968 | down | lnc-ARMCX3-1 | Homo sapiens full length insert cDNA clone YO64F11. [AF085872] | Hs.32118 |
| A_22_P00016610 | 5.50E-04 | 17.989958 | down |  |  |  |
| A_33_P3402304 | 0.04653826 | 17.909805 | down | ZBTB7C | Homo sapiens zinc finger and BTB domain containing 7C (ZBTB7C), mRNA [NM_001039360] | Hs.515388 |
| A_21_P0000483 | 0.01565403 | 17.90384 | down | SNORD75 | Homo sapiens small nucleolar RNA, C/D box 75 (SNORD75), small nucleolar RNA [NR_003941] | |
| A_33_P3631491 | 0.022904815 | 17.88547 | down | lnc-CECR2-1 | Homo sapiens Cat eye syndrome critical region candidate gene number 9 (CECR9) gene, partial sequence. [AF307449] | Hs.542791 |
| A_33_P3412055 | 0.037596244 | 17.775066 | down | ZNF582 | Homo sapiens zinc finger protein 582 (ZNF582), mRNA [NM_144690] | Hs.244391 |
| A_22_P00010608 | 0.033016738 | 17.746782 | down | lnc-NDUFA5-1 | LNCipedia lincRNA (lnc-NDUFA5-1), lincRNA [lnc-NDUFA5-1:5] |  |
| A_22_P00020929 | 0.043953475 | 17.70886 | down | lnc-TRAPPC12-1 | LNCipedia lincRNA (lnc-TRAPPC12-1), lincRNA [lnc-TRAPPC12-1:1] |  |
| A_23_P58328 | 0.015216575 | 17.567432 | down | ANXA10 | Homo sapiens annexin A10 (ANXA10), mRNA [NM_007193] | Hs.188401 |
| A_24_P151032 | 0.031000394 | 17.446186 | down | MYL4 | Homo sapiens myosin, light chain 4, alkali; atrial, embryonic (MYL4), transcript variant 2, mRNA [NM_002476] | Hs.463300 |
| A_23_P150590 | 0.011921153 | 17.336025 | down | SLC22A9 | Homo sapiens solute carrier family 22 (organic anion transporter), member 9 (SLC22A9), mRNA [NM_080866] | Hs.502772 |
| A_21_P0004536 | 0.043700963 | 16.949715 | down | lnc-NEURL1B-3 | LNCipedia lincRNA (lnc-NEURL1B-3), lincRNA [lnc-NEURL1B-3:2] |  |
| A_33_P3311210 | 0.022710374 | 16.943731 | down | BSX | Homo sapiens brain-specific homeobox (BSX), mRNA [NM_001098169] | Hs.449687 |
| A_21_P0013589 | 0.03941188 | 16.926403 | down | LINC01504 | Homo sapiens long intergenic non-protein coding RNA 1504 (LINC01504), transcript variant 2, long non-coding RNA [NR_110953] | |
| A_21_P0012155 | 0.048982028 | 16.761524 | down |  | MIR646 host gene (non-protein coding) [Source:HGNC Symbol;Acc:HGNC:27659] [ENST00000427691] | Hs.542528 |
| A_22_P00008340 | 0.002221887 | 16.750547 | down | LOC100653005 |  | Hs.157039 |
| A_23_P344655 | 0.005652043 | 16.747524 | down | FLJ36848 | Homo sapiens cDNA FLJ36848 fis, clone ASTRO2013802. [AK094167] | Hs.516217 |
| A_22_P00024949 | 0.025565496 | 16.706646 | down | lnc-EIF2AK4-3 | LNCipedia lincRNA (lnc-EIF2AK4-3), lincRNA [lnc-EIF2AK4-3:1] |  |
| A_22_P00013883 | 0.007044932 | 16.57904 | down | lnc-RPRM-5 | LNCipedia lincRNA (lnc-RPRM-5), lincRNA [lnc-RPRM-5:1] |  |
| A_21_P0005101 | 0.03343488 | 16.523285 | down | lnc-WRNIP1-2 | LNCipedia lincRNA (lnc-WRNIP1-2), lincRNA [lnc-WRNIP1-2:20] |  |
| A_22_P00013469 | 0.021812858 | 16.434687 | down | lnc-RP11-324D17.1.1-4 | LNCipedia lincRNA (lnc-RP11-324D17.1.1-4), lincRNA [lnc-RP11-324D17.1.1-4:1] | |
| A_22_P00018643 | 0.008189701 | 16.210241 | down | lnc-TEKT5-2 | BX089002 Soares_testis_NHT Homo sapiens cDNA clone IMAGp998K073476 ; IMAGE:1377150, mRNA sequence [BX089002] | Hs.658699 |
| A_32_P126557 | 0.028233932 | 16.052164 | down | RGS6 | Homo sapiens regulator of G-protein signaling 6 (RGS6), transcript variant 10, mRNA [NM_001204424] | Hs.509872 |
| A_22_P00003716 | 0.047468606 | 15.766782 | down |  |  | Hs.201828 |
| A_22_P00016719 | 0.010475451 | 15.760485 | down | LOC101927793 | PREDICTED: Homo sapiens uncharacterized LOC101927793 (LOC101927793), transcript variant X1, ncRNA [XR_243510] | Hs.690953 |
| A_22_P00002841 | 0.04713421 | 15.64268 | down | lnc-C3orf23-2 | LNCipedia lincRNA (lnc-C3orf23-2), lincRNA [lnc-C3orf23-2:1] |  |
| A_33_P3289810 | 0.026225075 | 15.621835 | down |  | Q4TBJ8_TETNG (Q4TBJ8) Chromosome undetermined SCAF7122, whole genome shotgun sequence. (Fragment), partial (8%) [THC2617349] | |
| A_23_P331770 | 0.027299246 | 15.458671 | down | USP49 | Homo sapiens ubiquitin specific peptidase 49 (USP49), transcript variant 2, mRNA [NM_018561] | Hs.665742 |
| A_24_P352637 | 0.036311887 | 15.393856 | down | TSSK4 | Homo sapiens testis-specific serine kinase 4 (TSSK4), transcript variant 2, mRNA [NM_174944] | Hs.314432 |
| A_21_P0013250 | 0.048610456 | 15.380188 | down | XLOC_l2_013485 | BROAD Institute lincRNA (XLOC_l2_013485), lincRNA [TCONS_l2_00026055] | |
| A_24_P41939 | 0.013614864 | 15.287793 | down |  | keratin 18 pseudogene 24 [Source:HGNC Symbol;Acc:HGNC:33393] [ENST00000344739] | |
| A_21_P0008608 | 0.049240854 | 15.235003 | down | lnc-C15orf41-6 | LNCipedia lincRNA (lnc-C15orf41-6), lincRNA [lnc-C15orf41-6:2] |  |
| A_21_P0002365 | 0.022660933 | 15.091985 | down | lnc-MOGAT1-1 | LNCipedia lincRNA (lnc-MOGAT1-1), lincRNA [lnc-MOGAT1-1:2] |  |
| A_21_P0006077 | 0.02910138 | 14.742535 | down | LOC101927305 | Homo sapiens uncharacterized LOC101927305 (LOC101927305), transcript variant 2, long non-coding RNA [NR_125884] | Hs.737187 |
| A_23_P66967 | 0.035038058 | 14.642153 | down | CETN1 | centrin, EF-hand protein, 1 [Source:HGNC Symbol;Acc:HGNC:1866] [ENST00000327228] | Hs.122511 |
| A_32_P201212 | 0.018314617 | 14.598247 | down | CETN4P | Homo sapiens centrin EF-hand protein 4, pseudogene (CETN4P), non-coding RNA [NR_024041] | Hs.647968 |
| A_33_P3300147 | 0.027350245 | 14.438795 | down | C9orf170 | Homo sapiens chromosome 9 open reading frame 170 (C9orf170), mRNA [NM_001001709] | Hs.657740 |
| A_24_P56388 | 0.020736293 | 14.34986168 | down | HIF1A | Homo sapiens hypoxia inducible factor 1, alpha subunit (basic helix-loop-helix transcription factor) (HIF1A), transcript variant 2, mRNA [NM_181054] | Hs.597216 |
| A_32_P138628 | 0.0097223 | 14.320846 | down | LOC284933 | Homo sapiens uncharacterized LOC284933 (LOC284933), long non-coding RNA [NR_038917] | Hs.159057 |
| A_21_P0005492 | 0.02664686 | 14.287632 | down | lnc-C7orf11-2 | LNCipedia lincRNA (lnc-C7orf11-2), lincRNA [lnc-C7orf11-2:1] |  |
| A_21_P0013873 | 0.029791297 | 14.230369 | down | MAGEA8-AS1 | Homo sapiens MAGEA8 antisense RNA 1 (head to head) (MAGEA8-AS1), long non-coding RNA [NR_102703] | |
| A_22_P00015000 | 0.021279475 | 14.15835 | down | lnc-SMG6-4 | DB081557 TESTI4 Homo sapiens cDNA clone TESTI4027609 5', mRNA sequence [DB081557] | Hs.691518 |
| A_22_P00017639 | 0.029517194 | 13.887967 | down | ST7-AS2 | Homo sapiens ST7 antisense RNA 2 (ST7-AS2), transcript variant 3, long non-coding RNA [NR_109980] | Hs.628891 |
| A_22_P00004092 | 0.03369379 | 13.677367 | down | lnc-CIT-5 | DPEP2_MOUSE (Q8C255) Dipeptidase 2 precursor (Membrane-bound dipeptidase 2) (MBD-2) , partial (4%) [THC2640964] | |
| A_22_P00015696 | 0.007605482 | 13.610501 | down |  | Synthetic construct Homo sapiens gateway clone IMAGE:100019193 3' read WDR89 mRNA. [CU679798] | |
| A_21_P0003446 | 0.046329692 | 13.101422 | down | FLJ36777 | uncharacterized LOC730971 [Source:EntrezGene;Acc:730971] [ENST00000504402] | |
| A_23_P132341 | 0.022566369 | 13.043791 | down | C22orf46 | Homo sapiens chromosome 22 open reading frame 46 (C22orf46), mRNA [NM_001142964] | Hs.517653 |
| A_24_P229884 | 0.006526217 | 13.021189 | down | SMIM22 | Homo sapiens small integral membrane protein 22 (SMIM22), transcript variant 1, mRNA [NM_001253790] | Hs.390599 |
| A_22_P00001606 | 0.04705852 | 12.907559 | down | LOC729040 | PREDICTED: Homo sapiens uncharacterized LOC729040 (LOC729040), misc_RNA [XR_241838] | Hs.678658 |
| A_23_P83234 | 0.034901455 | 12.87309 | down | ZBTB6 | Homo sapiens zinc finger and BTB domain containing 6 (ZBTB6), mRNA [NM_006626] | Hs.654596 |
| A_21_P0006725 | 0.008959985 | 12.77031 | down | LINC00702 | long intergenic non-protein coding RNA 702 [Source:HGNC Symbol;Acc:HGNC:44676] [ENST00000454470] | Hs.741423 |
| A_32_P107029 | 0.042025965 | 12.617078 | down | NAPSA | Homo sapiens napsin A aspartic peptidase (NAPSA), mRNA [NM_004851] | Hs.512843 |
| A_33_P3354424 | 0.008811601 | 12.551085 | down | TRIM61 | Homo sapiens tripartite motif containing 61 (TRIM61), mRNA [NM_001012414] | Hs.654633 |
| A_21_P0004814 | 0.03440151 | 12.5283165 | down | lnc-FOXF2-1 | LNCipedia lincRNA (lnc-FOXF2-1), lincRNA [lnc-FOXF2-1:1] |  |
| A_22_P00009535 | 0.044578753 | 12.508808 | down | LOC440390 | Homo sapiens uncharacterized LOC440390 (LOC440390), long non-coding RNA [NR_126008] | Hs.513789 |
| A_32_P223173 | 0.010794013 | 12.50694 | down | LOC102725053 | PREDICTED: Homo sapiens unconventional myosin-Vb-like (LOC102725053), misc_RNA [XR_432412] | |
| A_22_P00004542 | 0.049195953 | 12.479678 | down | PARD3-AS1 | Homo sapiens PARD3 antisense RNA 1 (PARD3-AS1), long non-coding RNA [NR_108043] | |
| A_22_P00003315 | 0.02955003 | 12.444729 | down | lnc-CAPSL-1 | LNCipedia lincRNA (lnc-CAPSL-1), lincRNA [lnc-CAPSL-1:1] |  |
| A_22_P00015827 | 0.040125165 | 12.41425 | down | LOC339685 | PREDICTED: Homo sapiens uncharacterized LOC339685 (LOC339685), ncRNA [XR_254107] | Hs.434351 |
| A_22_P00001400 | 0.017002534 | 12.37959 | down | BANCR | Homo sapiens BRAF-activated non-protein coding RNA (BANCR), long non-coding RNA [NR_047671] | Hs.49768 |
| A_33_P3298102 | 0.04561479 | 12.29163 | down |  |  |  |
| A_21_P0006865 | 0.019827412 | 12.209282 | down | LINC00840 | Homo sapiens long intergenic non-protein coding RNA 840 (LINC00840), long non-coding RNA [NR_038268] | Hs.462615 |
| A_22_P00014011 | 0.018002257 | 12.03831 | down | LOC100996635 | Homo sapiens uncharacterized LOC100996635 (LOC100996635), transcript variant 2, long non-coding RNA [NR_110622] | |
| A_33_P3233886 | 0.036476135 | 11.986167 | down |  | Sequence 272 from Patent WO0220754. [AX721312] |  |
| A_33_P3364836 | 0.04831032 | 11.917631 | down |  |  |  |
| A_22_P00011229 | 0.03558972 | 11.910232 | down | lnc-OR10H1-1 | LNCipedia lincRNA (lnc-OR10H1-1), lincRNA [lnc-OR10H1-1:1] |  |
| A_23_P100730 | 0.048173293 | 11.871247 | down | SKAP1 | Homo sapiens src kinase associated phosphoprotein 1 (SKAP1), transcript variant 1, mRNA [NM_003726] | Hs.316931 |
| A_33_P3311353 | 0.046619855 | 11.854025 | down | NUTM1 | Homo sapiens NUT midline carcinoma, family member 1 (NUTM1), transcript variant 3, mRNA [NM_175741] | Hs.525769 |
| A_33_P3335183 | 0.033371814 | 11.837058 | down | LRRC37A2 | Homo sapiens leucine rich repeat containing 37, member A2 (LRRC37A2), mRNA [NM_001006607] | Hs.646568 |
| A_33_P3340560 | 0.002570562 | 11.706721 | down | XLOC_l2_015200 | BROAD Institute lincRNA (XLOC_l2_015200), lincRNA [TCONS_l2_00029310] | |
| A_22_P00008722 | 0.026822727 | 11.589672 | down | lnc-KIAA1609-1 | Homo sapiens mRNA; cDNA DKFZp434L231 (from clone DKFZp434L231). [AL137668] | Hs.610979 |
| A_22_P00008750 | 0.005121343 | 11.50042 | down | lnc-KIDINS220-2 | LNCipedia lincRNA (lnc-KIDINS220-2), lincRNA [lnc-KIDINS220-2:4] | Hs.439031 |
| A_22_P00002173 | 0.044708073 | 11.4007845 | down | lnc-BTBD10-5 | LNCipedia lincRNA (lnc-BTBD10-5), lincRNA [lnc-BTBD10-5:1] |  |
| A_33_P3315149 | 0.032291308 | 11.392345 | down | HEXA-AS1 | Homo sapiens HEXA antisense RNA 1 (HEXA-AS1), long non-coding RNA [NR_027262] | Hs.591113 |
| A_21_P0001558 | 0.025471156 | 11.374439 | down | LINC01349 | Homo sapiens long intergenic non-protein coding RNA 1349 (LINC01349), long non-coding RNA [NR_038914] | Hs.536050 |
| A_23_P208768 | 0.034607947 | 11.373835 | down | FCAR | Homo sapiens Fc fragment of IgA, receptor for (FCAR), transcript variant 1, mRNA [NM_002000] | Hs.659872 |
| A_33_P3307945 | 0.029932432 | 11.30445 | down | KIAA2012 | Homo sapiens KIAA2012 (KIAA2012), mRNA [NM_001277372] | Hs.686898 |
| A_33_P3376321 | 0.037119344 | 11.228634 | down | ANK1 | Homo sapiens ankyrin 1, erythrocytic (ANK1), transcript variant 2, mRNA [NM_020477] | Hs.654438 |
| A_33_P3226560 | 0.011707203 | 11.160728 | down |  | HSU07000 breakpoint cluster region protein {Homo sapiens} (exp=-1; wgp=0; cg=0), partial (11%) [THC2481061] | |
| A_21_P0006475 | 9.59E-04 | 11.146289 | down | lnc-SHOX-1 | LNCipedia lincRNA (lnc-SHOX-1), lincRNA [lnc-SHOX-1:1] |  |
| A_21_P0004611 | 0.04530717 | 11.078662 | down | LOC100506188 | Homo sapiens uncharacterized LOC100506188 (LOC100506188), long non-coding RNA [NR_109774] | Hs.485640 |
| A_21_P0002000 | 0.006696583 | 11.071603 | down |  | long intergenic non-protein coding RNA 1248 [Source:EntrezGene;Acc:102723818] [ENST00000453678] | |
| A_22_P00010638 | 0.006314027 | 11.024944 | down |  | Homo sapiens cDNA FLJ40423 fis, clone TESTI2038958. [AK097742] | Hs.528312 |
| A_33_P3271395 | 0.026427202 | 11.004238 | down | LOC100129534 | Homo sapiens small nuclear ribonucleoprotein polypeptide N pseudogene (LOC100129534), non-coding RNA [NR_024489] | Hs.655313 |
| A_22_P00019338 | 0.037173335 | 10.945404 | down |  |  |  |
| A_21_P0012263 | 0.044712625 | 10.922069 | down | XLOC_l2_009332 | BROAD Institute lincRNA (XLOC_l2_009332), lincRNA [TCONS_l2_00017661] | |
| A_33_P3215392 | 0.042526554 | 10.903815 | down | EXOC3L2 | Homo sapiens exocyst complex component 3-like 2 (EXOC3L2), mRNA [NM_138568] | Hs.337557 |
| A_21_P0007613 | 0.005920535 | 10.826179 | down | lnc-C12orf48-3 | LNCipedia lincRNA (lnc-C12orf48-3), lincRNA [lnc-C12orf48-3:1] |  |
| A_21_P0008819 | 0.026088469 | 10.784329 | down | lnc-GOLGA8J-2 | LNCipedia lincRNA (lnc-GOLGA8J-2), lincRNA [lnc-GOLGA8J-2:2] |  |
| A_33_P3252954 | 0.021245081 | 10.74659 | down |  | Homo sapiens cDNA FLJ26166 fis, clone ADG02852. [AK129677] | Hs.240951 |
| A_22_P00005395 | 0.017117416 | 10.656267 | down |  |  | Hs.303788 |
| A_33_P3250555 | 0.039521523 | 10.597946 | down | LOC102723882 | long intergenic non-protein coding RNA 470 [Source:HGNC Symbol;Acc:HGNC:1225] [ENST00000584090] | |
| A_33_P3231700 | 0.031292085 | 10.473327 | down | ZNF677 | Homo sapiens mRNA; cDNA DKFZp686M13108 (from clone DKFZp686M13108). [BX648243] | Hs.20506 |
| A_22_P00000214 | 0.003542783 | 10.46168 | down | LIG3 | ligase III, DNA, ATP-dependent [Source:HGNC Symbol;Acc:HGNC:6600] [ENST00000378526] | Hs.594059 |
| A_22_P00014344 | 0.02544574 | 10.452206 | down |  | long intergenic non-protein coding RNA 1330 [Source:HGNC Symbol;Acc:HGNC:50536] [ENST00000493529] | |
| A_19_P00804072 | 0.01424543 | 10.400594 | down | RNF213 | Homo sapiens ring finger protein 213 (RNF213), transcript variant 3, mRNA [NM_001256071] | Hs.740662 |
| A_21_P0010936 | 0.040901143 | 10.382053 | down |  |  |  |
| A_22_P00024162 | 0.013090013 | 10.243933 | down | lnc-SLC3A2-1 | LNCipedia lincRNA (lnc-SLC3A2-1), lincRNA [lnc-SLC3A2-1:1] |  |
| A_21_P0003059 | 0.045792785 | 10.219613 | down | lnc-SKIL-1 | LNCipedia lincRNA (lnc-SKIL-1), lincRNA [lnc-SKIL-1:1] |  |
| A_23_P47806 | 0.038428724 | 10.181433 | down | MIP | Homo sapiens major intrinsic protein of lens fiber (MIP), mRNA [NM_012064] | Hs.574026 |
| A_33_P3344276 | 0.031846143 | 10.17318 | down | HS1BP3 | Homo sapiens HCLS1 binding protein 3 (HS1BP3), mRNA [NM_022460] | Hs.531785 |
| A_32_P15512 | 0.034902852 | 10.1617 | down | C1orf194 | Homo sapiens chromosome 1 open reading frame 194 (C1orf194), mRNA [NM_001122961] | Hs.446962 |
| A_22_P00000286 | 0.034191046 | 10.113776 | down |  |  |  |
| A_22_P00010216 | 0.018431652 | 10.106906 | down |  | BX119863 Soares_NSF_F8_9W_OT_PA_P_S1 Homo sapiens cDNA clone IMAGp998F015873 ; IMAGE:2365824, mRNA sequence [BX119863] | Hs.738422 |
| A_33_P3354191 | 0.0408441 | 10.03036 | down |  | upstream binding transcription factor, RNA polymerase I-like 9 (pseudogene) [Source:HGNC Symbol;Acc:HGNC:50286] [ENST00000534504] | |
| A_23_P55373 | 0.004121782 | 9.895525 | down | ALOX15 | Homo sapiens arachidonate 15-lipoxygenase (ALOX15), mRNA [NM_001140] | Hs.73809 |
| A_23_P57733 | 0.00559932 | 9.8122225 | down | DRD3 | Homo sapiens dopamine receptor D3 (DRD3), transcript variant e, mRNA [NM_033663] | Hs.121478 |
| A_24_P221092 | 9.92E-04 | 9.797181 | down |  | Homo sapiens clone pp9953 unknown mRNA. [AF289615] | Hs.684473 |
| A_33_P3372285 | 0.02644181 | 9.757426 | down | ZNF81 | Homo sapiens zinc finger protein 81 (ZNF81), mRNA [NM_007137] | Hs.114246 |
| A_21_P0012133 | 0.019325742 | 9.735021 | down | SIRPB2 | signal-regulatory protein beta 2 [Source:HGNC Symbol;Acc:HGNC:16247] [ENST00000486775] | |
| A_21_P0004560 | 0.04909147 | 9.682967 | down | lnc-ZNF366-2 | LNCipedia lincRNA (lnc-ZNF366-2), lincRNA [lnc-ZNF366-2:2] |  |
| A_21_P0013354 | 0.017838053 | 9.559604 | down | ZNF767P | Homo sapiens zinc finger family member 767, pseudogene (ZNF767P), transcript variant 2, non-coding RNA [NR_027789] | Hs.520785 |
| A_24_P31627 | 0.008103236 | 9.5139885 | down | KCNB1 | Homo sapiens potassium channel, voltage gated Shab related subfamily B, member 1 (KCNB1), mRNA [NM_004975] | Hs.84244 |
| A_22_P00004770 | 0.002491124 | 9.487043 | down | lnc-CTU2-1 | Homo sapiens cDNA FLJ40532 fis, clone TESTI2047801. [AK097851] | Hs.377001 |
| A_21_P0013611 | 0.022457482 | 9.393341 | down | XLOC_l2_015090 | BROAD Institute lincRNA (XLOC_l2_015090), lincRNA [TCONS_l2_00029161] | |
| A_21_P0002118 | 0.016759057 | 9.362226 | down |  | qe05g10.x1 Soares_testis_NHT Homo sapiens cDNA clone IMAGE:1738146 3', mRNA sequence [AI140623] | Hs.580118 |
| A_33_P3356926 | 0.027174465 | 9.3155775 | down | APBB3 | Homo sapiens amyloid beta (A4) precursor protein-binding, family B, member 3 (APBB3), transcript variant 1, mRNA [NM_133174] | Hs.529449 |
| A_22_P00010304 | 0.022500968 | 9.148056 | down | lnc-MTX1-1 | LNCipedia lincRNA (lnc-MTX1-1), lincRNA [lnc-MTX1-1:6] | Hs.658188 |
| A_22_P00018969 | 0.030164339 | 9.121935 | down | lnc-SLC25A47-4 | LNCipedia lincRNA (lnc-SLC25A47-4), lincRNA [lnc-SLC25A47-4:2] |  |
| A_21_P0009733 | 0.046172325 | 9.047063 | down | lnc-CTD-2368P22.1.1-1 | LNCipedia lincRNA (lnc-CTD-2368P22.1.1-1), lincRNA [lnc-CTD-2368P22.1.1-1:1] | |
| A_33_P3416937 | 0.013973871 | 8.971583 | down | FAM206A | family with sequence similarity 206, member A [Source:HGNC Symbol;Acc:HGNC:1364] [ENST00000374624] | Hs.736735 |
| A_22_P00012657 | 0.009459063 | 8.898583 | down | LINCR-0002 | Homo sapiens uncharacterized LincR-0002 (LINCR-0002), long non-coding RNA [NR_120606] | |
| A_22_P00007110 | 0.029475957 | 8.888432 | down | lnc-GLTP-1 | LNCipedia lincRNA (lnc-GLTP-1), lincRNA [lnc-GLTP-1:1] |  |
| A_33_P3299232 | 0.045742407 | 8.841528 | down | BMS1P20 | Homo sapiens BMS1 pseudogene 20 (BMS1P20), non-coding RNA [NR_027293] | Hs.449601 |
| A_22_P00002812 | 0.044675507 | 8.83201 | down | HS1BP3-IT1 | Homo sapiens HS1BP3 intronic transcript 1 (non-protein coding) (HS1BP3-IT1), long non-coding RNA [NR_046836] | |
| A_33_P3714482 | 0.039481353 | 8.820332 | down | LRP5L | Homo sapiens low density lipoprotein receptor-related protein 5-like (LRP5L), transcript variant 2, mRNA [NM_001135772] | Hs.634058 |
| A_22_P00018241 | 0.035809875 | 8.69133 | down | LOC101927905 | Homo sapiens uncharacterized LOC101927905 (LOC101927905), transcript variant 1, long non-coding RNA [NR_120454] | |
| A_21_P0005609 | 0.001494845 | 8.5887785 | down | KMT2E-AS1 | Homo sapiens KMT2E antisense RNA 1 (head to head) (KMT2E-AS1), long non-coding RNA [NR_024586] | Hs.631150 |
| A_33_P3222056 | 0.037925825 | 8.583718 | down | lnc-RP11-148O21.2.1-1 | LNCipedia lincRNA (lnc-RP11-148O21.2.1-1), lincRNA [lnc-RP11-148O21.2.1-1:1] | |
| A_23_P416965 | 0.0301638 | 8.541349 | down | FAM149A | Homo sapiens family with sequence similarity 149, member A (FAM149A), transcript variant 1, mRNA [NM_015398] | Hs.744961 |
| A_32_P114746 | 0.012058306 | 8.53977 | down | C1orf111 | Homo sapiens chromosome 1 open reading frame 111 (C1orf111), mRNA [NM_182581] | Hs.97784 |
| A_23_P90497 | 0.039770707 | 8.520908 | down | LILRA4 | Homo sapiens leukocyte immunoglobulin-like receptor, subfamily A (with TM domain), member 4 (LILRA4), mRNA [NM_012276] | Hs.406708 |
| A_23_P68234 | 0.04115779 | 8.415446 | down | GPR75 | Homo sapiens G protein-coupled receptor 75 (GPR75), mRNA [NM_006794] | Hs.40763 |
| A_22_P00013747 | 0.045884855 | 8.382268 | down | lnc-RP11-863N1.2.1-3 | LNCipedia lincRNA (lnc-RP11-863N1.2.1-3), lincRNA [lnc-RP11-863N1.2.1-3:1] | |
| A_22_P00013843 | 0.036005598 | 8.330998 | down | lnc-RPL27A-1 | DB099988 TESTI4 Homo sapiens cDNA clone TESTI4051795 5', mRNA sequence [DB099988] | Hs.630770 |
| A_24_P49896 | 0.003211864 | 8.330597 | down | GDPD4 | Homo sapiens glycerophosphodiester phosphodiesterase domain containing 4 (GDPD4), mRNA [NM_182833] | Hs.249795 |
| A_21_P0007213 | 0.030801805 | 8.313602 | down | lnc-MS4A8B-1 | LNCipedia lincRNA (lnc-MS4A8B-1), lincRNA [lnc-MS4A8B-1:7] |  |
| A_23_P134729 | 0.02582068 | 8.244049 | down | RBM12B-AS1 | Homo sapiens RBM12B antisense RNA 1 (RBM12B-AS1), long non-coding RNA [NR_027259] | Hs.192788 |
| A_22_P00013837 | 0.03997825 | 8.180136 | down | USP12-AS1 | Homo sapiens USP12 antisense RNA 1 (USP12-AS1), long non-coding RNA [NR_046547] | |
| A_33_P3293254 | 0.024647959 | 8.179046 | down | GPSM1 | Homo sapiens G-protein signaling modulator 1 (GPSM1), transcript variant 2, mRNA [NM_015597] | Hs.239370 |
| A_22_P00007407 | 0.04492731 | 8.173152 | down |  | Homo sapiens cDNA FLJ31183 fis, clone KIDNE2000317. [AK055745] | Hs.473947 |
| A_23_P45011 | 0.004447395 | 8.10425 | down | PPP1R14C | Homo sapiens protein phosphatase 1, regulatory (inhibitor) subunit 14C (PPP1R14C), mRNA [NM_030949] | Hs.486798 |
| A_22_P00013296 | 0.005463834 | 8.011385 | down | lnc-RP11-1105G2.3.1-1 | CR737344 NCI_CGAP_Co8 Homo sapiens cDNA clone IMAGp971C1183 ; IMAGE:1927187 5', mRNA sequence [CR737344] | Hs.640490 |
| A_22_P00018477 | 0.013438611 | 7.9843116 | down | lnc-THOC5-1 | DA084938 BRACE2 Homo sapiens cDNA clone BRACE2039191 5', mRNA sequence [DA084938] | Hs.716569 |
| A_22_P00025061 | 0.017077394 | 7.8646708 | down | lnc-AC078802.1-1 | LNCipedia lincRNA (lnc-AC078802.1-1), lincRNA [lnc-AC078802.1-1:1] |  |
| A_33_P3276301 | 0.032269944 | 7.843606 | down |  | T cell receptor beta variable 11-1 [Source:HGNC Symbol;Acc:HGNC:12180] [ENST00000390367] | Hs.546371 |
| A_21_P0005069 | 0.042433698 | 7.834064 | down | lnc-ULBP3-1 | LNCipedia lincRNA (lnc-ULBP3-1), lincRNA [lnc-ULBP3-1:1] |  |
| A_22_P00001551 | 0.044306707 | 7.7069974 | down | lnc-ARID1B-1 | LNCipedia lincRNA (lnc-ARID1B-1), lincRNA [lnc-ARID1B-1:1] |  |
| A_24_P100650 | 0.028741553 | 7.7018676 | down | EQTN | Homo sapiens equatorin, sperm acrosome associated (EQTN), transcript variant 1, mRNA [NM_020641] | Hs.163070 |
| A_33_P3385161 | 0.04150463 | 7.653832 | down | EFCAB9 | Homo sapiens EF-hand calcium binding domain 9 (EFCAB9), mRNA [NM_001171183] | Hs.716824 |
| A_21_P0005388 | 0.046304032 | 7.607678 | down | lnc-ZNF727-2 | LNCipedia lincRNA (lnc-ZNF727-2), lincRNA [lnc-ZNF727-2:1] |  |
| A_33_P3297883 | 0.040469624 | 7.5898232 | down | LOC100131796 | Homo sapiens LP2570 mRNA, complete cds. [AY203937] |  |
| A_21_P0011336 | 0.029160064 | 7.5532937 | down | LOC440300 | Homo sapiens chondroitin sulfate proteoglycan 4 pseudogene (LOC440300), non-coding RNA [NR_033738] | Hs.546565 |
| A_21_P0010592 | 0.011068056 | 7.52661 | down | LOC100288069 | Homo sapiens uncharacterized LOC100288069 (LOC100288069), long non-coding RNA [NR_033908] | Hs.527521 |
| A_21_P0011228 | 0.041590355 | 7.5091243 | down |  | long intergenic non-protein coding RNA 444 [Source:HGNC Symbol;Acc:HGNC:42781] [ENST00000435617] | Hs.733716 |
| A_23_P161998 | 0.016791614 | 7.4867845 | down | HPX | Homo sapiens hemopexin (HPX), mRNA [NM_000613] | Hs.426485 |
| A_21_P0003673 | 0.02543031 | 7.4766345 | down | lnc-PALLD-1 | LNCipedia lincRNA (lnc-PALLD-1), lincRNA [lnc-PALLD-1:1] |  |
| A_21_P0014059 | 0.04719139 | 7.433264 | down | PIGC | phosphatidylinositol glycan anchor biosynthesis, class C [Source:HGNC Symbol;Acc:HGNC:8960] [ENST00000489002] | Hs.728951 |
| A_33_P3320443 | 0.02654979 | 7.4200907 | down | MGC57346-CRHR1 | Homo sapiens MGC57346-CRHR1 readthrough (MGC57346-CRHR1), transcript variant 5, mRNA [NM_001256299] | Hs.417628 |
| A_21_P0013627 | 0.030399852 | 7.409437 | down | LOC730098 | PREDICTED: Homo sapiens uncharacterized LOC730098 (LOC730098), transcript variant X1, misc_RNA [XR_113195] | Hs.459590 |
| A_22_P00017050 | 0.04423696 | 7.381656 | down | lnc-TUBA1C-1 | zr02f09.s1 Stratagene NT2 neuronal precursor 937230 Homo sapiens cDNA clone IMAGE:650345 3' similar to gb:K00558 TUBULIN ALPHA-1 CHAIN (HUMAN);, mRNA sequence [AA218968] | Hs.731608 |
| A_33_P3371727 | 0.026337262 | 7.347566 | down | SAT1 | spermidine/spermine N1-acetyltransferase 1 [Source:HGNC Symbol;Acc:HGNC:10540] [ENST00000379253] | Hs.28491 |
| A_22_P00009753 | 0.010360392 | 7.314235 | down |  | 603295654F1 NIH_MGC_96 Homo sapiens cDNA clone IMAGE:5315076 5', mRNA sequence [BI668294] | Hs.576199 |
| A_33_P3660204 | 0.036526784 | 7.2942553 | down | C5orf66-AS2 | Homo sapiens C5orf66 antisense RNA 2 (C5orf66-AS2), long non-coding RNA [NR_037895] | Hs.434422 |
| A_33_P3309289 | 0.010539617 | 7.2392426 | down | ST3GAL4 | Homo sapiens cDNA FLJ11867 fis, clone HEMBA1006976, weakly similar to H.sapiens mRNA for Gal-beta(1-3/1-4)GlcNAc alpha-2.3-sialyltransferase. [AK021929] | |
| A_22_P00022392 | 0.022772713 | 7.2368226 | down | lnc-GTDC1-3 | Q607B0_METCA (Q607B0) Transcription elongation factor (Transcript cleavage factor), partial (8%) [THC2756937] | |
| A_33_P3264790 | 0.009007346 | 7.217511 | down | GJD4 | Homo sapiens gap junction protein, delta 4, 40.1kDa, mRNA (cDNA clone IMAGE:5240397), partial cds. [BC035898] | Hs.638922 |
| A_23_P130961 | 0.043846134 | 7.1038594 | down | ELANE | Homo sapiens elastase, neutrophil expressed (ELANE), mRNA [NM_001972] | Hs.99863 |
| A_23_P64888 | 0.029409904 | 7.0971136 | down | TAS2R10 | Homo sapiens taste receptor, type 2, member 10 (TAS2R10), mRNA [NM_023921] | Hs.533756 |
| A_33_P3309626 | 0.04626951 | 7.0613937 | down | ADAMTS17 | ADAM metallopeptidase with thrombospondin type 1 motif, 17 [Source:HGNC Symbol;Acc:HGNC:17109] [ENST00000378898] | Hs.513200 |
| A_22_P00013923 | 0.046671424 | 7.0592737 | down | LOC101927755 | Homo sapiens uncharacterized LOC101927755 (LOC101927755), transcript variant 2, long non-coding RNA [NR_110816] | Hs.733307 |
| A_33_P3310744 | 0.012277701 | 6.9783297 | down | TTC34 | Homo sapiens tetratricopeptide repeat domain 34 (TTC34), mRNA [NM_001242672] | Hs.632363 |
| A_21_P0009991 | 0.024869466 | 6.886049 | down | lnc-COL20A1-3 | LNCipedia lincRNA (lnc-COL20A1-3), lincRNA [lnc-COL20A1-3:1] |  |
| A_23_P368338 | 0.008727008 | 6.876917 | down | TRAPPC3L | Homo sapiens trafficking protein particle complex 3-like (TRAPPC3L), mRNA [NM_001139444] | Hs.134795 |
| A_33_P3398181 | 0.01801451 | 6.8538656 | down | LOC100127909 | Homo sapiens cDNA FLJ26912 fis, clone RCT02392. [AK130422] | Hs.637202 |
| A_33_P3365710 | 0.024322854 | 6.826296 | down | ATP6V0D1 | ATPase, H+ transporting, lysosomal 38kDa, V0 subunit d1 [Source:HGNC Symbol;Acc:HGNC:13724] [ENST00000568298] | |
| A_22_P00014715 | 5.62E-04 | 6.822033 | down | lnc-SLC25A5-1 | LNCipedia lincRNA (lnc-SLC25A5-1), lincRNA [lnc-SLC25A5-1:1] |  |
| A_22_P00002061 | 5.81E-04 | 6.8206735 | down | lnc-BLID-1 | LNCipedia lincRNA (lnc-BLID-1), lincRNA [lnc-BLID-1:8] | Hs.44098 |
| A_19_P00322663 | 0.044870555 | 6.718934 | down |  | Q21WG5_9BURK (Q21WG5) Transcriptional regulator, MarR family, partial (10%) [THC2712539] | |
| A_22_P00010636 | 0.025576133 | 6.5892086 | down | LOC100506351 | PREDICTED: Homo sapiens uncharacterized LOC100506351 (LOC100506351), ncRNA [XR_108863] | Hs.666202 |
| A_33_P3342300 | 0.026206978 | 6.583317 | down | VSX1 | Homo sapiens visual system homeobox 1 (VSX1), transcript variant 4, mRNA [NM_001256272] | Hs.274264 |
| A_33_P3314659 | 0.03851014 | 6.577877 | down | SPEF2 | Homo sapiens sperm flagellar 2 (SPEF2), transcript variant 2, mRNA [NM_144722] | Hs.298863 |
| A_33_P3341144 | 0.011492315 | 6.489272377 | down | BAX | Homo sapiens BCL2-associated X protein (BAX), transcript variant beta, mRNA [NM_004324] | Hs.624291 |
| A_23_P322008 | 0.024608636 | 6.46542 | down | TRHR | Homo sapiens thyrotropin-releasing hormone receptor (TRHR), mRNA [NM_003301] | Hs.3022 |
| A_22_P00013880 | 0.046897292 | 6.4360523 | down |  |  |  |
| A_33_P3286774 | 0.04244751 | 6.3470826 | down |  | phosphodiesterase 4D interacting protein [Source:HGNC Symbol;Acc:HGNC:15580] [ENST00000479369] | Hs.731111 |
| A_22_P00015995 | 0.044522904 | 6.3236356 | down | TEX261 | Homo sapiens testis expressed 261 (TEX261), mRNA [NM_144582] | Hs.516087 |
| A_21_P0000554 | 0.007376413 | 6.3134108 | down | KALRN | Homo sapiens kalirin, RhoGEF kinase (KALRN), transcript variant 4, non-coding RNA [NR_028136] | Hs.8004 |
| A_21_P0008575 | 0.026196022 | 6.246887 | down | lnc-ALDH1A3-1 | Homo sapiens cDNA FLJ31972 fis, clone NT2RP7008142. [AK056534] | Hs.612155 |
| A_21_P0005936 | 0.019196544 | 6.2395587 | down | lnc-UNC5D-1 | LNCipedia lincRNA (lnc-UNC5D-1), lincRNA [lnc-UNC5D-1:2] |  |
| A_33_P3280875 | 0.027960343 | 6.2054267 | down | IVNS1ABP | Homo sapiens influenza virus NS1A binding protein (IVNS1ABP), mRNA [NM_006469] | Hs.497183 |
| A_21_P0007903 | 0.007968095 | 6.1913204 | down | lnc-SLC15A4-2 | LNCipedia lincRNA (lnc-SLC15A4-2), lincRNA [lnc-SLC15A4-2:2] |  |
| A_32_P525524 | 0.040771924 | 6.1695976 | down | ITPRIPL1 | Homo sapiens inositol 1,4,5-trisphosphate receptor interacting protein-like 1 (ITPRIPL1), transcript variant 1, mRNA [NM_178495] | Hs.65009 |
| A_21_P0011080 | 0.022754038 | 6.129361 | down | XLOC_l2_003285 | BROAD Institute lincRNA (XLOC_l2_003285), lincRNA [TCONS_l2_00006102] | Hs.123633 |
| A_22_P00013950 | 0.027157297 | 6.1112595 | down | LOC101928255 | PREDICTED: Homo sapiens uncharacterized LOC101928255 (LOC101928255), ncRNA [XR_244322] | Hs.399886 |
| A_21_P0008582 | 0.019352878 | 6.0990415 | down | lnc-OR4N4-2 | LNCipedia lincRNA (lnc-OR4N4-2), lincRNA [lnc-OR4N4-2:1] |  |
| A_33_P3336567 | 0.0295955 | 6.0858307 | down |  |  |  |
| A_33_P6805502 | 0.04223927 | 6.0020156 | down | LINC00671 | Homo sapiens long intergenic non-protein coding RNA 671 (LINC00671), long non-coding RNA [NR_027254] | Hs.632257 |
| A_21_P0009147 | 0.033801977 | 6.0010605 | down | LINC00672 | Homo sapiens long intergenic non-protein coding RNA 672 (LINC00672), long non-coding RNA [NR_038847] | Hs.634043 |
| A_22_P00007202 | 0.026487056 | 5.9880185 | down | lnc-GPBP1L1-1 | AGENCOURT_37009809 NIH_MGC_280 Homo sapiens cDNA clone IMAGE:7505078 5', mRNA sequence [CV800748] | Hs.657748 |
| A_22_P00003069 | 0.013459506 | 5.981636 | down |  |  |  |
| A_33_P3259440 | 0.039126348 | 5.9497185 | down | GOLGA6A | Homo sapiens golgin A6 family, member A (GOLGA6A), mRNA [NM_001038640] | Hs.546408 |
| A_21_P0007340 | 0.034931876 | 5.945499 | down | LOC100507144 | Homo sapiens uncharacterized LOC100507144 (LOC100507144), long non-coding RNA [NR_120528] | Hs.673491 |
| A_23_P332584 | 0.020591957 | 5.8985376 | down | KIAA1107 | Homo sapiens KIAA1107 (KIAA1107), mRNA [NM_015237] | Hs.21554 |
| A_22_P00016647 | 0.040506274 | 5.851237 | down |  | hw35e08.y1 Human primary human ocular pericytes. Unamplified (hw) Homo sapiens cDNA clone hw35e08 5', mRNA sequence [CN483642] | Hs.577143 |
| A_21_P0003236 | 0.018717 | 5.7847037 | down | lnc-ROBO2-4 | LNCipedia lincRNA (lnc-ROBO2-4), lincRNA [lnc-ROBO2-4:3] |  |
| A_22_P00014453 | 0.020552536 | 5.7656565 | down |  | PREDICTED: Homo sapiens uncharacterized LOC100506851 (LOC100506851), ncRNA [XR_108264] | Hs.435465 |
| A_33_P3256920 | 0.029300338 | 5.7390985 | down | WNT7B | Homo sapiens wingless-type MMTV integration site family, member 7B (WNT7B), mRNA [NM_058238] | Hs.512714 |
| A_22_P00006159 | 0.002310545 | 5.701187 | down | LOC102724842 | PREDICTED: Homo sapiens uncharacterized LOC102724842 (LOC102724842), transcript variant X1, ncRNA [XR_425797] | Hs.602583 |
| A_22_P00003850 | 0.025978746 | 5.6429777 | down | LINC01272 | Homo sapiens long intergenic non-protein coding RNA 1272 (LINC01272), mRNA [NM_001278655] | Hs.235484 |
| A_24_P323941 | 0.013277653 | 5.6326385 | down | FAM209A | Homo sapiens family with sequence similarity 209, member A (FAM209A), mRNA [NM_001012971] | Hs.504907 |
| A_23_P359636 | 0.015441425 | 5.605233 | down | BROX | Homo sapiens BRO1 domain and CAAX motif containing (BROX), transcript variant 1, mRNA [NM_144695] | Hs.552608 |
| A_23_P134419 | 0.040036645 | 5.529554 | down | ZP3 | Homo sapiens zona pellucida glycoprotein 3 (sperm receptor) (ZP3), transcript variant 2, mRNA [NM_007155] | Hs.656137 |
| A_21_P0012274 | 0.03963468 | 5.5071416 | down | LINC01296 | Homo sapiens long intergenic non-protein coding RNA 1296 (LINC01296), transcript variant 2, long non-coding RNA [NR_122112] | Hs.744284 |
| A_22_P00014589 | 0.003899602 | 5.4684143 | down | lnc-SLC12A7-1 | LNCipedia lincRNA (lnc-SLC12A7-1), lincRNA [lnc-SLC12A7-1:16] |  |
| A_21_P0005137 | 0.03433641 | 5.409602 | down | lnc-C6orf115-1 | LNCipedia lincRNA (lnc-C6orf115-1), lincRNA [lnc-C6orf115-1:1] |  |
| A_22_P00009733 | 0.038953185 | 5.394312 | down | LINC01393 | Homo sapiens long intergenic non-protein coding RNA 1393 (LINC01393), long non-coding RNA [NR_120521] | Hs.352357 |
| A_33_P3239787 | 0.014683499 | 5.3679447 | down | ZNF646 | Homo sapiens zinc finger protein 646 (ZNF646), mRNA [NM_014699] | Hs.119273 |
| A_23_P428366 | 0.02524091 | 5.3192644 | down | HORMAD2 | Homo sapiens HORMA domain containing 2 (HORMAD2), mRNA [NM_152510] | Hs.120391 |
| A_22_P00005014 | 0.03303014 | 5.297833 | down | LOC643355 | Homo sapiens uncharacterized LOC643355 (LOC643355), mRNA [NM_001270945] | Hs.279714 |
| A_22_P00009054 | 0.025469745 | 5.2868524 | down | lnc-LEKR1-4 | LNCipedia lincRNA (lnc-LEKR1-4), lincRNA [lnc-LEKR1-4:4] |  |
| A_24_P330633 | 0.046675302 | 5.283522 | down | TAT | Homo sapiens tyrosine aminotransferase (TAT), mRNA [NM_000353] | Hs.161640 |
| A_33_P3408757 | 0.042844377 | 5.2202125 | down | FOXO6 | Homo sapiens forkhead box O6 (FOXO6), mRNA [NM_001291281] |  |
| A_22_P00001310 | 0.001454597 | 5.199111 | down | lnc-ANP32A-2 | LNCipedia lincRNA (lnc-ANP32A-2), lincRNA [lnc-ANP32A-2:2] | Hs.631231 |
| A_23_P376172 | 0.004490821 | 5.136419 | down | C1orf64 | Homo sapiens chromosome 1 open reading frame 64 (C1orf64), mRNA [NM_178840] | Hs.29190 |
| A_21_P0006078 | 0.03941517 | 5.123021 | down | LINC01410 | Homo sapiens long intergenic non-protein coding RNA 1410 (LINC01410), long non-coding RNA [NR_121647] | Hs.351215 |
| A_21_P0010335 | 0.03359667 | 5.1168337 | down | LOC100506271 | Homo sapiens uncharacterized LOC100506271 (LOC100506271), long non-coding RNA [NR_110515] | Hs.220558 |
| A_21_P0001417 | 0.02745125 | 5.1003766 | down | lnc-ATF3-1 | LNCipedia lincRNA (lnc-ATF3-1), lincRNA [lnc-ATF3-1:4] |  |
| A_22_P00011886 | 0.04672591 | 5.0963335 | down | PIGL | phosphatidylinositol glycan anchor biosynthesis, class L [Source:HGNC Symbol;Acc:HGNC:8966] [ENST00000431149] | Hs.656332 |
| A_21_P0001911 | 0.04271877 | 5.001664 | down | NIFK-AS1 | Homo sapiens NIFK antisense RNA 1 (NIFK-AS1), transcript variant 2, long non-coding RNA [NR_037858] | Hs.712685 |
| A_22_P00011711 | 0.00521857 | 4.896274 | down | EMX2OS | Homo sapiens EMX2 opposite strand/antisense RNA (EMX2OS), long non-coding RNA [NR_002791] | Hs.312592 |
| A_23_P356152 | 0.01566199 | 4.877716 | down | MAPK8 | Homo sapiens mitogen-activated protein kinase 8 (MAPK8), transcript variant JNK1-b2, mRNA [NM_001278547] | Hs.138211 |
| A_22_P00016525 | 0.027470268 | 4.8727202 | down | LOC102724811 | PREDICTED: Homo sapiens uncharacterized LOC102724811 (LOC102724811), transcript variant X2, misc_RNA [XR_429170] | |
| A_21_P0012051 | 0.019299585 | 4.863487 | down | 14-Sep | Homo sapiens septin 14 (SEPT14), mRNA [NM_207366] | Hs.453629 |
| A_23_P170581 | 0.023818085 | 4.846749 | down | ZNF778 | zinc finger protein 778 [Source:HGNC Symbol;Acc:HGNC:26479] [ENST00000620195] | Hs.744608 |
| A_23_P85922 | 0.014714517 | 4.836803 | down | BMP8A | Homo sapiens bone morphogenetic protein 8a (BMP8A), mRNA [NM_181809] | Hs.472497 |
| A_21_P0010988 | 0.010901278 | 4.818269 | down | XLOC_l2_002423 | BROAD Institute lincRNA (XLOC_l2_002423), lincRNA [TCONS_l2_00004688] | |
| A_23_P57658 | 0.049079947 | 4.6743293 | down | HRASLS | Homo sapiens HRAS-like suppressor (HRASLS), mRNA [NM_020386] | Hs.36761 |
| A_22_P00008669 | 0.02349665 | 4.6295195 | down |  | Q7NTC3_CHRVO (Q7NTC3) Flagellar M-ring protein, partial (5%) [THC2684200] | |
| A_22_P00024338 | 0.02146446 | 4.627489 | down |  | 17000600254284 GRN_PREHEP Homo sapiens cDNA 5', mRNA sequence [CN294846] | Hs.583908 |
| A_23_P126844 | 0.025357246 | 4.5037518 | down | TNFRSF25 | Homo sapiens tumor necrosis factor receptor superfamily, member 25 (TNFRSF25), transcript variant 1, mRNA [NM_148965] | Hs.462529 |
| A_21_P0012126 | 0.037851315 | 4.490074 | down | XLOC_l2_008667 | BROAD Institute lincRNA (XLOC_l2_008667), lincRNA [TCONS_l2_00016333] | |
| A_33_P3876192 | 0.039702326 | 4.4775333 | down | IGLV1-44 | AGENCOURT_8487616 NIH_MGC_113 Homo sapiens cDNA clone IMAGE:6300723 5', mRNA sequence [BQ708343] | Hs.655198 |
| A_22_P00001509 | 0.046852253 | 4.422817 | down | lnc-ARHGAP17-3 | LNCipedia lincRNA (lnc-ARHGAP17-3), lincRNA [lnc-ARHGAP17-3:1] |  |
| A_21_P0009216 | 0.042001273 | 4.377751 | down | lnc-PFAS-1 | LNCipedia lincRNA (lnc-PFAS-1), lincRNA [lnc-PFAS-1:2] |  |
| A_33_P3308872 | 0.001388328 | 4.376542 | down | POM121L1P | Homo sapiens POM121 transmembrane nucleoporin-like 1, pseudogene (POM121L1P), non-coding RNA [NR_024591] | Hs.367764 |
| A_24_P272313 | 0.001343653 | 4.362949 | down | KIAA1211L | Homo sapiens KIAA1211-like (KIAA1211L), mRNA [NM_207362] | Hs.469398 |
| A_22_P00011505 | 0.031493615 | 4.3367815 | down | LOC101929625 | Homo sapiens uncharacterized LOC101929625 (LOC101929625), long non-coding RNA [NR_109880] | Hs.542413 |
| A_33_P3244269 | 0.024414467 | 4.3366604 | down |  |  |  |
| A_32_P200238 | 0.006559895 | 4.257871 | down | UCA1 | Homo sapiens urothelial cancer associated 1 (non-protein coding) (UCA1), long non-coding RNA [NR_015379] | Hs.644234 |
| A_22_P00023199 | 0.018220048 | 4.2489758 | down | LOC101928405 | PREDICTED: Homo sapiens uncharacterized LOC101928405 (LOC101928405), ncRNA [XR_241610] | Hs.589983 |
| A_22_P00010005 | 0.027108477 | 4.237448 | down |  | Homo sapiens cDNA FLJ11009 fis, clone PLACE1003108. [AK001871] | Hs.552711 |
| A_22_P00002310 | 0.027067628 | 4.1895957 | down | lnc-C11orf36-2 | LNCipedia lincRNA (lnc-C11orf36-2), lincRNA [lnc-C11orf36-2:1] |  |
| A_32_P715038 | 0.027439766 | 4.16981 | down |  | Homo sapiens connexin mRNA, complete cds. [AF251048] |  |
| A_21_P0007916 | 0.042363673 | 4.140993 | down | DNAJC3-AS1 | DNAJC3 antisense RNA 1 (head to head) [Source:HGNC Symbol;Acc:HGNC:39808] [ENST00000499499] | Hs.594844 |
| A_19_P00321360 | 0.030180665 | 4.139564 | down | LOC101927136 | Homo sapiens uncharacterized LOC101927136 (LOC101927136), long non-coding RNA [NR_110840] | Hs.509788 |
| A_33_P3282261 | 0.04758517 | 4.100474 | down | XLOC_l2_000001 | BROAD Institute lincRNA (XLOC_l2_000001), lincRNA [TCONS_l2_00000001] | Hs.738866 |
| A_19_P00800206 | 0.026915992 | 4.0920954 | down | LOC400958 | Homo sapiens uncharacterized LOC400958 (LOC400958), long non-coding RNA [NR_036586] | Hs.591565 |
| A_21_P0009108 | 0.038717687 | 4.072882 | down | lnc-CDH5-3 | LNCipedia lincRNA (lnc-CDH5-3), lincRNA [lnc-CDH5-3:2] |  |
| A_22_P00001115 | 0.041629564 | 4.04148 | down |  |  |  |
| A_22_P00024184 | 0.014616395 | 4.036825 | down |  |  |  |
| A_21_P0003654 | 0.005236103 | 4.031528 | down | lnc-CCRN4L-8 | LNCipedia lincRNA (lnc-CCRN4L-8), lincRNA [lnc-CCRN4L-8:1] |  |
| A_22_P00008551 | 0.042626962 | 4.0064607 | down |  | RST21962 Athersys RAGE Library Homo sapiens cDNA, mRNA sequence [BG202600] | Hs.736699 |
| A_23_P65506 | 0.004387803 | 3.9606655 | down | SPTB | Homo sapiens spectrin, beta, erythrocytic (SPTB), transcript variant 2, mRNA [NM_000347] | Hs.417303 |
| A_23_P104224 | 0.033047568 | 3.9573214 | down | A1CF | Homo sapiens APOBEC1 complementation factor (A1CF), transcript variant 3, mRNA [NM_138933] | Hs.282795 |
| A_22_P00006268 | 0.048132043 | 3.9522746 | down | LINC00452 | Homo sapiens long intergenic non-protein coding RNA 452 (LINC00452), mRNA [NM_001278674] | Hs.740982 |
| A_21_P0008297 | 0.034821045 | 3.9339974 | down | lnc-RNASE6-2 | LNCipedia lincRNA (lnc-RNASE6-2), lincRNA [lnc-RNASE6-2:1] |  |
| A_21_P0002098 | 0.035254557 | 3.869932 | down |  | DA677089 NETRP2 Homo sapiens cDNA clone NETRP2008290 5', mRNA sequence [DA677089] | Hs.580182 |
| A_24_P148499 | 0.00892883 | 3.8544571 | down | CASP8 | Homo sapiens caspase 8, apoptosis-related cysteine peptidase (CASP8), transcript variant E, mRNA [NM_033358] | Hs.599762 |
| A_33_P3268612 | 0.02207192 | 3.8401175 | down | IFNLR1 | Homo sapiens interferon, lambda receptor 1 (IFNLR1), transcript variant 1, mRNA [NM_170743] | Hs.221375 |
| A_21_P0014541 | 0.024900839 | 3.835405 | down |  |  |  |
| A_33_P3209703 | 0.009174613 | 3.832141 | down |  |  |  |
| A_33_P3415207 | 0.010139729 | 3.8284836 | down | MPV17 | MpV17 mitochondrial inner membrane protein [Source:HGNC Symbol;Acc:HGNC:7224] [ENST00000616707] | Hs.75659 |
| A_22_P00010394 | 0.017805716 | 3.7908456 | down | LOC100507661 | Homo sapiens uncharacterized LOC100507661 (LOC100507661), long non-coding RNA [NR_109989] | Hs.65918 |
| A_33_P3385842 | 0.029218113 | 3.7675967 | down | CCDC7 | Homo sapiens coiled-coil domain containing 7 (CCDC7), transcript variant 1, mRNA [NM_145023] | Hs.585464 |
| A_22_P00004675 | 0.021040922 | 3.7548707 | down | LINC01566 | Homo sapiens long intergenic non-protein coding RNA 1566 (LINC01566), transcript variant 2, long non-coding RNA [NR_027080] | Hs.385772 |
| A_23_P90980 | 0.026146103 | 3.7451682 | down | NEU2 | Homo sapiens sialidase 2 (cytosolic sialidase) (NEU2), mRNA [NM_005383] | Hs.532681 |
| A_33_P3360227 | 0.020024246 | 3.717241 | down |  | DC397705 TESTI2 Homo sapiens cDNA clone TESTI2031480 5', mRNA sequence [DC397705] | Hs.740139 |
| A_32_P124245 | 0.011954178 | 3.7149382 | down | OLIG3 | Homo sapiens oligodendrocyte transcription factor 3 (OLIG3), mRNA [NM_175747] | Hs.195398 |
| A_23_P897 | 0.025368448 | 3.7142377 | down | C1orf116 | Homo sapiens chromosome 1 open reading frame 116 (C1orf116), transcript variant 1, mRNA [NM_023938] | Hs.32417 |
| A_33_P6818008 | 0.011082709 | 3.7111676 | down | lnc-GGCT-1 | LNCipedia lincRNA (lnc-GGCT-1), lincRNA [lnc-GGCT-1:6] | Hs.561708 |
| A_24_P219053 | 0.003932514 | 3.6720562 | down | GPALPP1 | GPALPP motifs containing 1 [Source:HGNC Symbol;Acc:HGNC:20298] [ENST00000497558] | Hs.744143 |
| A_22_P00009556 | 0.047972646 | 3.64688 | down | lnc-MAP3K6-1 | LNCipedia lincRNA (lnc-MAP3K6-1), lincRNA [lnc-MAP3K6-1:1] |  |
| A_33_P3269854 | 0.019325722 | 3.635015 | down |  |  |  |
| A_33_P3307147 | 0.04617821 | 3.6057649 | down | GRIK2 | Homo sapiens glutamate receptor, ionotropic, kainate 2 (GRIK2), transcript variant 3, mRNA [NM_001166247] | Hs.98262 |
| A_22_P00007562 | 0.03575082 | 3.6018329 | down | lnc-HBEGF-1 | DA331166 BRHIP3 Homo sapiens cDNA clone BRHIP3028838 5', mRNA sequence [DA331166] | Hs.582175 |
| A_33_P3333890 | 0.014652976 | 3.593086 | down | LCORL | Homo sapiens ligand dependent nuclear receptor corepressor-like (LCORL), transcript variant 1, mRNA [NM_001166139] | Hs.446201 |
| A_33_P3251640 | 0.038413774 | 3.5847352 | down | LINC00663 | Homo sapiens long intergenic non-protein coding RNA 663 (LINC00663), long non-coding RNA [NR_026956] | Hs.665307 |
| A_22_P00017617 | 0.03043552 | 3.5797102 | down | lnc-WFIKKN2-1 | ALU1_HUMAN (P39188) Alu subfamily J sequence contamination warning entry, partial (19%) [THC2589040] | |
| A_23_P322 | 0.010739853 | 3.5261393 | down | EFNA4 | Homo sapiens ephrin-A4 (EFNA4), transcript variant 3, mRNA [NM_182690] | Hs.449913 |
| A_33_P3362562 | 0.034912594 | 3.494207 | down | RPS6KB1 | Homo sapiens ribosomal protein S6 kinase, 70kDa, polypeptide 1 (RPS6KB1), transcript variant 3, mRNA [NM_001272043] | Hs.463642 |
| A_23_P412321 | 0.031245185 | 3.472201 | down | CCR5 | Homo sapiens chemokine (C-C motif) receptor 5 (gene/pseudogene) (CCR5), transcript variant A, mRNA [NM_000579] | Hs.450802 |
| A_33_P3382588 | 0.0469492 | 3.4492502 | down | WNT1 | Homo sapiens wingless-type MMTV integration site family, member 1 (WNT1), mRNA [NM_005430] | Hs.248164 |
| A_23_P163227 | 0.026724849 | 3.4415312 | down | CKMT1A | Homo sapiens creatine kinase, mitochondrial 1A (CKMT1A), mRNA [NM_001015001] | Hs.741420 |
| A_33_P3347035 | 0.034488387 | 3.4250028 | down |  | Homo sapiens cDNA clone IMAGE:7262526, with apparent retained intron. [BC069659] | Hs.655345 |
| A_21_P0014503 | 0.03563881 | 3.4147422 | down | INTS6-AS1 | Homo sapiens INTS6 antisense RNA 1 (INTS6-AS1), long non-coding RNA [NR_103812] | Hs.594897 |
| A_33_P3372501 | 0.04965667 | 3.3979354 | down | PPP1R13B | Homo sapiens protein phosphatase 1, regulatory subunit 13B (PPP1R13B), mRNA [NM_015316] | Hs.709297 |
| A_21_P0001304 | 0.04646449 | 3.3818004 | down | lnc-PRKAA2-2 | LNCipedia lincRNA (lnc-PRKAA2-2), lincRNA [lnc-PRKAA2-2:1] |  |
| A_33_P3313695 | 0.040323433 | 3.3654482 | down |  |  |  |
| A_21_P0007556 | 0.021394 | 3.3544142 | down | LOC101927292 | Homo sapiens uncharacterized LOC101927292 (LOC101927292), long non-coding RNA [NR_110048] | Hs.129345 |
| A_23_P259207 | 0.013435027 | 3.32425 | down | THNSL2 | Homo sapiens threonine synthase-like 2 (S. cerevisiae) (THNSL2), transcript variant 1, mRNA [NM_018271] | Hs.740434 |
| A_24_P27977 | 0.035868376 | 3.2621667 | down | TRPM2 | Homo sapiens transient receptor potential cation channel, subfamily M, member 2 (TRPM2), transcript variant 1, mRNA [NM_003307] | Hs.369759 |
| A_33_P3213645 | 0.005538345 | 3.2182367 | down | ERN2 | Homo sapiens endoplasmic reticulum to nucleus signaling 2 (ERN2), mRNA [NM_033266] | Hs.592041 |
| A_22_P00001282 | 0.013793597 | 3.159851 | down | LOC102724312 | Homo sapiens uncharacterized LOC102724312 (LOC102724312), transcript variant 1, long non-coding RNA [NR_125994] | |
| A_21_P0001444 | 0.026231721 | 3.1304905 | down | lnc-EXO1-1 | LNCipedia lincRNA (lnc-EXO1-1), lincRNA [lnc-EXO1-1:1] |  |
| A_21_P0007374 | 3.86E-04 | 3.093975 | down | lnc-PRKRIR-1 | Homo sapiens cDNA FLJ30156 fis, clone BRACE2000487. [AK054718] | Hs.523913 |
| A_21_P0001037 | 0.012593643 | 3.064984 | down |  | long intergenic non-protein coding RNA 1037 [Source:HGNC Symbol;Acc:HGNC:49025] [ENST00000444887] | Hs.568709 |
| A_33_P3366156 | 0.017865269 | 3.0562217 | down | SPATA9 | Homo sapiens spermatogenesis associated 9 (SPATA9), transcript variant 1, mRNA [NM_031952] | Hs.50499 |
| A_21_P0013194 | 0.003989573 | 3.0345602 | down | LOC729737 | Homo sapiens uncharacterized LOC729737 (LOC729737), long non-coding RNA [NR_039983] | Hs.728864 |
| A_23_P22761 | 0.03371295 | 3.0332875 | down | SHOX | Homo sapiens short stature homeobox (SHOX), transcript variant 2, mRNA [NM_006883] | Hs.105932 |
| A_33_P3262167 | 0.00130897 | 3.0317085 | down |  |  |  |
| A_32_P479743 | 0.009958917 | 2.98289 | down | LINC00593 | Homo sapiens long intergenic non-protein coding RNA 593 (LINC00593), long non-coding RNA [NR_026764] | Hs.569502 |
| A_22_P00015718 | 0.016900124 | 2.9760048 | down | lnc-SYT16-1 | Homo sapiens cDNA FLJ31085 fis, clone IMR321000037. [AK055647] | Hs.713240 |
| A_22_P00019447 | 0.013805323 | 2.9541054 | down | lnc-GPR78-1 | LNCipedia lincRNA (lnc-GPR78-1), lincRNA [lnc-GPR78-1:1] |  |
| A_21_P0013332 | 0.0323668 | 2.928015 | down |  | speedy/RINGO cell cycle regulator family member E8, pseudogene [Source:HGNC Symbol;Acc:HGNC:33771] [ENST00000571071] | Hs.571275 |
| A_23_P11397 | 0.04457634 | 2.8835726 | down | TTTY13 | Homo sapiens testis-specific transcript, Y-linked 13 (non-protein coding) (TTTY13), long non-coding RNA [NR_001537] | Hs.615432 |
| A_22_P00003295 | 0.010501771 | 2.8779445 | down | STARD4-AS1 | Homo sapiens STARD4 antisense RNA 1 (STARD4-AS1), long non-coding RNA [NR_040093] | Hs.745061 |
| A_21_P0012866 | 0.014896685 | 2.84648 | down | XLOC_l2_011872 | BROAD Institute lincRNA (XLOC_l2_011872), lincRNA [TCONS_l2_00022657] | |
| A_33_P3795524 | 0.017153366 | 2.8174706 | down | LRRC2-AS1 | Homo sapiens LRRC2 antisense RNA 1 (LRRC2-AS1), long non-coding RNA [NR_073385] | |
| A_23_P7866 | 0.041878823 | 2.7309837 | down | GPR115 | Homo sapiens G protein-coupled receptor 115 (GPR115), mRNA [NM_153838] | Hs.150131 |
| A_33_P3255531 | 0.035375424 | 2.7271118 | down | LOC100129216 | Homo sapiens beta-defensin 131-like (LOC100129216), mRNA [NM_001242853] | Hs.740246 |
| A_21_P0005633 | 0.00890217 | 2.6909354 | down | lnc-BAALC-1 | LNCipedia lincRNA (lnc-BAALC-1), lincRNA [lnc-BAALC-1:1] |  |
| A_21_P0008995 | 0.032224115 | 2.6348734 | down | lnc-BANP-1 | LNCipedia lincRNA (lnc-BANP-1), lincRNA [lnc-BANP-1:2] |  |
| A_23_P343963 | 0.032460254 | 2.634489 | down | FAM83F | Homo sapiens family with sequence similarity 83, member F (FAM83F), mRNA [NM_138435] | Hs.197680 |
| A_22_P00018850 | 0.041014016 | 2.6311357 | down | LOC101927207 | Homo sapiens uncharacterized LOC101927207 (LOC101927207), long non-coding RNA [NR_110805] | Hs.638402 |
| A_22_P00003447 | 0.018481933 | 2.6173465 | down |  |  |  |
| A_33_P3394105 | 0.033703525 | 2.5647354 | down | SPATA2L | Homo sapiens spermatogenesis associated 2-like (SPATA2L), mRNA [NM_152339] | Hs.374556 |
| A_21_P0009510 | 0.010632364 | 2.5123918 | down | lnc-KCNG2-1 | LNCipedia lincRNA (lnc-KCNG2-1), lincRNA [lnc-KCNG2-1:1] |  |
| A_19_P00319509 | 0.032643374 | 2.468401 | down | LINC00698 | Homo sapiens long intergenic non-protein coding RNA 698 (LINC00698), long non-coding RNA [NR_027104] | Hs.211125 |
| A_32_P212095 | 0.025926026 | 2.4181077 | down | C17orf105 | Homo sapiens chromosome 17 open reading frame 105 (C17orf105), mRNA [NM_001136483] | Hs.252707 |
| A_23_P44643 | 0.049791068 | 2.4130278 | down | ANAPC7 | Homo sapiens anaphase promoting complex subunit 7 (ANAPC7), transcript variant 1, mRNA [NM_016238] | Hs.719935 |
| A_21_P0005800 | 0.04115396 | 2.3980672 | down | lnc-RP1L1-1 | LNCipedia lincRNA (lnc-RP1L1-1), lincRNA [lnc-RP1L1-1:1] |  |
| A_19_P00319409 | 0.04918521 | 2.3845403 | down | ERVMER34-1 | Homo sapiens endogenous retrovirus group MER34, member 1 (ERVMER34-1), transcript variant 1, mRNA [NM_024534] | Hs.363087 |
| A_22_P00015227 | 0.044669364 | 2.358386 | down |  | RST36459 Athersys RAGE Library Homo sapiens cDNA, mRNA sequence [BG216764] | Hs.574637 |
| A_23_P150979 | 0.012891697 | 2.324954 | down | MUCL1 | Homo sapiens mucin-like 1 (MUCL1), mRNA [NM_058173] | Hs.348419 |
| A_23_P26810 | 0.004134723 | 2.303505145 | down | TP53 | Homo sapiens tumor protein p53 (TP53), transcript variant 1, mRNA [NM_000546] | Hs.437460 |
| A_21_P0010743 | 0.003150593 | 2.2850635 | down |  |  | Hs.149726 |
| A_24_P704878 | 0.0108208 | 2.250366 | down |  |  |  |
| A_33_P3297302 | 0.032801885 | 2.2282324 | down | ELMOD3 | Homo sapiens ELMO/CED-12 domain containing 3 (ELMOD3), transcript variant 2, mRNA [NM_001135021] | Hs.269990 |
| A_21_P0006005 | 0.010497571 | 2.2279391 | down |  | PREDICTED: Homo sapiens RGP1 retrograde golgi transport homolog (S. cerevisiae) (RGP1), transcript variant X1, mRNA [XM_006716895] | |
| A_23_P114210 | 2.65E-04 | 2.2073417 | down | POU3F4 | Homo sapiens POU class 3 homeobox 4 (POU3F4), mRNA [NM_000307] | Hs.2229 |
| A_21_P0001088 | 0.002711047 | 2.190557 | down | lnc-AL590822.1-1 | UI-H-FE1-beg-g-15-0-UI.s1 NCI_CGAP_FE1 Homo sapiens cDNA clone UI-H-FE1-beg-g-15-0-UI 3', mRNA sequence [CA425772] | Hs.553310 |
| A_33_P3238148 | 0.013955968 | 2.1752472 | down | LRRD1 | leucine-rich repeats and death domain containing 1 [Source:HGNC Symbol;Acc:HGNC:34300] [ENST00000343318] | Hs.671729 |
| A_21_P0005029 | 0.021696474 | 2.1692488 | down | lnc-SUPT3H-1 | LNCipedia lincRNA (lnc-SUPT3H-1), lincRNA [lnc-SUPT3H-1:1] |  |
| A_33_P3253361 | 0.03963922 | 2.1540759 | down |  | Homo sapiens cDNA FLJ45509 fis, clone BRTHA2020811. [AK127417] |  |
| A_24_P77947 | 0.024301924 | 2.0919642 | down | CCDC132 | Homo sapiens coiled-coil domain containing 132 (CCDC132), transcript variant 2, mRNA [NM_024553] | Hs.202424 |
| A_33_P3306843 | 0.031149976 | 2.0799346 | down | ZNF836 | Homo sapiens zinc finger protein 836 (ZNF836), mRNA [NM_001102657] | Hs.631584 |
| A_22_P00002921 | 0.012100505 | 2.0374112 | down | lnc-C5orf25-2 | LNCipedia lincRNA (lnc-C5orf25-2), lincRNA [lnc-C5orf25-2:1] |  |
| A_22_P00001448 | 0.028015744 | 2.0230637 | down | PRKCA-AS1 | Homo sapiens PRKCA antisense RNA 1 (PRKCA-AS1), long non-coding RNA [NR_110822] | Hs.668027 |
| A_23_P147070 | 0.014654144 | 2.0185628 | down | SUN1 | Sad1 and UNC84 domain containing 1 [Source:HGNC Symbol;Acc:HGNC:18587] [ENST00000340926] | |
| A_33_P6817530 | 0.011387959 | 2.0098143 | down | PCBP1-AS1 | Homo sapiens PCBP1 antisense RNA 1 (PCBP1-AS1), long non-coding RNA [NR_033872] | Hs.716915 |
| A_23_P142250 | 0.02012296 | 1.9969853 | down | SLC7A9 | Homo sapiens solute carrier family 7 (amino acid transporter light chain, bo,+ system), member 9 (SLC7A9), transcript variant 1, mRNA [NM_014270] | Hs.743345 |
| A_33_P3412488 | 0.001913302 | 1.987705 | down |  |  |  |
| A_23_P44648 | 0.0053458 | 1.9261948 | down | ADAMTS12 | Homo sapiens ADAM metallopeptidase with thrombospondin type 1 motif, 12 (ADAMTS12), mRNA [NM_030955] | Hs.12680 |
| A_21_P0014694 | 0.022852652 | 1.8937618 | down | LOC101929910 | PREDICTED: Homo sapiens nuclear pore complex-interacting protein family member B4-like (LOC101929910), misc_RNA [XR_433240] | Hs.720286 |
| A_21_P0005320 | 0.04957628 | 1.7655623 | down | lnc-EPHB4-1 | LNCipedia lincRNA (lnc-EPHB4-1), lincRNA [lnc-EPHB4-1:1] |  |
| A_21_P0000942 | 0.040267773 | 1.6843172 | down | lnc-RER1-1 | LNCipedia lincRNA (lnc-RER1-1), lincRNA [lnc-RER1-1:1] |  |
| A_22_P00016700 | 0.032435626 | 1.6326216 | down |  | long intergenic non-protein coding RNA 467 [Source:HGNC Symbol;Acc:HGNC:28227] [ENST00000423222] | Hs.550012 |
| A_22_P00002568 | 0.028173547 | 1.5945395 | down | lnc-C17orf89-1 | LNCipedia lincRNA (lnc-C17orf89-1), lincRNA [lnc-C17orf89-1:2] | Hs.620720 |
| A_33_P3404588 | 0.03832348 | 1.5914202 | down | FGD4 | Homo sapiens FYVE, RhoGEF and PH domain containing 4 (FGD4), mRNA [NM_139241] | Hs.117835 |
| A_33_P3242909 | 0.004473347 | 1.5515002 | down | ZNF563 | Homo sapiens zinc finger protein 563 (ZNF563), mRNA [NM_145276] | Hs.663510 |
